# Analysis of culturable airborne fungi in outdoor environments in Tianjin, China

**DOI:** 10.1186/s12866-021-02205-2

**Published:** 2021-05-02

**Authors:** Yumna Nageen, Michael Dare Asemoloye, Sergei Põlme, Xiao Wang, Shihan Xu, Pramod W. Ramteke, Lorenzo Pecoraro

**Affiliations:** 1 School of Pharmaceutical Science and Technology, Tianjin University, 92 Weijin Road, Nankai District, 300072 Tianjin, China; 2 Institute of Ecology and Earth Sciences, University of Tartu, 14A Ravila, 50411 Tartu, Estonia; 3 Faculty of Life Sciences, Mandsaur University, 458001 Mandsaur, India

**Keywords:** Airborne fungi, Biodiversity, Environmental factors, Outdoor environments

## Abstract

**Background:**

Fungal spores dispersed in the atmosphere may become cause of different pathological conditions and allergies for human beings. A number of studies have been performed to analyze the diversity of airborne fungi in different environments worldwide, and in particular in many urban areas in China. We investigated, for the first time, the diversity, concentration and distribution of airborne fungi in Tianjin city. We sampled 8 outdoor environments, using open plate method, during a whole winter season. Isolated fungi were identified by morphological and molecular analysis. Environmental factors which could influence the airborne fungi concentration (temperature, humidity, wind speed, and air pressure) were monitored and analyzed. The effect of different urban site functions (busy areas with high traffic flow and commercial activities vs. green areas) on airborne fungal diversity was also analyzed.

**Results:**

A total of 560 fungal strains, belonging to 110 species and 49 genera of Ascomycota (80 %), Basidiomycota (18 %), and Mucoromycota (2 %) were isolated in this study. The dominant fungal genus was *Alternaria* (22 %), followed by *Cladosporium* (18.4 %), *Naganishia* (14.1 %), *Fusarium* (5.9 %), *Phoma* (4.11 %), and *Didymella* (4.8 %). A fungal concentration ranging from 0 to 3224.13 CFU m^− 3^ was recorded during the whole study. Permutational multivariate analysis showed that the month was the most influential factor for airborne fungal community structure, probably because it can be regarded as a proxy of environmental variables, followed by wind speed. The two analyzed environments (busy vs. green) had no detectable effect on the air fungal community, which could be related to the relatively small size of parks in Tianjin and/or to the study season.

**Conclusions:**

Our study shed light on the highly diverse community of airborne fungi characterizing the outdoor environments of Tianjin, and clarified the role that different environmental factors played in shaping the analyzed fungal community. The dominant presence of fungi with potential hazardous effect on human health, such as *Alternaria, Cladosporium* and *Naganishia*, deserves further attention. Our results may represent a valuable source of information for air quality monitoring, microbial pollution control, and airborne diseases prevention.

**Supplementary Information:**

The online version contains supplementary material available at 10.1186/s12866-021-02205-2.

## Background

Fungi are one of the most abundant, widely distributed and pervasive group of organisms on Earth [1]. These organisms are ubiquitous in nature, playing different roles in the environment as symbionts, saprotrophs or parasites, which enable them to colonize diverse habitats [2]. Fungal presence in the ecosystem reaches tremendous levels, being fungi estimated to comprise approximately 25 % of the global biomass [3, 4]. Spores of different fungal species are dispersed in the atmosphere hence, airborne fungi play a critical role in air pollution [5], which can alter biotic and/or abiotic factors of the environment, thus affecting human health [6]. The diversity and concentration of airborne fungi in a certain area depend on environmental condition, human activity and availability of substrate for fungal growth [7, 8]. Fungal invasion of human body may lead to many pathological conditions and allergies. Until now, approximately 150 allergy causing fungal *taxa* have been identified [9], while about 10 % of the world population is suspected to have fungal allergies [10–13]. The myriad health effects of fungi on humans, plants and livestock are well recognized [14], including severe diseases of human respiratory system [1, 13]. Effects of airborne fungi on human health have stimulated researchers to study the diversity of aeroallergens, which has led to the identification of nearly 80 genera of fungi mainly responsible for respiratory ailments or anomalies [10, 15–23]. Over 100 fungal species are known to cause serious animal and human infections [2, 24]. *Alternaria, Penicillium, Aspergillus* and *Cladosporium* are among the airborne fungal genera responsible for allergic rhinitis or asthma [5, 8, 10, 25, 26]. Airborne fungi are also suspected to be one of the causes of lung cancer. Aflatoxin B_1_ produced by *Aspergillus flavus* was found to cause liver and lung cancer when the fungus entered the respiratory tract by ingestion or inhalation [27–31]. Some of the less severe infectious diseases caused by fungi include aspergillosis [32], hypersensitivity diseases, such as hypersensitivity pneumonitis and asthma [33, 34], and toxicosis reactions, including acute systemic toxicosis [35]. *Aspergillus fumigatus* is responsible for about 90 % of all the invasive aspergillosis, while *A. niger* causes a range of invasive pulmonary diseases [36]. Exposure to the airborne fungi was found to be associated with respiratory allergy symptoms, asthma exacerbation and asthma complication leading to respiratory failure and death [37–39]. Knowledge about the identity and relative frequencies of airborne fungi in the environment is necessary to evaluate potential health hazards [5].

Nowadays, the global research on aeroallergens is considered of primary importance by scholars and researchers, resulting in a large body of literature available on airborne fungi [6, 8, 40]. Numerous scientific studies have been conducted on outdoor microorganisms in China and reported on national and international forums over the past several years. Airborne fungi concentrations are monitored in many Chinese cities, including Baoding [41], Beijing [26, 42–44], Hangzhou [40], Nanjing [45], Shenzhen [46], Tainan [1, 47], and Xi’an [48, 49]. These studies have increased our basic knowledge on the diversity of airborne fungi in the environment and have provided data on some important issues related to public health [40]. The study of disease-causing airborne fungi can help in the medical evaluations, assessment of health issues, development of remedies, and assist in the proactive air quality monitoring [5].

Air pollution does not only affect the human health by creating pathological conditions, allergies and other health related problems, but also has a negative influence on the socioeconomic development process to a significant level in most of the northern cities of China [44]. Tianjin is the third biggest municipality and the Northern China’s leading manufacturing centre and port [50]. Currently, there are no data available on the diversity of airborne fungi comparing the different urban environments in Tianjin. Our study aimed at contributing to the knowledge of airborne fungi in Tianjin, with the assessment of the diversity of culturable strains, using the open plate sampling method in different outdoor environments. We described diversity, concentration, distribution, and relative frequency of culturable airborne fungi in eight different sampling sites in Tianjin. We also analyzed the influence of several environmental factors on the studied fungal community. Results from the present research could provide support in pollution control and environmental protection in urban environments.

## Results

**Fig. 1:**
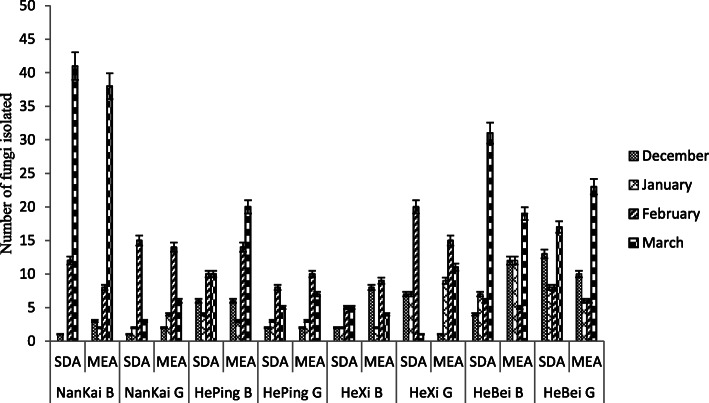
Total fungal strains isolated from each location

A total of 560 different fungal strains belonging to 110 species and 49 genera of Ascomycota (80 %), Basidiomycota (18 %), and Mucoromycota (2 %) were identified from the 8 analyzed sites in 4 urban districts in Tianjin (Fig. 1, Supplementary Table S3). Among the dominant fungal genera, approximately 22 % of the total identified strains belonged to the genus *Alternaria*, followed by *Cladosporium* 18.4 %, *Naganishia* 14.1 %, *Fusarium* 5.9 %, *Phoma* 4.11 %, and *Didymella* 4.8 % (Supplementary Table S4). The most frequently occurring fungal species were *Alternaria alternata* (13.4 %), *Cladosporium cladosporioides* (9.6 %), *Alternaria tenuissima* (7.5 %), *Naganishia albida* (6.4 %), and *N. globosa* (6.1 %), followed by *Didymella pedeiae* (2.7 %), *Fusarium verticillioides* (2.7 %), *F. equiseti* (2.3 %), *C. tenuissimum* (2.3 %), *Valsa sordida* (2.3 %), *C. anthropophilum* (1.8 %) (Supplementary Table S5). The genus *Cladosporium* showed the highest species richness of the whole airborne fungal community, with 13 identified species, 12 % of total recorded *taxa*. *Alternaria*, represented by 8 species (7 %), was the second most species-rich genus, followed by *Naganishia* (7 species, 6 %), while 6 species (5.5 %) were found to represent each of the genera *Aspergillus, Didymella, Fusarium*, and *Phoma*.

**Fig. 2:**
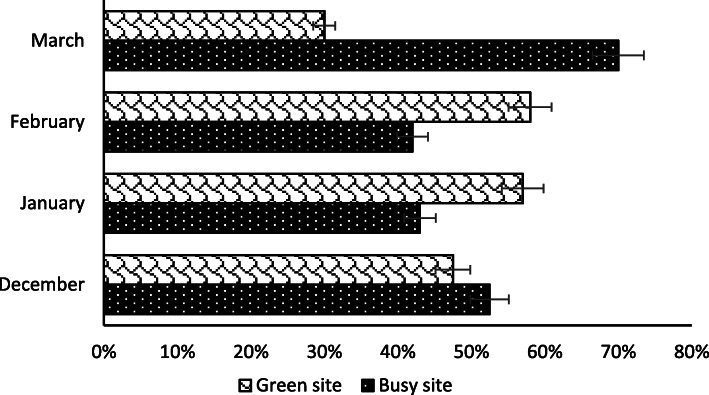
Isolated strains based on site

**Fig. 3:**
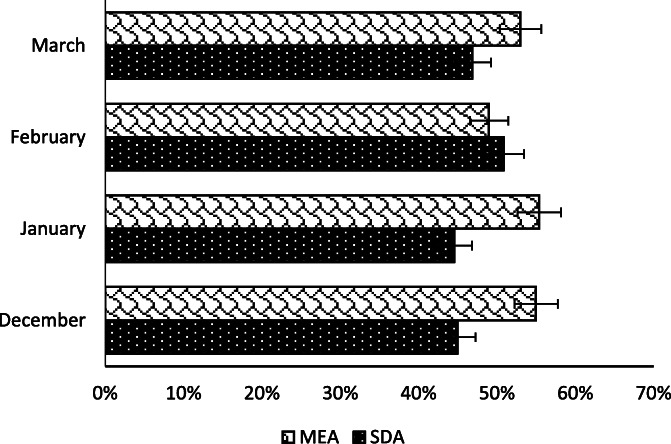
Isolated strains based on growth media

The highest number of strains was recorded in March (241), followed by February (165), while January and December sampling yielded a considerably lower number of isolates, with a total of 74 and 80 strains respectively (Supplementary Table S2). Overall, 55.5 and 44.5 % colonies were isolated from busy and green sites, respectively (Fig. 2). Concerning the two culture media used in this study, 47.5 % colonies were isolated on SDA, whereas 52.5 % fungal strains were obtained on MEA (Fig. 3).

A varying fungal concentration was observed at each location in each month as shown in Table 1. The concentration of outdoor culturable airborne fungi ranged from 0 CFU m^− 3^ to 3224.13 CFU m^− 3^, with an average of 625 CFU m^− 3^ and 720 CFU m^− 3^ recorded for SDA and MEA media, respectively.

**Table 1:** Fungal colony average concentration detected in Tianjin outdoor environments

Sampling Month	Medium	Colony Concentration (CFU m^**-3**^)							
		NanKai B	NanKai G	HePing B	HePing G	Hexi B	Hexi G	Hebei B	Hebei G
December	SDA	78.64	78.64	471.82	157.27	157.27	550.46	314.55	1022.29
	MEA	235.91	157.27	471.82	157.27	629.1	78.64	943.65	786.37
January	SDA	0	157.27	314.55	235.91	157.27	629.1	550.46	629.1
	MEA	157.27	235.91	2359.1	235.91	157.27	707.74	943.65	471.82
February	SDA	943.65	1179.56	550.46	865.01	393.19	1572.75	471.82	629.1
	MEA	629.1	1100.92	1022.29	550.46	550.46	1494.11	550.46	471.82
March	SDA	3224.13	235.91	786.37	393.19	393.19	78.64	2437.76	1336.84
	MEA	2988.22	471.82	1572.75	550.46	314.55	865.01	1494.11	1808.66

Sampling district name (Nankai, Heping, Hexi, Hebei), site (*G* Green, *B* Busy), and isolation medium (*MEA* Malt Extract Agar, *SDA* Sabouraud Dextrose Agar) are indicated

The environmental parameters recorded in this study (Supplementary Table S1) showed different effect on the analysed fungal diversity. According to permutational multivariate analysis month had the greatest impact (Adonis: F_3,17_ = 3.57) to airborne fungal community structure, followed by wind speed (F_1,17_ = 2,13) when included simultaneously in the model (Table 2). Wind, humidity, and temperature had a similar effect when tested one by one in the model. Surprisingly, the two different environments analyzed in each district (green vs. busy) and growth media had no detectable effect on the airborne fungal community. Since culture medium did not affected fungal community structure, observations divided by the media were collapsed in order to avoid pseudoreplication in further community analyses.

**Table 2:** Permutational multivariate analysis showing the effects of location, weather and month on fungal community abundance

	Degrees of freedom	F-value	adjusted R2	*P*-value
Month	3	3.573	0.175	0.001
logWind	1	2.132	0.019	0.007
Humidity	1	1.734	0.009	0.058
logTemp	1	1.539	0.005	0.112
Site	7	1.103	0.054	0.264
Air pressure	1	1.142	0.005	0.331
Busy vs. Green Site	1	1.051	0.007	0.392
Residuals	17	0.405		
Total	31			

## Discussion

This is the first study on culturable airborne fungi in Tianjin City, China. The research project was carried out to investigate the concentration, identity and diversity of airborne fungi present in the outdoor environments of the studied city. The 4 analyzed urban districts, each comprising a green and a busy site, showed a high airborne fungal diversity, which resulted in the isolation of 560 strains belonging to 110 fungal species in 49 genera. Previous studies have shown the presence of less diverse culturable airborne fungal communities, compare to Tianjin, in other cities of China, such as Beijing, where 40 species belonging to 14 genera were recorded [26], Hangzhou, with 85 species in 21 genera [40], and Shenzhen (27 species, 18 genera) [46]. Overall, a fungal concentration ranging from 0 to 3224.13 CFU m^− 3^ was recorded across the eight different analyzed sites in Tianjin. The average concentration was 625 CFU m^− 3^ on SDA medium and 720 CFU m^− 3^ on MEA (Table 1). Many studies have reported various concentrations of airborne fungi across cities in different countries [5, 40, 51, 52], in some cases showing that the outdoor environment fungal diversity and concentration depended on the traffic flow, human activities and vegetation presence [52–54]. For example, in a study conducted in different regions of the US, Shelton et al. [5] reported a median outdoor fungal concentration of 500 CFU m^− 3^, while Kalyoncu et al. [51] found a fungal concentration of 412 CFU m^− 3^ in eight different environments within Yunusemre City, in Turkey. Numerous studies have reported on the outdoor airborne fungal concentrations in different cities of China. Fang et al. [42] observed a fungal concentration ranging from 4.8 × 10^2^ CFU m^− 3^ to 2.4 × 10^4^ CFU m^− 3^ in three different sampling sites in Beijing, whereas the concentration of culturable fungi in the air was found to range from < 12 to 8767 CFU m^− 3^ (mean value 848 CFU m^− 3^) in a study performed in Hangzhou, in four sampling locations characterized by different urban functions [40].

The most abundant fungal genera found in our analyzed environments included *Alternaria, Cladosporium*, and *Naganishia* (Supplementary Tables S4 and S5). *Alternaria* and *Cladosporium* have been similarly reported as the most common airborne fungi in other research studies performed in Nanjing, Beijing and Hangzhou [26, 40, 45]. *Alternaria* was the dominant genus recorded in Tianjin, comprising almost one fourth of all the isolated airborne fungal strains. Among the recorded *Alternaria* species, *A. alternata*, which was the most frequently occurring *taxon* of the whole study, is known as a common and widespread allergenic fungus, responsible for Immunoglobulin E (IgE) mediated respiratory diseases, specifically for asthma exacerbation [55]. Besides, 16 allergens of *A. alternata* have been overall reported so far [56, 57]. *Cladosporium* was recorded as the second most common genus in Tianjin air environments. *Cladosporium* species are mostly saprotrophic [58], but have been occasionally described as opportunistic causative agents of infections in animals and humans [59, 60]. In a study performed by Sellart et al. [61] in Spain, where nasal samples of 135 people (healthy and allergic) were analyzed, *Cladosporium* was reported as the prevalent fungal genus in nasal microbiota, being *C. herbarum* and *C. cladosporioides* the most abundant species (23.6 %). Ma et al. [62], in another clinical study carried out in China, reported *C. cladosporioides* as a cause of Phaeohyphomycotic dermatitis in giant panda. The genus *Naganishia*, showing the third highest concentration in our results, can be considered a peculiar presence in Tianjin outdoor environments, as this genus was not reported in any other study on airborne fungi conducted in China. *Naganishia albida*, which accounted for 6.4 % of total isolated strains in this work, was reported as the cause of Otomycosis [63] and superficial skin infection [64] in case studies performed in Iran. The presence of *N. albida* in Tianjin air samples deserves further attention as a potential threat for human health. Long-term monitoring of this fungal species distribution and abundance in both outdoor and indoor Tianjin city environments is recommended.

Previous studies have shown that environmental factors such as temperature, humidity, wind speed and atmospheric pressure could affect the diversity of airborne fungi in different environments [58]. The analyses performed in our work revealed that wind had the highest influence on fungal community, among the studied environmental factors. This result is in agreement with previous works showing that wind enhanced the release and dispersal of fungal spores, by favoring the removal of spores from fruit bodies and their transportation and suspension in the air [65, 66]. Other environmental factors analyzed in our study, including temperature, humidity and atmospheric pressure showed a lower, but still significant effect on diversity and concentration of airborne fungi. Looking at the numerous aeromycological studies on the influence of meteorological factors on airborne fungal concentration that have been published [65–70], the results of our work are in agreement with findings from Giri et al. [65] who observed a very significant effect of atmospheric pressure and wind speed on particulate matter concentration as compared to temperature and relative humidity. Similar results were obtained by Santiago et al. [71] who studied the effect of meteorological factors on outdoor airborne fungi, using both culture-based methods and direct microscopic analysis of fungi. In the latter work, a significant correlation between fungal colony growth and relative humidity and wind speed was recorded. Some previous studies, instead, reported a major influence of temperature and humidity on the diversity of air borne fungi. For instance, in the work performed by Fang et al. [40] in Hangzhou, air temperature was found to sustain fungal growth and germination in all seasons except winter, whereas a study carried out in Beijing, showed that both air temperature and humidity in summer and autumn were more appropriate for fungal propagation as compared to winter season [26]. A strong relationship between fungal growth and relative humidity was reported by Jones and Harrison [72], who showed that the humidity in the air could support different mycelial structures growth in fungi, ultimately resulting in the increase in fungal air-borne concentration. In a study performed in Nepal, the atmospheric pressure was found to affect the suspension, dispersal, and sedimentation of particulate matter in the air [65], which was later interpreted by Pyrri et al. [73] to support the hypothesis that high atmospheric pressure combined with elevated temperature may increase the suspension of fungal spores in the air, and prevent their sedimentation. Further studies based on large-scale sampling and long-term data collection are necessary to disentangle the effect of different environmental factors affecting the diversity and structure of air borne fungal communities.

In our study, we also tried to understand the effect of different urban district management on the outdoor fungal diversity. We did not observe any significant difference in the concentration of airborne fungi in green vs. busy sites, according to the results of statistical analysis. Different studies have previously suggested a strong association between vegetation coverage and concentration of airborne fungi in outdoor environments. For instance, a study conducted by Fang et al. [26] has shown that the airborne fungi in green areas in Beijing were more diverse than in densely populated and polluted areas. Other studies have reported that areas with more abundant presence of green plants were characterized by higher concentration of airborne fungi [26, 40, 43, 66]. In particular, Picco et al. [66] have found that the presence and growth of parasitic and saprophytic fungi in green areas were supported by phylloplane (leaf surface) availability. The contrasting results obtained from our work could be explained with the relatively small size characterizing the green areas in Tianjin, which could result in a strong influence of surrounding busy areas, thus making the fungal communities inside and outside parks similar. Besides, given that our study was performed during the winter season, when the great majority of plant did not bear leaves, due to the climatic conditions in Tianjin, it is possible that the limited phylloplane availability reduced the effect of leaf surface on fungal diversity registered in the above-mentioned previous studies [66]. Further analyses of Tianjin air bone fungi in different seasons could be useful to test the latter hypothesis. However, the percentage of total species isolated each month in our study registered a noteworthy shift in the fourth month of sampling (March 2020, Fig. 2) when busy sites showed a significantly higher fungal concentration than green sites, never observed in the previous three months. We cannot exclude that the nationally adopted lockdown, due to the COVID-19 pandemic, had some influence on the latter result. Indeed, we observed a much more intense activity and presence of people in the busy sites during the last month of sampling, when the restrictions related to the lockdown became smaller. This increased activity within the busy sites could have had some effect on the concentration of air borne fungi, although it is also possible that other environmental factors not measured in our study were involved in the observed results variation.

## Conclusions

In this study, we shed light on the highly diverse community of airborne fungi characterizing the outdoor environments of Tianjin City. We clarified the role that different environmental factors played in shaping the analyzed fungal community, and revealed the particularly strong effect of the wind on Tianjin air borne fungal diversity and concentration. Our effort to compare fungal communities in busy and green urban areas did not show substantial difference between the two investigated environments. This finding could be related to the relatively small size of parks in Tianjin and/or to the study season, hypotheses that require further studies to be tested. Among the identified cultured fungi, the dominant presence of strains belonging to *Alternaria, Cladosporium* and *Naganishia* genera may constitute an important feature of Tianjin outdoor environments, which deserves to be studied from a pathological point of view, due to the potential hazardous effect of these fungal *taxa* on human health. The results from our study may represent a valuable source of information for air quality monitoring, microbial pollution control, and airborne diseases prevention. Further studies are needed to generate additional data from long-term sampling, in order to increase our understanding of air borne fungal population dynamics and composition in Tianjin, across different seasons.

## Materials and methods

### Sampling sites

This study was conducted in Tianjin city, located in the North-Est of China (39°8’31.99"N, 117°10’36.01"E). The city was built in 1403 on the banks of Hai River at West of Bohai Sea, South of Yanshan Mountains and East to Beijing. It occupies 11,946 km squared land area with an approximated population of 13,589,078 as of 2020, distributed in 15 districts and three counties. Tianjin ranked 29th in world’s largest human agglomeration, 4th largest urban population in China and 11th in the world’s most populous city [74].

Eight sites, across 4 urban districts in Tianjin city, were investigated in this study. Two distinct sampling environments were selected in each district, one constituted by an open busy area with high commercial activities and traffic flow (busy site) and the other representative of green areas in urban parks (green site), as presented in Table 3.

**Table 3:** Detailed information of the eight selected sampling sites in Tianjin

Site	District	Location	GPS Position
Green Sites	Nan Kai	Water park	39°5’23”N, 117°9’42”E
	He Bei	Zhong Shan Park	39°9’21”N, 117°12’24”E
	He Ping	Central Park	39°7’24”N, 117°11’54”E
	He Xi	People’s Park	39°6’10”N, 117°12’44”E
Busy Sites	Nan Kai	East gate of TJU	39°6’26”N, 117°10’24”E
	He Bei	Kunwei Hospital	39°9’21”N, 117°12’28”E
	He Ping	Bin Jiang Road	39°7’22”N, 117°11’34”E
	He Xi	Tianjin Foreign Studies University	39°6’21”N, 117°12’18”E

### Sampling method and culture media

Sampling of fungi in bio-aerosols at the study sites was conducted for four consecutive months, from December 2019 to March 2020. Air samples were collected monthly, when the weather was dry and stable, approximately between 12:00–13:00 o’clock. Airborne fungi were sampled using the open plate method [2, 46, 75], by opening sterile plates containing media for 10 min at a height of 1.5 m above ground, which is representative of the human breathing zone. Minimum distance of 1 m from walls and obstacles was maintained. For each air sampling, 2 petri dishes of 9.0 cm diameter, one containing Sabouraud Dextrose Agar (SDA), another containing Malt Extract Agar (MEA) medium, amended with chloramphenicol (100 mg/L) to inhibit bacterial growth, were used. Environmental parameters including temperature, humidity, wind speed and air pressure were recorded using Huafeng-AccuWeather software at each sampling site. Exposed culture dishes were incubated at 25 °C, in the darkness, and examined for fungal colony growth every 24 h, for 5–7 days. Fungal colonies were observed and counted in each plate. Each colony was carefully picked up, and aseptically inoculated in a new plate for isolation. Fungal morphology was observed in pure cultures, and isolated strains were identified using molecular method. All isolated fungal strains were deposited in the LP Culture Collection (personal culture collection held in the laboratory of Prof. Lorenzo Pecoraro), at the School of Pharmaceutical Science and Technology, Tianjin University, Tianjin, China.

### Enumeration of fungi

After incubation and counting of fungal colonies, the concentration obtained from the samples was expressed as CFU per cubic meter of air (CFU m^− 3^). Omelyansky formula [76, 77] was used to calculate the fungal concentration in unit CFU m^− 3^:

$$\mathrm N=5\mathrm a\times104\left(\mathrm{bt}\right)^{-1}$$

where “N” is the concentration of fungi, “a” is the number of fungal colonies per plate, “b” is the total area of the plate (cm^2^); and “t” is the time duration for opening of plate (min).

### Fungal identification

The isolated fungal colonies were identified using DNA-based molecular analysis combined with microscopy. DNA extraction was performed using the cetyltrimethylammonium bromide method [78]. The amplification of fungal rRNA genes internal transcribed spacer (ITS) region was performed using the following universal primer set: ITS1 (5′-TCCGTAGGTGAACCTGCGG-3′) and ITS4 (5′TCCTCCGCTTATTGATATGC-3′) [55]. The reaction mixture for PCR (50 µl), consisted of 25.0 µl of 2 × Rapid Taq Master Mix (Vazyme Biotech Co. Ltd, Nanjing), 2 µl of forward primer (10 µM), 2 µl of reverse primer (10 µM) and 2.0 µl (20 ng DNA) of template and 19 µl of double distilled sterilized water. The amplification program was as follows: initial denaturation at 95 °C for 3 min, 30–35 cycles of 95 °C for 15 s, annealing at 60 °C for 15 s, extension at 72 °C for 15 s, followed by a final extension at 72 °C for 5 min. The PCR products were detected by gel electrophoresis using electrophoresis tank (LiuYi, Beijing) on a 1 % agarose gel. The sequences were obtained from Genewiz, Inc., China, and analyzed with the Basic Local Alignment Search Tool (BLAST) program of the National Center for Biotechnology Information, USA (http://www.ncbi.nlm.nih.gov/Blast.cgi) in order to identify the closest sequence in the NCBI database. DNA sequences were deposited in GenBank (Accession Nos. MW723617–MW724176).

Fungal morphology (branched septate hyphae, pseudohyphae, conidiophores, conidia, poroconidia, arthroconidia and sporangiosphores, etc.) was examined under Nikon ECLIPSE Ci microscope for identification of isolates following the standard taxonomic keys for different *taxa* [79–83].

### Statistical analysis

The frequency of fungal species in petri dishes per site was used in community-level analyses. Wind and temperature values were log-transformed before analyses in order to reduce heteroscedasticity. To address the relative importance of media and environmental factors on the community structure of airborne fungi, we used a multivariate ANOVA as implemented in the Adonis routine of the Vegan package of R [84]. Prior to permutational multivariate analysis, abundance data was transformed into binary format and the Bray–Curtis dissimilarity metric was used to calculate the community distance matrix. Adjusted R-squares were calculated in the final model.

In order to summarize descriptive statistics of fungal concentrations, results were calculated using Excel 2016 software. ANOVA was used for comparing fungal concentrations between different fungal samples and sampling times while the means were separated using Duncan Multiple Range Test (DMRT) in SPSS Version 20.0 (Standard Version, SPSS Inc.) software.

## Supplementary Information

**Additional file 1: Supplementary Table S1. **Environmental factors recorded in each samplingsite at the time of sampling. **Supplementary Table S2.** Total number of fungal colonies isolated from each location from December 2019 to March 2020. **Supplementary Table S3.** Airborne fungal diversity molecularly detected in Tianjin outdoor environments, from DNA extracted from isolated strains. **Supplementary Table S4.** Isolated airborne fungal genera and number of strains. **Supplementary Table S5.** Isolated air borne fungal species and number of strains

## Data Availability

All data generated or analysed during this study are included in this published article. The Fungal DNA sequences amplified during this study are available in GenBank under accessions MW723617–MW724176.
