# Supplementary material for: Analysis of culturable airborne fungi in outdoor environments in Tianjin, China
Source: BMC Microbiol. 2021 May 2;21:134. doi: 10.1186/s12866-021-02205-2 (PMC8088404; doi:10.1186/s12866-021-02205-2)
Supplement: Supplementary file 1 — Additional file 1: Supplementary Table S1. Environmental factors recorded in each samplingsite at the time of sampling. Supplementary Table S2. Total number of fungal colonies isolated from each location from December 2019 to March 2020. Supplementary Table S3. Airborne fungal diversity molecularly detected in Tianjin outdoor environments, from DNA extracted from isolated strains. Supplementary Table S4. Isolated airborne fungal genera and number of strains. Supplementary Table S5. Isolated air borne fungal species and number of strains [file 12866_2021_2205_MOESM1_ESM.docx]

**Analysis of culturable airborne fungi in outdoor environments in Tianjin, China**

**Supplementary Table S1: Environmental factors recorded in each sampling site at the time of sampling**

| **Month** | **Sampling sites** | **Temperature (**°C**)** | **Wind speed (Km/h)** | **Air pressure (MPa)** | **Humidity (%)** |
| --- | --- | --- | --- | --- | --- |
| December | Nankai G | -1.00 | 3.00 | 1.03 | 64.00 |
|  | Nankai B | 1.00 | 3.00 | 1.03 | 55.00 |
|  | Hebei G | 3.00 | 3.00 | 1.03 | 57.00 |
|  | Hebei B | 2.00 | 3.00 | 1.03 | 67.00 |
|  | Heping G | 2.00 | 0.20 | 1.03 | 53.00 |
|  | Heping B | 1.00 | 1.90 | 1.03 | 53.00 |
|  | Hexi G | -2.00 | 3.00 | 1.03 | 53.00 |
|  | Hexi B | -2.00 | 3.00 | 1.03 | 53.00 |
| January | Nankai G | -2.00 | 3.00 | 1.03 | 28.00 |
|  | Nankai B | 3.00 | 3.00 | 1.03 | 29.00 |
|  | Hebei G | 2.00 | 0.60 | 1.03 | 47.00 |
|  | Hebei B | 2.00 | 0.60 | 1.03 | 42.00 |
|  | Heping G | 1.00 | 3.00 | 1.03 | 40.00 |
|  | Heping B | 2.00 | 3.00 | 1.03 | 29.00 |
|  | Hexi G | -1.00 | 3.00 | 1.03 | 53.00 |
|  | Hexi B | -1.00 | 3.00 | 1.03 | 53.00 |
| February | Nankai G | 3.00 | 18.10 | 1.02 | 54.00 |
|  | Nankai B | 2.00 | 18.50 | 1.02 | 56.00 |
|  | Hebei G | 4.00 | 12.80 | 1.03 | 52.00 |
|  | Hebei B | 5.00 | 12.10 | 1.03 | 51.00 |
|  | Heping G | 5.00 | 17.20 | 1.03 | 52.00 |
|  | Heping B | 4.00 | 17.10 | 1.03 | 50.00 |
|  | Hexi G | 5.00 | 16.40 | 1.03 | 51.00 |
|  | Hexi B | 6.00 | 15.90 | 1.03 | 52.00 |
| March | Nankai G | 24.00 | 11.00 | 1.00 | 28.00 |
|  | Nankai B | 25.00 | 11.00 | 1.05 | 27.00 |
|  | Hebei G | 28.00 | 10.00 | 1.00 | 26.00 |
|  | Hebei B | 27.00 | 11.00 | 1.00 | 26.00 |
|  | Heping G | 28.00 | 9.00 | 1.00 | 23.00 |
|  | Heping B | 27.00 | 9.00 | 1.01 | 26.00 |
|  | Hexi G | 27.00 | 9.00 | 1.01 | 26.00 |
|  | Hexi B | 29.00 | 7.00 | 1.00 | 28.00 |

**In sample site codes, sampling district name (Nankai, Hexi, Heping, Hebei) and site (G = Green, B = Busy) are indicated.**

**Supplementary Table S2: Total number of fungal colonies isolated from each location from December 2019 to March 2020**

| **December** | | | | | | | | | | | | | | | | | |
| --- | --- | --- | --- | --- | --- | --- | --- | --- | --- | --- | --- | --- | --- | --- | --- | --- | --- |
| **Fungal species** | **NanKai P** | | **NanKai G** | | **HePing P** | | **HePing G** | | **HeXi P** | | **HeXi G** | | **HeBei P** | | **HeBei G** | | **Total** |
|  | **SDA** | **MEA** | **SDA** | **MEA** | **SDA** | **MEA** | **SDA** | **MEA** | **SDA** | **MEA** | **SDA** | **MEA** | **SDA** | **MEA** | **SDA** | **MEA** |  |
| *Dothiorella gregaria* | 0 | 0 | 0 | 0 | 0 | 0 | 0 | 0 | 0 | 1 | 0 | 0 | 0 | 0 | 0 | 0 | **1** |
| *Filobasidium chernovii* | 0 | 0 | 0 | 0 | 0 | 0 | 0 | 0 | 0 | 1 | 0 | 0 | 0 | 0 | 0 | 0 | **1** |
| *Filobasidium magnum* | 0 | 0 | 0 | 0 | 0 | 0 | 0 | 0 | 0 | 1 | 0 | 0 | 0 | 0 | 0 | 0 | **1** |
| *Ectophoma multirostrata* | 0 | 0 | 0 | 0 | 0 | 0 | 0 | 0 | 0 | 1 | 0 | 0 | 0 | 0 | 0 | 0 | **1** |
| *Aspergillus pseudoglaucus* | 0 | 0 | 0 | 0 | 0 | 0 | 0 | 0 | 0 | 0 | 0 | 1 | 0 | 0 | 0 | 0 | **1** |
| *Phoma sp.* | 0 | 0 | 0 | 0 | 0 | 0 | 0 | 0 | 1 | 0 | 0 | 0 | 0 | 0 | 0 | 0 | **1** |
| *Trichoderma asperellum* | 0 | 0 | 0 | 0 | 0 | 0 | 0 | 0 | 0 | 0 | 1 | 0 | 0 | 0 | 0 | 0 | **1** |
| *Cladosporium anthropophilum* | 0 | 0 | 0 | 0 | 0 | 0 | 0 | 0 | 0 | 0 | 1 | 0 | 0 | 0 | 0 | 0 | **1** |
| *Colletotrichum gloeosporioides* | 0 | 1 | 0 | 0 | 0 | 0 | 0 | 0 | 0 | 0 | 0 | 0 | 0 | 0 | 0 | 0 | **1** |
| *Aspergillus ochraceus* | 0 | 1 | 0 | 0 | 0 | 0 | 0 | 0 | 0 | 0 | 0 | 0 | 0 | 0 | 0 | 0 | **1** |
| *Phoma herbarum* | 0 | 0 | 0 | 1 | 0 | 0 | 0 | 0 | 0 | 0 | 0 | 0 | 0 | 0 | 0 | 0 | **1** |
| *Aspergillus nomius* | 1 | 0 | 0 | 0 | 0 | 0 | 0 | 0 | 0 | 0 | 0 | 0 | 0 | 0 | 0 | 0 | **1** |
| *Cladosporium asperulatum* | 0 | 0 | 0 | 0 | 0 | 0 | 0 | 0 | 0 | 0 | 0 | 0 | 0 | 0 | 0 | 1 | **1** |
| *Fusarium chlamydosporum* | 0 | 0 | 0 | 0 | 0 | 0 | 0 | 0 | 0 | 0 | 0 | 0 | 0 | 0 | 0 | 1 | **1** |
| *Fusarium equiseti* | 0 | 0 | 0 | 0 | 0 | 0 | 0 | 0 | 0 | 0 | 0 | 0 | 1 | 0 | 0 | 0 | **1** |
| *Cladosporium subuliforme* | 0 | 0 | 0 | 0 | 0 | 0 | 0 | 0 | 0 | 0 | 0 | 0 | 0 | 0 | 1 | 0 | **1** |
| *Phoma multirostrata* | 0 | 0 | 0 | 0 | 0 | 0 | 0 | 0 | 0 | 0 | 0 | 0 | 0 | 0 | 1 | 0 | **1** |
| *Alternaria eichhorniae* | 0 | 0 | 0 | 0 | 0 | 0 | 0 | 0 | 0 | 0 | 0 | 0 | 0 | 0 | 1 | 0 | **1** |
| *Naganishia globosa* | 0 | 0 | 0 | 0 | 0 | 1 | 0 | 0 | 0 | 0 | 0 | 0 | 0 | 0 | 0 | 0 | **1** |
| *Erythrobasidium hasegawianum* | 0 | 0 | 0 | 0 | 0 | 1 | 0 | 0 | 0 | 0 | 0 | 0 | 0 | 0 | 0 | 0 | **1** |
| *Purpureocillium lilacinum* | 0 | 0 | 0 | 0 | 0 | 0 | 0 | 1 | 0 | 0 | 0 | 0 | 0 | 0 | 0 | 0 | **1** |
| *Cladosporium xanthochromaticum* | 0 | 0 | 0 | 0 | 0 | 0 | 0 | 0 | 0 | 0 | 1 | 0 | 0 | 0 | 0 | 1 | **2** |
| *Didymella pedeiae* | 0 | 0 | 0 | 0 | 1 | 0 | 0 | 0 | 0 | 0 | 0 | 0 | 0 | 1 | 0 | 0 | **2** |
| *Didymella subherbarum* | 0 | 0 | 0 | 0 | 0 | 1 | 0 | 0 | 0 | 0 | 0 | 0 | 0 | 1 | 0 | 0 | **2** |
| *Epicoccum latusicollum* | 0 | 0 | 0 | 0 | 0 | 0 | 0 | 0 | 0 | 0 | 0 | 0 | 0 | 1 | 0 | 1 | **2** |
| *Phoma medicaginis* | 0 | 0 | 0 | 0 | 0 | 0 | 0 | 0 | 0 | 0 | 0 | 0 | 1 | 1 | 0 | 0 | **2** |
| *Cladosporium perangustum* | 0 | 0 | 0 | 0 | 0 | 0 | 0 | 0 | 0 | 0 | 0 | 0 | 1 | 0 | 0 | 1 | **2** |
| *Fusarium cf. incarnatum-equiseti* | 0 | 0 | 0 | 0 | 0 | 0 | 0 | 0 | 0 | 0 | 0 | 0 | 1 | 1 | 0 | 0 | **2** |
| *Cladosporium sp.* | 0 | 0 | 0 | 0 | 0 | 0 | 0 | 0 | 0 | 0 | 0 | 0 | 0 | 1 | 1 | 0 | **2** |
| *Sistotrema brinkmannii* | 0 | 0 | 0 | 0 | 0 | 0 | 0 | 0 | 0 | 3 | 1 | 0 | 0 | 0 | 0 | 0 | **4** |
| *Cladosporium pseudocladosporioides* | 0 | 0 | 0 | 0 | 0 | 0 | 0 | 0 | 0 | 1 | 0 | 0 | 0 | 0 | 2 | 1 | **4** |
| *Epicoccum sorghinum* | 0 | 0 | 0 | 0 | 0 | 0 | 0 | 0 | 0 | 0 | 0 | 0 | 0 | 2 | 3 | 0 | **5** |
| *Valsa sordida* | 0 | 0 | 0 | 0 | 0 | 2 | 1 | 1 | 0 | 0 | 0 | 0 | 0 | 1 | 0 | 0 | **5** |
| *Alternaria alternata* | 0 | 0 | 0 | 0 | 2 | 1 | 1 | 0 | 0 | 0 | 0 | 0 | 0 | 1 | 2 | 0 | **7** |
| *Alternaria tenuissima* | 0 | 0 | 0 | 0 | 0 | 0 | 0 | 0 | 0 | 0 | 3 | 0 | 0 | 0 | 2 | 3 | **8** |
| *Cladosporium cladosporioides* | 0 | 1 | 1 | 1 | 3 | 0 | 0 | 0 | 1 | 0 | 0 | 0 | 0 | 2 | 0 | 1 | **10** |
| **Total** | **1** | **3** | **1** | **2** | **6** | **6** | **2** | **2** | **2** | **8** | **7** | **1** | **4** | **12** | **13** | **10** | **80** |
| **January** | | | | | | | | | | | | | | | | | |
| **Fungal species** | **NanKai P** | | **NanKai G** | | **HePing P** | | **HePing G** | | **HeXi P** | | **HeXi G** | | **HeBei P** | | **HeBei G** | | **Total** |
|  | **SDA** | **MEA** | **SDA** | **MEA** | **SDA** | **MEA** | **SDA** | **MEA** | **SDA** | **MEA** | **SDA** | **MEA** | **SDA** | **MEA** | **SDA** | **MEA** |  |
| *Cladosporium cladosporioides* | 0 | 0 | 0 | 0 | 1 | 0 | 0 | 1 | 0 | 1 | 2 | 1 | 2 | 1 | 4 | 0 | **13** |
| *Naganishia albida* | 0 | 0 | 0 | 0 | 0 | 0 | 0 | 0 | 0 | 0 | 0 | 0 | 0 | 0 | 0 | 0 | **1** |
| *Aspergillus ostianus* | 0 | 0 | 0 | 0 | 0 | 0 | 0 | 0 | 0 | 0 | 1 | 2 | 0 | 0 | 0 | 0 | **3** |
| *Curvularia tsudae* | 0 | 0 | 0 | 0 | 0 | 0 | 0 | 0 | 0 | 0 | 0 | 1 | 0 | 0 | 0 | 0 | **1** |
| *Epicoccum nigrum* | 0 | 0 | 0 | 0 | 0 | 0 | 0 | 0 | 0 | 0 | 0 | 1 | 0 | 0 | 0 | 0 | **1** |
| *Fusarium equiseti* | 0 | 0 | 0 | 0 | 0 | 0 | 0 | 0 | 0 | 0 | 2 | 1 | 0 | 0 | 0 | 0 | **3** |
| *Filobasidium magnum* | 0 | 0 | 0 | 0 | 0 | 0 | 0 | 0 | 1 | 0 | 0 | 1 | 0 | 0 | 0 | 0 | **2** |
| *Cladosporium asperulatum* | 0 | 0 | 0 | 0 | 0 | 0 | 0 | 0 | 0 | 0 | 0 | 1 | 0 | 0 | 0 | 0 | **1** |
| *Microsphaeropsis olivacea* | 0 | 0 | 0 | 0 | 0 | 0 | 0 | 0 | 0 | 0 | 0 | 1 | 0 | 0 | 0 | 1 | **2** |
| *Filobasidium uniguttulatum* | 0 | 0 | 0 | 0 | 0 | 0 | 0 | 0 | 1 | 0 | 0 | 0 | 0 | 0 | 0 | 0 | **1** |
| *Alternaria alternata* | 0 | 1 | 0 | 1 | 0 | 0 | 0 | 1 | 0 | 0 | 2 | 0 | 2 | 1 | 1 | 0 | **9** |
| *Nothophoma quercina* | 0 | 0 | 0 | 0 | 0 | 0 | 0 | 0 | 0 | 0 | 1 | 0 | 0 | 0 | 0 | 0 | **1** |
| *Aspergillus nomius* | 0 | 1 | 0 | 0 | 0 | 0 | 0 | 0 | 0 | 0 | 0 | 0 | 0 | 0 | 0 | 0 | **1** |
| *Fungal endophyte sp.* | 0 | 0 | 0 | 1 | 0 | 0 | 0 | 0 | 0 | 0 | 0 | 0 | 0 | 0 | 0 | 0 | **1** |
| *Alternaria brassicicola* | 0 | 0 | 0 | 1 | 0 | 0 | 0 | 0 | 0 | 0 | 0 | 0 | 0 | 0 | 0 | 0 | **1** |
| *Didymella pedeiae* | 0 | 0 | 1 | 0 | 0 | 0 | 0 | 0 | 0 | 0 | 0 | 0 | 0 | 0 | 0 | 0 | **1** |
| *Epicoccum sorghinum* | 0 | 0 | 1 | 0 | 0 | 0 | 0 | 0 | 0 | 0 | 0 | 0 | 0 | 0 | 0 | 0 | **1** |
| *Rhizopus oryzae* | 0 | 0 | 0 | 0 | 0 | 0 | 0 | 0 | 0 | 0 | 0 | 0 | 0 | 2 | 0 | 0 | **2** |
| *Cladosporium sp.* | 0 | 0 | 0 | 0 | 0 | 0 | 0 | 0 | 0 | 0 | 0 | 0 | 0 | 1 | 0 | 0 | **1** |
| *Phoma macrostoma* | 0 | 0 | 0 | 0 | 0 | 0 | 0 | 0 | 0 | 0 | 0 | 0 | 0 | 1 | 0 | 0 | **1** |
| *Massarina igniaria* | 0 | 0 | 0 | 0 | 0 | 0 | 0 | 0 | 0 | 0 | 0 | 0 | 0 | 1 | 0 | 0 | **1** |
| *Aspergillus flavus* | 0 | 0 | 0 | 0 | 0 | 0 | 0 | 0 | 0 | 0 | 0 | 0 | 0 | 1 | 0 | 0 | **1** |
| *Cladosporium xanthochromaticum* | 0 | 0 | 0 | 0 | 0 | 1 | 0 | 0 | 0 | 0 | 0 | 0 | 0 | 1 | 0 | 0 | **2** |
| *Paraconiothyrium hawaiiense* | 0 | 0 | 0 | 0 | 0 | 0 | 0 | 0 | 0 | 0 | 0 | 0 | 0 | 1 | 0 | 0 | **1** |
| *Dothiorella viticola* | 0 | 0 | 0 | 0 | 0 | 0 | 0 | 0 | 0 | 0 | 0 | 0 | 0 | 1 | 0 | 0 | **1** |
| *Naganishia globosa* | 0 | 0 | 0 | 0 | 0 | 1 | 0 | 0 | 0 | 0 | 0 | 0 | 0 | 1 | 0 | 1 | **3** |
| *Phoma multirostrata* | 0 | 0 | 0 | 0 | 0 | 0 | 0 | 0 | 0 | 0 | 0 | 0 | 0 | 0 | 1 | 1 | **2** |
| *Valsa sordida* | 0 | 0 | 0 | 0 | 0 | 0 | 1 | 1 | 0 | 0 | 0 | 0 | 3 | 0 | 0 | 2 | **7** |
| *Cladosporium subuliforme* | 0 | 0 | 0 | 0 | 0 | 0 | 0 | 0 | 0 | 0 | 0 | 0 | 0 | 0 | 0 | 1 | **1** |
| *Ectophoma multirostrata* | 0 | 0 | 0 | 0 | 0 | 0 | 0 | 0 | 0 | 0 | 0 | 0 | 0 | 0 | 2 | 0 | **2** |
| *Didymella sp.* | 0 | 0 | 0 | 0 | 1 | 1 | 0 | 0 | 0 | 0 | 0 | 0 | 0 | 0 | 0 | 0 | **2** |
| *Coniothyrium aleuritis* | 0 | 0 | 0 | 0 | 1 | 0 | 0 | 0 | 0 | 0 | 0 | 0 | 0 | 0 | 0 | 0 | **1** |
| *Alternaria tenuissima* | 0 | 0 | 0 | 0 | 1 | 0 | 1 | 0 | 0 | 0 | 0 | 0 | 0 | 0 | 0 | 0 | **2** |
| *Aureobasidium pullulans* | 0 | 0 | 0 | 0 | 0 | 0 | 1 | 0 | 0 | 0 | 0 | 0 | 0 | 0 | 0 | 0 | **1** |
| **Total** | **0** | **2** | **2** | **3** | **4** | **3** | **3** | **3** | **2** | **2** | **8** | **9** | **7** | **12** | **8** | **6** | **74** |
| **February** | | | | | | | | | | | | | | | | | |
| **Fungal species** | **NanKai P** | | **NanKai G** | | **HePing P** | | **HePing G** | | **HeXi P** | | **HeXi G** | | **HeBei P** | | **HeBei G** | | **Total** |
|  | **SDA** | **MEA** | **SDA** | **MEA** | **SDA** | **MEA** | **SDA** | **MEA** | **SDA** | **MEA** | **SDA** | **MEA** | **SDA** | **MEA** | **SDA** | **MEA** |  |
| *Epicoccum sorghinum* | 0 | 0 | 1 | 0 | 0 | 0 | 0 | 0 | 0 | 0 | 0 | 0 | 0 | 0 | 0 | 0 | **1** |
| *Alternaria alternata* | 2 | 2 | 5 | 1 | 1 | 1 | 4 | 0 | 0 | 1 | 5 | 2 | 0 | 0 | 0 | 1 | **25** |
| *Alternaria longipes* | 0 | 0 | 0 | 1 | 0 | 0 | 0 | 0 | 0 | 0 | 0 | 0 | 0 | 0 | 0 | 0 | **1** |
| *Alternaria tenuissima* | 0 | 1 | 0 | 2 | 2 | 3 | 2 | 0 | 1 | 1 | 2 | 5 | 1 | 2 | 0 | 0 | **22** |
| *Aspergillus flavus* | 0 | 0 | 1 | 0 | 0 | 0 | 0 | 0 | 0 | 0 | 0 | 0 | 0 | 0 | 0 | 0 | **1** |
| *Aspergillus niger* | 0 | 0 | 0 | 0 | 0 | 0 | 0 | 0 | 0 | 0 | 0 | 0 | 0 | 0 | 1 | 0 | **1** |
| *Aureobasidium pullulans* | 0 | 0 | 0 | 0 | 1 | 1 | 0 | 0 | 0 | 0 | 0 | 0 | 0 | 0 | 0 | 0 | **2** |
| *Bipolaris zeae* | 0 | 0 | 0 | 0 | 0 | 0 | 0 | 0 | 0 | 0 | 0 | 0 | 0 | 0 | 1 | 0 | **1** |
| *Cladosporium anthropophilum* | 0 | 0 | 1 | 0 | 0 | 0 | 0 | 2 | 0 | 0 | 0 | 0 | 0 | 0 | 0 | 0 | **3** |
| *Cladosporium asperulatum* | 0 | 0 | 0 | 0 | 0 | 0 | 0 | 0 | 0 | 0 | 0 | 0 | 1 | 0 | 0 | 0 | **1** |
| *Cladosporium cladosporioides* | 1 | 1 | 4 | 1 | 0 | 2 | 5 | 2 | 2 | 1 | 2 | 1 | 3 | 0 | 1 | 3 | **29** |
| *Cladosporium perangustum* | 0 | 0 | 0 | 0 | 0 | 0 | 0 | 0 | 1 | 0 | 0 | 1 | 0 | 0 | 0 | 0 | **2** |
| *Cladosporium sp.* | 0 | 0 | 0 | 0 | 0 | 0 | 0 | 0 | 0 | 0 | 0 | 0 | 0 | 1 | 0 | 0 | **1** |
| *Cladosporium tenuissimum* | 0 | 0 | 0 | 1 | 0 | 0 | 0 | 0 | 1 | 0 | 2 | 0 | 0 | 0 | 0 | 0 | **4** |
| *Cladosporium uredinicola* | 0 | 1 | 0 | 0 | 0 | 0 | 0 | 0 | 0 | 0 | 0 | 0 | 0 | 0 | 0 | 0 | **1** |
| *Colletotrichum gloeosporioides* | 3 | 0 | 0 | 0 | 0 | 0 | 0 | 0 | 0 | 0 | 0 | 1 | 0 | 0 | 0 | 0 | **4** |
| *Coreomyces sp.* | 0 | 0 | 0 | 0 | 0 | 0 | 0 | 0 | 0 | 0 | 1 | 0 | 0 | 0 | 0 | 0 | **1** |
| *Curvularia sp.* | 1 | 0 | 0 | 0 | 0 | 0 | 0 | 0 | 0 | 0 | 0 | 0 | 0 | 0 | 0 | 0 | **1** |
| *Daldinia eschscholtzii* | 0 | 0 | 0 | 0 | 0 | 0 | 0 | 0 | 0 | 0 | 1 | 0 | 0 | 0 | 0 | 0 | **1** |
| [Didymella glomerata](#alnHdr_1508351557) | 0 | 0 | 0 | 0 | 0 | 1 | 0 | 0 | 0 | 0 | 0 | 1 | 0 | 0 | 0 | 0 | **2** |
| *Didymella macrostoma* | 1 | 0 | 0 | 0 | 0 | 0 | 0 | 0 | 0 | 0 | 1 | 0 | 0 | 0 | 0 | 0 | **2** |
| *Didymella pedeiae* | 2 | 1 | 0 | 0 | 0 | 0 | 0 | 0 | 0 | 0 | 1 | 0 | 0 | 0 | 2 | 0 | **6** |
| *Erythrobasidium hasegawianum* | 0 | 0 | 0 | 0 | 0 | 1 | 0 | 0 | 0 | 0 | 0 | 1 | 0 | 0 | 0 | 0 | **2** |
| *Fusarium oxysporum* | 1 | 0 | 0 | 0 | 0 | 0 | 0 | 0 | 0 | 0 | 0 | 0 | 0 | 0 | 0 | 0 | **1** |
| *Fusarium verticillioides* | 0 | 0 | 0 | 0 | 1 | 2 | 0 | 1 | 0 | 3 | 1 | 3 | 1 | 2 | 1 | 0 | **15** |
| *Kazachstania humilis* | 0 | 0 | 0 | 0 | 0 | 0 | 0 | 0 | 0 | 0 | 0 | 0 | 0 | 0 | 1 | 0 | **1** |
| *Lasiodiplodia theobromae* | 0 | 0 | 0 | 0 | 0 | 0 | 0 | 0 | 0 | 0 | 0 | 0 | 0 | 0 | 1 | 0 | **1** |
| *Microsphaeropsis olivacea* | 0 | 0 | 0 | 0 | 0 | 0 | 0 | 0 | 0 | 0 | 1 | 0 | 0 | 0 | 0 | 0 | **1** |
| *Naganishia albida* | 1 | 2 | 2 | 8 | 2 | 1 | 0 | 0 | 0 | 0 | 0 | 1 | 0 | 0 | 0 | 0 | **17** |
| *Papiliotrema flavescens* | 0 | 0 | 0 | 0 | 0 | 0 | 0 | 2 | 0 | 1 | 1 | 2 | 0 | 1 | 0 | 1 | **8** |
| *Paraconiothyrium hawaiiense* | 0 | 0 | 0 | 0 | 0 | 1 | 0 | 0 | 0 | 0 | 0 | 0 | 0 | 0 | 0 | 0 | **1** |
| *Periconia epilithographicola* | 0 | 0 | 0 | 0 | 0 | 0 | 0 | 0 | 0 | 0 | 0 | 1 | 0 | 0 | 0 | 0 | **1** |
| *Phoma multirostrata* | 0 | 0 | 1 | 0 | 0 | 0 | 0 | 0 | 0 | 0 | 1 | 0 | 0 | 0 | 0 | 0 | **2** |
| *Symmetrospora foliicola* | 0 | 0 | 0 | 0 | 0 | 0 | 0 | 0 | 0 | 0 | 0 | 0 | 0 | 0 | 0 | 1 | **1** |
| *Symmetrospora symmetrica* | 0 | 0 | 0 | 0 | 0 | 0 | 0 | 0 | 0 | 0 | 0 | 0 | 0 | 1 | 0 | 0 | **1** |
| *Cladosporium cladosporioides* | 0 | 0 | 0 | 0 | 0 | 0 | 0 | 0 | 0 | 0 | 1 | 0 | 0 | 0 | 0 | 0 | **1** |
| **Total** | **12** | **8** | **15** | **14** | **7** | **13** | **11** | **7** | **5** | **7** | **20** | **19** | **6** | **7** | **8** | **6** | **165** |
| **March** | | | | | | | | | | | | | | | | | |
| ***Fungal species*** | **NanKai P** | | **NanKai G** | | **HePing P** | | **HePing G** | | **HeXi P** | | **HeXi G** | | **HeBei P** | | **HeBei G** | | **Total** |
|  | **SDA** | **MEA** | **SDA** | **MEA** | **SDA** | **MEA** | **SDA** | **MEA** | **SDA** | **MEA** | **SDA** | **MEA** | **SDA** | **MEA** | **SDA** | **MEA** |  |
| [*Acrodontium crateriforme*](#alnHdr_1616362720) | 0 | 0 | 0 | 0 | 0 | 0 | 0 | 0 | 0 | 0 | 0 | 1 | 0 | 0 | 0 | 0 | **1** |
| [*Allophoma labilis*](#alnHdr_908376432) | 0 | 0 | 0 | 0 | 0 | 0 | 0 | 0 | 0 | 0 | 0 | 0 | 0 | 0 | 1 | 0 | **1** |
| *Alternaria alternata* | 9 | 1 | 1 | 3 | 0 | 4 | 0 | 0 | 1 | 2 | 1 | 0 | 1 | 2 | 4 | 5 | **34** |
| [*Alternaria arborescens*](#alnHdr_1836447795) | 0 | 0 | 0 | 0 | 0 | 0 | 0 | 0 | 0 | 1 | 0 | 0 | 0 | 0 | 0 | 0 | **1** |
| [*Alternaria porri*](#alnHdr_1353266127) | 0 | 0 | 0 | 0 | 0 | 0 | 0 | 0 | 0 | 0 | 0 | 0 | 0 | 0 | 1 | 0 | **1** |
| [*Alternaria sp.*](#alnHdr_1591647502) | 0 | 0 | 0 | 0 | 0 | 0 | 0 | 0 | 0 | 0 | 0 | 0 | 0 | 0 | 1 | 0 | **1** |
| [*Alternaria tenuissima*](#alnHdr_1241824226) | 0 | 1 | 0 | 0 | 0 | 0 | 1 | 2 | 0 | 0 | 0 | 0 | 1 | 1 | 0 | 4 | **10** |
| [*Aplosporella javeedii*](#alnHdr_1631814841) | 6 | 0 | 0 | 0 | 0 | 0 | 0 | 0 | 0 | 0 | 0 | 0 | 0 | 0 | 0 | 0 | **6** |
| [*Aureobasidium namibiae*](#alnHdr_1612852056) | 0 | 0 | 0 | 0 | 0 | 0 | 0 | 0 | 0 | 0 | 0 | 0 | 1 | 1 | 0 | 0 | **2** |
| *Aureobasidium proteae* | 0 | 0 | 0 | 0 | 0 | 0 | 0 | 0 | 0 | 0 | 0 | 0 | 0 | 1 | 1 | 0 | **3** |
| *Aureobasidium pullulans* | 1 | 2 | 0 | 0 | 0 | 1 | 0 | 0 | 0 | 0 | 0 | 0 | 1 | 0 | 0 | 1 | **5** |
| [*Aureobasidium sp.*](#alnHdr_1612852146) | 0 | 0 | 0 | 0 | 1 | 0 | 0 | 0 | 0 | 0 | 0 | 0 | 0 | 0 | 0 | 0 | **1** |
| *Candida auris* | 0 | 0 | 0 | 0 | 0 | 0 | 0 | 0 | 0 | 0 | 0 | 0 | 0 | 0 | 0 | 1 | **1** |
| [*Cladosporium anthropophilum*](#alnHdr_1787112178) | 0 | 0 | 0 | 1 | 0 | 2 | 0 | 0 | 0 | 0 | 0 | 1 | 0 | 2 | 0 | 0 | **6** |
| *Cladosporium cladosporioides* | 0 | 0 | 0 | 0 | 0 | 0 | 0 | 0 | 0 | 0 | 0 | 0 | 1 | 0 | 0 | 0 | **1** |
| [*Cladosporium gossypiicola*](#alnHdr_1657837982) | 0 | 0 | 0 | 0 | 0 | 1 | 0 | 0 | 0 | 0 | 0 | 0 | 0 | 0 | 0 | 0 | **1** |
| [*Cladosporium herbarum*](#alnHdr_1644884796) | 0 | 0 | 0 | 0 | 0 | 1 | 0 | 1 | 0 | 0 | 0 | 0 | 0 | 4 | 0 | 0 | **6** |
| [*Cladosporium ramotenellum*](#alnHdr_1839250434) | 0 | 0 | 0 | 0 | 0 | 0 | 0 | 0 | 0 | 0 | 0 | 0 | 0 | 0 | 1 | 0 | **1** |
| [*Cladosporium sp.*](#alnHdr_1802567472) | 0 | 0 | 0 | 0 | 2 | 0 | 0 | 0 | 0 | 0 | 0 | 0 | 0 | 0 | 0 | 1 | **3** |
| [*Cladosporium tenuissimum*](#alnHdr_1441342080) | 0 | 0 | 0 | 0 | 0 | 0 | 0 | 0 | 0 | 0 | 0 | 0 | 0 | 0 | 0 | 1 | **1** |
| [*Colletotrichum gloeosporioides*](#alnHdr_321172561) | 0 | 0 | 0 | 0 | 0 | 0 | 0 | 0 | 0 | 0 | 0 | 0 | 1 | 0 | 0 | 0 | **1** |
| [*Coniothyrium aleuritis*](#alnHdr_1493564064) | 0 | 0 | 0 | 1 | 0 | 0 | 0 | 0 | 0 | 0 | 0 | 0 | 0 | 0 | 0 | 0 | **1** |
| [*Coniothyrium pyrinum*](#alnHdr_1817191496) | 1 | 0 | 0 | 0 | 0 | 0 | 0 | 0 | 0 | 0 | 0 | 0 | 0 | 0 | 0 | 0 | **1** |
| *Cytospora chrysosperma* | 0 | 0 | 0 | 0 | 0 | 0 | 0 | 0 | 0 | 0 | 0 | 0 | 1 | 0 | 0 | 0 | **1** |
| [*Didymella bryoniae*](#alnHdr_154721261) | 1 | 0 | 0 | 0 | 0 | 0 | 0 | 0 | 0 | 0 | 0 | 0 | 0 | 0 | 0 | 0 | **1** |
| *Didymella macrostoma* | 0 | 1 | 0 | 0 | 0 | 0 | 0 | 0 | 0 | 0 | 0 | 0 | 0 | 0 | 0 | 0 | **1** |
| [*Didymella pedeiae*](#alnHdr_1477502079) | 1 | 4 | 0 | 0 | 0 | 0 | 0 | 0 | 0 | 0 | 0 | 0 | 0 | 0 | 0 | 1 | **6** |
| [*Didymellaceae sp.*](#alnHdr_1840379226) | 1 | 1 | 0 | 0 | 0 | 0 | 0 | 0 | 0 | 0 | 0 | 0 | 0 | 0 | 0 | 0 | **2** |
| *Dioszegia zsoltii* | 0 | 0 | 0 | 0 | 1 | 0 | 0 | 0 | 0 | 0 | 0 | 0 | 0 | 0 | 1 | 0 | **2** |
| [*Dothideomycetes sp.*](#alnHdr_1401690725) | 0 | 0 | 0 | 0 | 0 | 0 | 0 | 1 | 1 | 0 | 0 | 0 | 0 | 0 | 0 | 0 | **2** |
| [*Dothiorella gregaria*](#alnHdr_189162046) | 0 | 0 | 0 | 0 | 0 | 1 | 0 | 0 | 0 | 0 | 0 | 0 | 1 | 0 | 0 | 0 | **2** |
| [*Dothiorella viticola*](#alnHdr_1276314859) | 5 | 1 | 0 | 0 | 0 | 0 | 0 | 0 | 0 | 0 | 0 | 0 | 0 | 0 | 0 | 0 | **6** |
| *Epicoccum nigrum* | 0 | 0 | 0 | 0 | 0 | 4 | 0 | 0 | 0 | 0 | 0 | 0 | 2 | 1 | 0 | 0 | **7** |
| *Fusarium equiseti* | 0 | 0 | 0 | 0 | 0 | 2 | 2 | 0 | 0 | 0 | 0 | 1 | 1 | 0 | 0 | 3 | **9** |
| [*Fusarium tricinctum*](#alnHdr_1820378428) | 0 | 0 | 0 | 1 | 0 | 0 | 0 | 0 | 0 | 0 | 0 | 0 | 0 | 0 | 0 | 0 | **1** |
| [*Herpotrichia striatispora*](#alnHdr_1580977281) | 0 | 0 | 0 | 0 | 1 | 0 | 0 | 0 | 0 | 0 | 0 | 0 | 0 | 0 | 0 | 0 | **1** |
| *Kazachstania humilis* | 1 | 0 | 0 | 0 | 0 | 0 | 0 | 0 | 0 | 0 | 0 | 0 | 0 | 0 | 0 | 0 | **1** |
| [*Microsphaeropsis olivacea*](#alnHdr_1797100830) | 1 | 0 | 0 | 0 | 0 | 1 | 0 | 0 | 0 | 0 | 0 | 0 | 2 | 1 | 0 | 0 | **5** |
| *Naganishia adeliensis* | 0 | 0 | 0 | 0 | 1 | 0 | 0 | 0 | 0 | 0 | 0 | 0 | 0 | 0 | 0 | 0 | **1** |
| *Naganishia albida* | 4 | 2 | 2 | 0 | 1 | 1 | 0 | 1 | 0 | 0 | 0 | 2 | 4 | 0 | 0 | 1 | **18** |
| [*Naganishia friedmannii*](#alnHdr_1612852082) | 0 | 0 | 0 | 0 | 0 | 0 | 0 | 0 | 0 | 0 | 0 | 0 | 1 | 0 | 0 | 0 | **1** |
| *Naganishia globosa* | 6 | 15 | 0 | 0 | 0 | 0 | 0 | 0 | 0 | 1 | 0 | 1 | 4 | 3 | 0 | 0 | **30** |
| *Naganishia liquefaciens* | 1 | 1 | 0 | 0 | 0 | 0 | 0 | 0 | 0 | 0 | 0 | 0 | 0 | 0 | 0 | 0 | **2** |
| [*Naganishia randhawae*](#alnHdr_1612852046) | 0 | 0 | 0 | 0 | 0 | 0 | 0 | 0 | 0 | 0 | 0 | 1 | 0 | 0 | 0 | 0 | **1** |
| [*Naganishia uzbekistanensis*](#alnHdr_1316032186) | 0 | 0 | 0 | 0 | 0 | 1 | 1 | 0 | 0 | 0 | 0 | 1 | 0 | 0 | 0 | 0 | **3** |
| [*Neosetophoma samarorum*](#alnHdr_1526409248) | 0 | 1 | 0 | 0 | 0 | 0 | 0 | 0 | 0 | 0 | 0 | 0 | 0 | 0 | 0 | 0 | **1** |
| [*Nothophoma sp.*](#alnHdr_1778599777) | 0 | 0 | 0 | 0 | 1 | 1 | 1 | 0 | 0 | 0 | 0 | 0 | 1 | 0 | 0 | 0 | **4** |
| [*Papiliotrema laurentii*](#alnHdr_1531397805) | 0 | 0 | 0 | 0 | 0 | 0 | 0 | 0 | 0 | 0 | 0 | 0 | 2 | 0 | 0 | 0 | **2** |
| *Paraconiothyrium archidendri* | 0 | 0 | 0 | 0 | 0 | 0 | 0 | 0 | 0 | 0 | 0 | 0 | 0 | 0 | 1 | 0 | **1** |
| [*Paraconiothyrium hawaiiense*](#alnHdr_254028327) | 2 | 3 | 0 | 0 | 0 | 0 | 0 | 0 | 0 | 0 | 0 | 1 | 0 | 0 | 1 | 1 | **8** |
| [*Phaeosphaeria sp.*](#alnHdr_317383339) | 1 | 0 | 0 | 0 | 0 | 0 | 0 | 0 | 0 | 0 | 0 | 0 | 0 | 0 | 0 | 0 | **1** |
| [*Phoma herbarum*](#alnHdr_667673579) | 0 | 0 | 0 | 0 | 0 | 0 | 0 | 0 | 0 | 0 | 0 | 0 | 0 | 0 | 0 | 2 | **2** |
| [*Phoma macrostoma var. macrostoma*](#alnHdr_526482363) | 0 | 2 | 0 | 0 | 0 | 0 | 0 | 0 | 0 | 0 | 0 | 0 | 2 | 0 | 0 | 0 | **4** |
| *Phoma medicaginis* | 0 | 1 | 0 | 0 | 1 | 0 | 0 | 0 | 0 | 0 | 0 | 0 | 1 | 0 | 0 | 0 | **3** |
| *Phoma sojicola* | 0 | 0 | 0 | 0 | 0 | 0 | 0 | 0 | 0 | 0 | 0 | 0 | 1 | 0 | 0 | 0 | **1** |
| [*Phoma sp.*](#alnHdr_1121724666) | 0 | 2 | 0 | 0 | 0 | 0 | 0 | 0 | 1 | 0 | 0 | 0 | 0 | 0 | 0 | 0 | **3** |
| [*Rhodotorula mucilaginosa*](#alnHdr_1797857408) | 0 | 0 | 0 | 0 | 0 | 0 | 0 | 0 | 0 | 0 | 0 | 1 | 0 | 1 | 1 | 1 | **4** |
| [*Stagonosporopsis cucurbitacearum*](#alnHdr_1829368272) | 0 | 0 | 0 | 0 | 0 | 0 | 0 | 0 | 0 | 0 | 0 | 0 | 1 | 0 | 0 | 0 | **1** |
| [*Talaromyces funiculosus*](#alnHdr_1419375410) | 0 | 0 | 0 | 0 | 0 | 0 | 0 | 0 | 2 | 0 | 0 | 0 | 1 | 0 | 1 | 0 | **4** |
| *Talaromyces marneffei* | 0 | 0 | 0 | 0 | 0 | 0 | 0 | 0 | 0 | 0 | 0 | 1 | 0 | 1 | 3 | 1 | **6** |
| *Thelebolus microsporus* | 0 | 0 | 0 | 0 | 0 | 0 | 0 | 0 | 0 | 0 | 0 | 0 | 0 | 0 | 0 | 0 | **1** |
| [*Valsa sordida*](#alnHdr_1674985841) | 0 | 0 | 0 | 0 | 1 | 0 | 0 | 0 | 0 | 0 | 0 | 0 | 0 | 0 | 0 | 0 | **1** |
| *Vishniacozyma sp.* | 0 | 0 | 0 | 0 | 0 | 0 | 0 | 1 | 0 | 0 | 0 | 0 | 0 | 0 | 0 | 0 | **1** |
| *Vishniacozyma tephrensis* | 0 | 0 | 0 | 0 | 0 | 0 | 0 | 1 | 0 | 0 | 0 | 0 | 0 | 0 | 0 | 0 | **1** |
| **Total** | **41** | **38** | **3** | **6** | **10** | **20** | **5** | **7** | **5** | **4** | **1** | **11** | **31** | **19** | **17** | **23** | **241** |

**Supplementary Table S3: Airborne fungal diversity molecularly detected in Tianjin outdoor enviroments, from DNA extracted from isolated strains**

| **Sample** | **Sampling** | **Sample** | **GenBank** | **Best BLAST** | **Accession** | **Overlap** | **%** | **Max** | **Query** | **E** |
| --- | --- | --- | --- | --- | --- | --- | --- | --- | --- | --- |
| **No.** | **Month** | **Code** |  | **Match(es)** | **Code** | **Length** | **Match** | **Score** | **Cover** | **Value** |
| 1 | DEC | HEXI B MEA | MW723617 | *Dothiorella gregaria* | EU520055.1 | 866 | 99.77% | 866 | 99% | 0 |
| 2 | DEC | HEXI B MEA | MW723618 | Filobasidium sp. | MN299307.1 | 946 | 99.81% | 946 | 100% | 0 |
|  |  |  |  | *Filobasidium chernovii* | MN848516.1 | 935 | 99.42% | 935 | 100% | 0 |
| 3 | DEC | HEXI B MEA | MW723619 | *Sistotrema brinkmannii* | KM232472.1 | 1417 | 98.51% | 1417 | 100% | 0 |
| 4 | DEC | HEXI B MEA | MW723620 | *Filobasidium magnum* | MH203407.1 | 955 | 100.00% | 955 | 100% | 0 |
| 5 | DEC | HEXI B MEA | MW723621 | Sistotrema sp. | MN905876.1 | 973 | 100.00% | 973 | 98% | 0 |
|  |  |  |  | *Sistotrema brinkmannii* | KM232472.1 | 973 | 100.00% | 973 | 98% | 0 |
| 6 | DEC | HEXI B MEA | MW723622 | *Sistotrema brinkmannii* | KM232472.1 | 1367 | 97.39% | 1367 | 100% | 0 |
| 7 | DEC | HEXI B MEA | MW723623 | *Ectophoma multirostrata* | MG840682.1 | 1666 | 96.95% | 1208 | 99% | 0 |
| 8 | DEC | HEXI B MEA | MW723624 | *Cladosporium pseudocladosporioides* | MT582794.1 | 854 | 100.00% | 854 | 100% | 0 |
| 9 | DEC | HEXI G MEA | MW723625 | *Aspergillus pseudoglaucus* | MT582752.1 | 922 | 100.00% | 922 | 100% | 0 |
| 10 | DEC | HEXI B SDA | MW723626 | *Cladosporium cladosporioides* | MT573472.1 | 957 | 97.17% | 957 | 100% | 0 |
| 11 | DEC | HEXI B SDA | MW723627 | Phoma sp. | KY088083.1 | 575 | 100.00% | 575 | 100% | 5.00E-160 |
| 12 | DEC | HEXI G SDA | MW723628 | *Trichoderma asperellum* | KC312632.1 | 1648 | 98.40% | 1648 | 99% | 0 |
| 13 | DEC | HEXI G SDA | MW723629 | *Sistotrema brinkmannii* | KM232477.1 | 2922 | 98.43% | 2922 | 100% | 0 |
| 14 | DEC | HEXI G SDA | MW723630 | *Cladosporium anthropophilum* | MT508803.1 | 408 | 100.00% | 408 | 100% | 4.00E-110 |
| 15 | DEC | HEXI G SDA | MW723631 | Cladosporium sp. | KC871037.1 | 983 | 99.80% | 983 | 99% | 0 |
|  |  |  |  | *Cladosporium cladosporioides* | KT959291.1 | 979 | 100.00% | 979 | 98% | 0 |
| 16 | DEC | HEXI G SDA | MW723632 | *Cladosporium cladosporioides* | KT959291.1 | 846 | 97.39% | 846 | 100% | 0 |
| 17 | DEC | HEXI G SDA | MW723633 | *Cladosporium cladosporioides* | KT959291.1 | 808 | 95.99% | 808 | 100% | 0 |
| 18 | DEC | HEXI G SDA | MW723634 | *Cladosporium xanthochromaticum* | MH605338.1 | 944 | 98.16% | 944 | 100% | 0 |
| 19 | DEC | NANKAI B MEA | MW723635 | *Colletotrichum gloeosporioides* | HQ874970.1 | 58 | 100.00% | 58 | 100% | 8.00E-06 |
| 20 | DEC | NANKAI B MEA | MW723636 | *Cladosporium cladosporioides* | MT573472.1 | 931 | 96.46% | 931 | 99% | 0 |
| 21 | DEC | NANKAI B MEA | MW723637 | *Aspergillus ochraceus* | MN088855.1 | 953 | 95.62% | 953 | 100% | 0 |
| 22 | DEC | NANKAI G MEA | MW723638 | *Phoma herbarum* | KJ767079.1 | 883 | 96.29% | 883 | 100% | 0 |
| 23 | DEC | NANKAI G MEA | MW723639 | *Cladosporium cladosporioides* | MT573472.1 | 944 | 96.81% | 944 | 99% | 0 |
| 24 | DEC | NANKAI B SDA | MW723640 | *Aspergillus nomius* | MF510822.1 | 1679 | 98.71% | 1243 | 100% | 0 |
| 25 | DEC | NANKAI G SDA | MW723641 | *Cladosporium cladosporioides* | JQ768323.1 | 963 | 97.20% | 963 | 99% | 0 |
| 26 | DEC | HEBEI B MEA | MW723642 | *Epicoccum sorghinum* | MN215621.1 | 1197 | 97.70% | 1197 | 100% | 0 |
| 27 | DEC | HEBEI B MEA | MW723643 | *Didymella pedeiae* | MH923258.1 | 1533 | 97.25% | 1110 | 99% | 0 |
| 28 | DEC | HEBEI B MEA | MW723644 | *Didymella subherbarum* | KR534651.1 | 1507 | 96.26% | 1050 | 100% | 0 |
| 29 | DEC | HEBEI B MEA | MW723645 | *Cladosporium cladosporioides* | JQ768323.1 | 977 | 97.56% | 977 | 100% | 0 |
| 30 | DEC | HEBEI B MEA | MW723646 | *Epicoccum sorghinum* | MN215621.1 | 1186 | 97.41% | 1186 | 99% | 0 |
| 31 | DEC | HEBEI B MEA | MW723647 | *Epicoccum latusicollum* | MN215613.1 | 1149 | 96.94% | 1149 | 100% | 0 |
| 32 | DEC | HEBEI B MEA | MW723648 | *Phoma medicaginis* | KT192426.1 | 854 | 97.97% | 854 | 99% | 0 |
| 33 | DEC | HEBEI G MEA | MW723649 | *Epicoccum latusicollum* | MN215613.1 | 1144 | 96.79% | 1144 | 100% | 0 |
| 34 | DEC | HEBEI G MEA | MW723650 | *Cladosporium asperulatum* | MN202774.1 | 909 | 97.73% | 909 | 99% | 0 |
| 35 | DEC | HEBEI G MEA | MW723651 | *Cladosporium cladosporioides* | MT258647.1 | 972 | 97.21% | 972 | 100% | 0 |
| 36 | DEC | HEBEI G MEA | MW723652 | *Alternaria tenuissima* | KU937315.1 | 2046 | 98.06% | 1524 | 99% | 0 |
| 37 | DEC | HEBEI G MEA | MW723653 | *Cladosporium pseudocladosporioides* | MT582794.1 | 1476 | 98.34% | 1476 | 100% | 0 |
| 38 | DEC | HEBEI G MEA | MW723654 | *Alternaria tenuissima* | MN907695.1 | 1003 | 97.93% | 1003 | 100% | 0 |
| 39 | DEC | HEBEI G MEA | MW723655 | *Cladosporium perangustum* | KF706664.1 | 893 | 97.87% | 893 | 99% | 0 |
| 40 | DEC | HEBEI G MEA | MW723656 | *Cladosporium xanthochromaticum* | MT464453.1 | 905 | 98.26% | 905 | 100% | 0 |
| 41 | DEC | HEBEI G MEA | MW723657 | *Alternaria tenuissima* | MN907695.1 | 972 | 97.21% | 972 | 100% | 0 |
| 42 | DEC | HEBEI G MEA | MW723658 | *Fusarium chlamydosporum* | MT032393.1 | 963 | 98.02% | 963 | 99% | 0 |
| 43 | DEC | HEBEI B SDA | MW723659 | *Fusarium equiseti* | KJ412501.1 | 918 | 96.73% | 918 | 99% | 0 |
| 44 | DEC | HEBEI B SDA | MW723660 | *Fusarium incarnatum* | MN646258.1 | 915 | 96.74% | 915 | 99% | 0 |
| 45 | DEC | HEBEI B SDA | MW723661 | *Cladosporium perangustum* | MF473173.1 | 937 | 96.63% | 937 | 99% | 0 |
| 46 | DEC | HEBEI B SDA | MW723662 | *Phoma medicaginis* | KT192426.1 | 915 | 97.06% | 915 | 99% | 0 |
| 47 | DEC | HEBEI G SDA | MW723663 | *Cladosporium subuliforme* | KP701938.1 | 1389 | 97.31% | 1389 | 99% | 0 |
| 48 | DEC | HEBEI G SDA | MW723664 | *Alternaria tenuissima* | MN907695.1 | 922 | 95.65% | 922 | 100% | 0 |
| 49 | DEC | HEBEI G SDA | MW723665 | *Alternaria alternata* | KT192411.1 | 952 | 96.53% | 952 | 99% | 0 |
| 50 | DEC | HEBEI G SDA | MW723666 | *Epicoccum sorghinum* | MT125854.1 | 1194 | 97.70% | 1194 | 99% | 0 |
| 51 | DEC | HEBEI G SDA | MW723667 | *Cladosporium pseudocladosporioides* | MT582794.1 | 1402 | 96.51% | 1402 | 99% | 0 |
| 52 | DEC | HEBEI G SDA | MW723668 | *Phoma multirostrata* | JX966636.1 | 1678 | 96.67% | 1197 | 100% | 0 |
| 53 | DEC | HEBEI G SDA | MW723669 | *Cladosporium pseudocladosporioides* | MT582794.1 | 1448 | 98.08% | 1448 | 99% | 0 |
| 54 | DEC | HEBEI G SDA | MW723670 | *Epicoccum sorghinum* | MK516206.1 | 1170 | 97.11% | 1170 | 99% | 0 |
| 55 | DEC | HEBEI G SDA | MW723671 | *Epicoccum sorghinum* | MT125854.1 | 1114 | 95.68% | 1114 | 99% | 0 |
| 56 | DEC | HEBEI G SDA | MW723672 | Cladosporium sp. | MK336600.1 | 953 | 96.54% | 953 | 99% | 0 |
|  |  |  |  | *Cladosporium xanthochromaticum* | MF473319.1 | 953 | 96.54% | 953 | 99% | 0 |
| 57 | DEC | HEBEI G SDA | MW723673 | *Alternaria tenuissima* | MN907695.1 | 944 | 96.35% | 944 | 99% | 0 |
| 58 | DEC | HEBEI G SDA | MW723674 | *Alternaria alternata* | KT192411.1 | 939 | 96.17% | 939 | 100% | 0 |
| 59 | DEC | HEBEI G SDA | MW723675 | *Alternaria eichhorniae* | MN128537.1 | 902 | 95.10% | 902 | 100% | 0 |
| 60 | DEC | HEBEI B MEA | MW723676 | *Alternaria alternata* | KJ728679.1 | 2313 | 96.61% | 1271 | 99% | 0 |
| 61 | DEC | HEBEI B MEA | MW723677 | *Cladosporium cladosporioides* | MK127535.1 | 846 | 95.98% | 846 | 98% | 0 |
| 62 | DEC | HEBEI B MEA | MW723678 | *Fusarium incarnatum* | MN646258.1 | 915 | 96.74% | 915 | 99% | 0 |
| 63 | DEC | HEBEI B MEA | MW723679 | Cladosporium sp. | JQ388271.1 | 926 | 96.76% | 926 | 99% | 0 |
|  |  |  |  | *Cladosporium cladosporioides* | MK127535.1 | 854 | 96.18% | 854 | 93% | 0 |
| 64 | DEC | HEBEI B MEA | MW723680 | *Valsa sordida* | MK994101.1 | 1144 | 100.00% | 1144 | 100% | 0 |
| 65 | DEC | HEPING B MEA | MW723681 | *Didymella subherbarum* | KR534651.1 | 1427 | 95.45% | 1013 | 98% | 0 |
| 66 | DEC | HEPING B MEA | MW723682 | Alternaria sp. | KP027305.1 | 1828 | 97.90% | 1144 | 100% | 0 |
|  |  |  |  | *Alternaria alternata* | JQ080319.1 | 1967 | 97.43% | 1116 | 100% | 0 |
| 67 | DEC | HEPING B MEA | MW723683 | *Valsa sordida* | MK994101.1 | 987 | 95.24% | 987 | 100% | 0 |
| 68 | DEC | HEPING B MEA | MW723684 | Uncultured fungus | MT635325.1 | 1044 | 96.69% | 1044 | 98% | 0 |
|  |  |  |  | *Naganishia globosa* | MH809978.1 | 1044 | 96.69% | 1044 | 98% | 0 |
| 69 | DEC | HEPING B MEA | MW723685 | *Valsa sordida* | MK994101.1 | 1033 | 96.65% | 1033 | 99% | 0 |
| 70 | DEC | HEPING B MEA | MW723686 | *Erythrobasidium hasegawianum* | AF444522.1 | 977 | 95.18% | 977 | 100% | 0 |
| 71 | DEC | HEPING G MEA | MW723687 | *Valsa sordida* | MK994101.1 | 985 | 99.81% | 985 | 100% | 0 |
| 72 | DEC | HEPING G MEA | MW723688 | *Purpureocillium lilacinum* | MF996811.1 | 996 | 100.00% | 996 | 100% | 0 |
| 73 | DEC | HEPING B SDA | MW723689 | *Alternaria alternata* | MN402464.1 | 889 | 100.00% | 889 | 100% | 0 |
| 74 | DEC | HEPING B SDA | MW723690 | *Alternaria alternata* | MN615420.1 | 867 | 100.00% | 867 | 100% | 0 |
| 75 | DEC | HEPING B SDA | MW723691 | *Didymella pedeiae* | MH923258.1 | 1450 | 95.22% | 1048 | 99% | 0 |
| 76 | DEC | HEPING B SDA | MW723692 | *Cladosporium cladosporioides* | KU527802.2 | 756 | 97.80% | 756 | 99% | 0 |
| 77 | DEC | HEPING B SDA | MW723693 | *Cladosporium cladosporioides* | MT508787.1 | 893 | 98.42% | 893 | 100% | 0 |
| 78 | DEC | HEPING B SDA | MW723694 | Cladosporium sp. | HQ671188.1 | 841 | 95.65% | 841 | 99% | 0 |
|  |  |  |  | *Cladosporium cladosporioides* | KY114882.1 | 835 | 95.63% | 835 | 99% | 0 |
| 79 | DEC | HEPING G SDA | MW723695 | Alternaria sp. | KP027305.1 | 2503 | 96.28% | 1393 | 100% | 0 |
|  |  |  |  | *Alternaria alternata* | JQ080319.1 | 2621 | 95.69% | 1356 | 100% | 0 |
| 80 | DEC | HEPING G SDA | MW723696 | *Valsa sordida* | MK656251.1 | 911 | 98.46% | 911 | 100% | 0 |
| 1 | JAN | HEXI B MEA | MW723697 | *Cladosporium cladosporioides* | MT258647.1 | 989 | 97.74% | 989 | 100% | 0 |
| 2 | JAN | HEXI B MEA | MW723698 | *Naganishia albida* | KY238156.1 | 1120 | 100.00% | 1120 | 100% | 0 |
| 3 | JAN | HEXI G MEA | MW723699 | Cladosporium sp. | KT588451.1 | 908 | 100.00% | 908 | 99% | 0 |
|  |  |  |  | *Cladosporium cladosporioides* | LT603044.1 | 908 | 100.00% | 908 | 99% | 0 |
| 4 | JAN | HEXI G MEA | MW723700 | *Aspergillus ostianus* | MT446137.1 | 998 | 97.92% | 998 | 100% | 0 |
| 5 | JAN | HEXI G SDA | MW723701 | *Aspergillus ostianus* | MT446137.1 | 965 | 96.88% | 965 | 100% | 0 |
| 6 | JAN | HEXI G MEA | MW723702 | *Curvularia tsudae* | MN368920.1 | 936 | 100.00% | 936 | 100% | 0 |
| 7 | JAN | HEXI G MEA | MW723703 | *Epicoccum nigrum* | KY587324.1 | 1069 | 99.76% | 803 | 100% | 0 |
| 8 | JAN | HEXI G MEA | MW723704 | *Fusarium equiseti* | MT626672.1 | 944 | 97.81% | 944 | 99% | 0 |
| 9 | JAN | HEXI G MEA | MW723705 | *Filobasidium magnum* | MF114305.1 | 1008 | 100.00% | 967 | 88% | 0 |
| 10 | JAN | HEXI G MEA | MW723706 | *Aspergillus ostianus* | MT446137.1 | 994 | 97.91% | 994 | 99% | 0 |
| 11 | JAN | HEXI G MEA | MW723707 | *Cladosporium asperulatum* | MN202774.1 | 922 | 100.00% | 922 | 99% | 0 |
| 12 | JAN | HEXI G MEA | MW723707 | *Microsphaeropsis olivacea* | MN944412.1 | 813 | 99.76% | 813 | 100% | 0 |
| 13 | JAN | HEXI B SDA | MW723708 | *Filobasidium uniguttulatum* | KC152903.1 | 965 | 100.00% | 965 | 99% | 0 |
| 14 | JAN | HEXI B SDA | MW723709 | *Filobasidium magnum* | MF114305.1 | 991 | 100.00% | 991 | 99% | 0 |
| 15 | JAN | HEXI G SDA | MW723710 | *Cladosporium cladosporioides* | MT573472.1 | 924 | 96.11% | 924 | 100% | 0 |
| 16 | JAN | HEXI G SDA | MW723711 | *Fusarium equiseti* | MT626672.1 | 941 | 97.63% | 941 | 99% | 0 |
| 17 | JAN | HEXI G SDA | MW723712 | *Fusarium equiseti* | MT626672.1 | 926 | 97.10% | 926 | 100% | 0 |
| 18 | JAN | HEXI G SDA | MW723713 | *Cladosporium cladosporioides* | MT258647.1 | 976 | 97.24% | 976 | 99% | 0 |
| 19 | JAN | HEXI G SDA | MW723714 | *Alternaria alternata* | MT453271.1 | 2004 | 97.53% | 2004 | 100% | 0 |
| 20 | JAN | HEXI G SDA | MW723715 | *Alternaria alternata* | MT453271.1 | 2030 | 97.95% | 2030 | 100% | 0 |
| 21 | JAN | HEXI G SDA | MW723716 | *Nothophoma quercina* | MN267493.1 | 926 | 100.00% | 926 | 100% | 0 |
| 22 | JAN | NANKAI B MEA | MW723717 | *Alternaria alternata* | MF422130.1 | 1285 | 97.77% | 1081 | 100% | 0 |
| 23 | JAN | NANKAI B MEA | MW723718 | *Aspergillus nomius* | KR905619.1 | 1576 | 98.66% | 1576 | 99% | 0 |
| 24 | JAN | NANKAI G MEA | MW723719 | Fungal endophyte | HM537037.1 | 918 | 96.26% | 918 | 100% | 0 |
|  |  |  |  | *Cladosporium ramotenellum* | MT529231.1 | 913 | 96.08% | 913 | 100% | 0 |
| 25 | JAN | NANKAI G MEA | MW723720 | *Alternaria alternata* | MF422133.1 | 1003 | 96.10% | 1003 | 100% | 0 |
| 26 | JAN | NANKAI G MEA | MW723721 | Alternaria sp. | KJ527009.1 | 1079 | 99.33% | 1079 | 100% | 0 |
|  |  |  |  | *Alternaria brassicicola* | KF542557.1 | 1079 | 99.33% | 1079 | 100% | 0 |
| 27 | JAN | NANKAI G SDA | MW723722 | *Didymella pedeiae* | MH923258.1 | 1496 | 96.36% | 1085 | 100% | 0 |
| 28 | JAN | NANKAIG SDA | MW723723 | *Epicoccum sorghinum* | MF061768.1 | 856 | 97.06% | 856 | 100% | 0 |
| 29 | JAN | HEBEI B MEA | MW723724 | *Rhizopus oryzae* | MH877020.1 | 1131 | 96.89% | 1131 | 99% | 0 |
| 30 | JAN | HEBEI B MEA | MW723725 | *Alternaria alternata* | MN907695.1 | 957 | 96.70% | 957 | 99% | 0 |
| 31 | JAN | HEBEI B MEA | MW723726 | Cladosporium sp. | MK336600.1 | 937 | 95.42% | 937 | 99% | 0 |
| 32 | JAN | HEBEI B MEA | MW723727 | *Phoma macrostoma* | KF293857.1 | 822 | 96.23% | 822 | 99% | 0 |
| 33 | JAN | HEBEI B MEA | MW723728 | *Massarina igniaria* | GQ377480.1 | 950 | 97.49% | 950 | 99% | 0 |
| 34 | JAN | HEBEI B MEA | MW723729 | *Aspergillus flavus* | MT541875.1 | 966 | 98.03% | 966 | 98% | 0 |
| 35 | JAN | HEBEI B MEA | MW723730 | *Cladosporium xanthochromaticum* | MF473319.1 | 944 | 96.21% | 944 | 99% | 0 |
| 36 | JAN | HEBEI B MEA | MW723731 | *Rhizopus oryzae* | MH877018.1 | 1591 | 96.58% | 1591 | 99% | 0 |
| 37 | JAN | HEBEI B MEA | MW723732 | *Paraconiothyrium hawaiiense* | EU715661.1 | 1838 | 95.46% | 1838 | 99% | 0 |
| 38 | JAN | HEBEI B MEA | MW723733 | *Dothiorella viticola* | KY385661.1 | 885 | 95.37% | 885 | 99% | 0 |
| 39 | JAN | HEBEI B MEA | MW723734 | *Naganishia globosa* | MH809978.1 | 1343 | 95.20% | 1343 | 99% | 0 |
| 40 | JAN | HEBEI B MEA | MW723735 | *Cladosporium cladosporioides* | MT258647.1 | 989 | 97.74% | 989 | 98% | 0 |
| 41 | JAN | HEBEI G MEA | MW723736 | *Microsphaeropsis olivacea* | MN944412.1 | 909 | 96.58% | 909 | 97% | 0 |
| 42 | JAN | HEBEI G MEA | MW723737 | *Phoma multirostrata* | JX966636.1 | 1610 | 95.74% | 1164 | 100% | 0 |
| 43 | JAN | HEBEI G MEA | MW723738 | *Valsa sordida* | MK994101.1 | 1000 | 95.57% | 1000 | 100% | 0 |
| 44 | JAN | HEBEI G MEA | MW723739 | *Valsa sordida* | MK994101.1 | 979 | 95.08% | 979 | 100% | 0 |
| 45 | JAN | HEBEI G MEA | MW723740 | *Cladosporium subuliforme* | KP701938.1 | 1339 | 96.34% | 1339 | 99% | 0 |
| 46 | JAN | HEBEI G MEA | MW723741 | *Naganishia globosa* | MH809978.1 | 1491 | 99.15% | 1491 | 100% | 0 |
| 47 | JAN | HEBEI B SDA | MW723742 | *Valsa sordida* | MK994101.1 | 994 | 95.79% | 994 | 98% | 0 |
| 48 | JAN | HEBEI B SDA | MW723743 | *Valsa sordida* | MK994101.1 | 1072 | 98.06% | 1072 | 100% | 0 |
| 49 | JAN | HEBEI B SDA | MW723744 | *Alternaria alternata* | KT192411.1 | 658 | 97.35% | 658 | 99% | 0 |
| 50 | JAN | HEBEI B SDA | MW723745 | *Alternaria alternata* | MT420645.1 | 935 | 96.02% | 935 | 100% | 0 |
| 51 | JAN | HEBEI B SDA | MW723746 | *Cladosporium cladosporioides* | KY114882.1 | 885 | 97.32% | 885 | 99% | 0 |
| 52 | JAN | HEBEI B SDA | MW723748 | *Cladosporium cladosporioides* | MK127535.1 | 883 | 96.96% | 883 | 99% | 0 |
| 53 | JAN | HEBEI B SDA | MW723749 | *Valsa sordida* | MK994101.1 | 1042 | 97.39% | 1042 | 98% | 0 |
| 54 | JAN | HEBEI G SDA | MW723750 | *Ectophoma multirostrata* | MG840682.1 | 1627 | 97.13% | 1173 | 98% | 0 |
| 55 | JAN | HEBEI G SDA | MW723751 | *Phoma multirostrata* | JX966636.1 | 1688 | 97.36% | 1216 | 99% | 0 |
| 56 | JAN | HEBEI G SDA | MW723752 | *Cladosporium cladosporioides* | KY114882.1 | 929 | 98.85% | 929 | 100% | 0 |
| 57 | JAN | HEBEI G SDA | MW723753 | *Cladosporium cladosporioides* | KY114882.1 | 822 | 95.22% | 822 | 100% | 0 |
| 58 | JAN | HEBEI G SDA | MW723754 | Cladosporium sp. | HQ671188.1 | 817 | 95.17% | 817 | 99% | 0 |
|  |  |  |  | *Cladosporium cladosporioides* | KY114882.1 | 813 | 95.16% | 813 | 99% | 0 |
| 59 | JAN | HEBEI G SDA | MW723755 | *Alternaria alternata* | MT646481.1 | 933 | 96.15% | 933 | 99% | 0 |
| 60 | JAN | HEBEI G SDA | MW723756 | *Cladosporium cladosporioides* | KY114882.1 | 821 | 95.04% | 821 | 99% | 0 |
| 61 | JAN | HEBEI G SDA | MW723757 | *Ectophoma multirostrata* | MG840682.1 | 1664 | 97.09% | 1212 | 100% | 0 |
| 62 | JAN | HEPING B MEA | MW723758 | *Cladosporium xanthochromaticum* | MF473319.1 | 981 | 97.72% | 981 | 97% | 0 |
| 63 | JAN | HEPING B MEA | MW723759 | Didymella sp. | MG198901.1 | 2433 | 98.29% | 1428 | 100% | 0 |
| 64 | JAN | HEPING B MEA | MW723760 | *Naganishia globosa* | MH809978.1 | 1426 | 97.07% | 1426 | 100% | 0 |
| 65 | JAN | HEPING G MEA | MW723761 | *Valsa sordida* | MK994101.1 | 961 | 99.81% | 961 | 100% | 0 |
| 66 | JAN | HEPING G MEA | MW723762 | *Alternaria alternata* | MN615420.1 | 867 | 100.00% | 867 | 100% | 0 |
| 67 | JAN | HEPING G MEA | MW723763 | *Cladosporium cladosporioides* | MK127535.1 | 935 | 98.85% | 935 | 99% | 0 |
| 68 | JAN | HEPING B SDA | MW723764 | Didymella sp. | MG198901.1 | 2343 | 96.88% | 1448 | 99% | 0 |
| 69 | JAN | HEPING B SDA | MW723765 | *Coniothyrium aleuritis* | KP749188.1 | 2346 | 96.83% | 1411 | 100% | 0 |
| 70 | JAN | HEPING B SDA | MW723766 | *Alternaria tenuissima* | MN907695.1 | 1024 | 98.62% | 1024 | 99% | 0 |
| 71 | JAN | HEPING B SDA | MW723767 | *Cladosporium cladosporioides* | MK127535.1 | 846 | 95.65% | 846 | 99% | 0 |
| 72 | JAN | HEPING G SDA | MW723768 | *Valsa sordida* | KF293840.1 | 1035 | 100.00% | 1035 | 100% | 0 |
| 73 | JAN | HEPING G SDA | MW723769 | *Aureobasidium pullulans* | KR912253.1 | 1507 | 98.24% | 1090 | 100% | 0 |
| 74 | JAN | HEPING G SDA | MW723770 | *Alternaria tenuissima* | MT497426.1 | 1547 | 98.25% | 1101 | 100% | 0 |
| 1 | FEB | NANKAI G SDA | MW723771 | *Epicoccum sorghinum* | MF061768.1 | 905 | 99.40% | 905 | 98% | 0 |
| 2 | FEB | NANKAI G SDA | MW723772 | Alternaria sp. | MN856342.1 | 970 | 99.62% | 970 | 98% | 0 |
|  |  |  |  | *Alternaria alternata* | MN856355.1 | 968 | 99.62% | 968 | 97% | 0 |
| 3 | FEB | NANKAI G SDA | MW723773 | *Alternaria alternata* | JQ080319.1 | 3209 | 96.14% | 1631 | 96% | 0 |
| 4 | FEB | NANKAI G SDA | MW723774 | *Alternaria alternata* | JQ080319.1 | 3390 | 98.51% | 1773 | 99% | 0 |
| 5 | FEB | NANKAI G SDA | MW723775 | *Alternaria alternata* | KJ728679.1 | 3076 | 99.14% | 1679 | 100% | 0 |
| 6 | FEB | NANKAI G SDA | MW723776 | *Cladosporium cladosporioides* | MH341182.1 | 911 | 98.46% | 911 | 98% | 0 |
| 7 | FEB | NANKAI G MEA | MW723777 | *Cladosporium cladosporioides* | MG731215.1 | 2418 | 98.54% | 2418 | 99% | 0 |
| 8 | FEB | NANKAI B MEA | MW723778 | *Alternaria alternata* | JQ080319.1 | 3098 | 95.25% | 1574 | 98% | 0 |
| 9 | FEB | NANKAI B MEA | MW723779 | Uncultured soil fungus | HM037664.1 | 749 | 96.68% | 749 | 46% | 0 |
|  |  |  |  | *Cladosporium uredinicola* | FJ025160.1 | 743 | 95.31% | 743 | 48% | 0 |
| 10 | FEB | NANKAI B MEA | MW723780 | *Alternaria alternata* | JQ080319.1 | 3491 | 99.40% | 1823 | 100% | 0 |
| 11 | FEB | NANKAI B MEA | MW723781 | *Didymella pedeiae* | MH923258.1 | 1603 | 98.48% | 1158 | 99% | 0 |
| 12 | FEB | NANKAI B SDA | MW723782 | *Didymella pedeiae* | MH923258.1 | 1616 | 98.48% | 1160 | 99% | 0 |
| 13 | FEB | NANKAI B SDA | MW723783 | *Colletotrichum gloeosporioides* | HQ874970.1 | 802 | 98.89% | 802 | 99% | 0 |
| 14 | FEB | NANKAI B SDA | MW723784 | *Alternaria alternata* | JQ080319.1 | 3098 | 95.25% | 1456 | 98% | 0 |
| 15 | FEB | NANKAI G MEA | MW723785 | *Alternaria longipes* | MN589608.1 | 885 | 98.04% | 885 | 99% | 0 |
| 16 | FEB | NANKAI G MEA | MW723786 | *Alternaria alternata* | MN907695.1 | 845 | 98.74% | 845 | 51% | 0 |
| 17 | FEB | NANKAI G MEA | MW723787 | *Alternaria tenuissima* | MF952613.1 | 1638 | 95.83% | 1120 | 71% | 0 |
| 18 | FEB | NANKAI G MEA | MW723788 | *Alternaria tenuissima* | MF952613.1 | 1586 | 95.01% | 1098 | 70% | 0 |
| 19 | FEB | NANKAI B SDA | MW723789 | Curvularia sp. | JN207338.1 | 712 | 98.99% | 712 | 99% | 0 |
|  |  |  |  | *Curvularia tuberculata* | MT229266.1 | 678 | 97.49% | 678 | 99% | 0 |
| 20 | FEB | NANKAI B SDA | MW723790 | *Colletotrichum gloeosporioides* | HQ874970.1 | 784 | 98.22% | 784 | 99% | 0 |
| 21 | FEB | NANKAI B SDA | MW723791 | *Didymella macrostoma* | MN944409.1 | 756 | 100.00% | 756 | 80% | 0 |
| 22 | FEB | NANKAI B SDA | MW723792 | *Alternaria alternata* | JQ080319.1 | 3367 | 98.59% | 1760 | 99% | 0 |
| 23 | FEB | NANKAI B SDA | MW723793 | *Colletotrichum gloeosporioides* | HQ874970.1 | 776 | 97.99% | 776 | 99% | 0 |
| 24 | FEB | NANKAI B SDA | MW723794 | *Naganishia albida* | MG551274.1 | 2838 | 99.28% | 2002 | 99% | 0 |
| 25 | FEB | HEXI G SDA | MW723795 | *Daldinia eschscholtzii* | MK928969.1 | 950 | 98.51% | 950 | 99% | 0 |
| 26 | FEB | HEXI G SDA | MW723796 | *Alternaria alternata* | MH521178.1 | 1044 | 98.64% | 1044 | 99% | 0 |
| 27 | FEB | HEXI G SDA | MW723797 | Coreomyces sp. | MG602602.1 | 1982 | 99.28% | 1982 | 99% | 0 |
| 28 | FEB | HEXI G SDA | MW723798 | *Alternaria alternata* | KJ728679.1 | 1699 | 98.93% | 1002 | 99% | 0 |
| 29 | FEB | HEXI G SDA | MW723799 | *Didymella macrostoma* | MN944409.1 | 623 | 99.42% | 623 | 67% | 3.00E-174 |
| 30 | FEB | HEXI G SDA | MW723800 | *Alternaria alternata* | MH201516.1 | 717 | 97.18% | 717 | 77% | 0 |
| 31 | FEB | HEXI G SDA | MW723801 | *Rhodotorula dairenensis* | MN920648.1 | 939 | 98.86% | 939 | 99% | 0 |
| 32 | FEB | HEXI G SDA | MW723802 | *Alternaria tenuissima* | KU937315.1 | 2138 | 99.20% | 1587 | 100% | 0 |
| 33 | FEB | HEXI G MEA | MW723803 | *Alternaria tenuissima* | MT497426.1 | 1645 | 98.10% | 1092 | 100% | 0 |
| 34 | FEB | HEXI G MEA | MW723804 | *Alternaria tenuissima* | KU937315.1 | 1368 | 95.05% | 972 | 91% | 0 |
| 35 | FEB | HEXI G MEA | MW723805 | *Alternaria tenuissima* | MT497426.1 | 1643 | 99.05% | 1109 | 100% | 0 |
| 36 | FEB | HEXI G MEA | MW723806 | *Alternaria tenuissima* | KU937315.1 | 1828 | 99.29% | 1266 | 100% | 0 |
| 37 | FEB | HEXI G MEA | MW723807 | *Naganishia albida* | MG551287.1 | 1959 | 99.29% | 1266 | 100% | 0 |
| 38 | FEB | HEXI G MEA | MW723808 | *Alternaria alternata* | MH521178.1 | 1051 | 98.98% | 1051 | 99% | 0 |
| 39 | FEB | HEXI B MEA | MW723809 | *Fusarium verticillioides* | MK790042.1 | 1619 | 96.29% | 1086 | 77% | 0 |
| 40 | FEB | HEXI B MEA | MW723810 | *Fusarium verticillioides* | MK790042.1 | 1797 | 99.41% | 1227 | 99% | 0 |
| 41 | FEB | HEXI B MEA | MW723811 | *Alternaria alternata* | MK659949.1 | 1061 | 98.82% | 1061 | 99% | 0 |
| 42 | FEB | HEXI B MEA | MW723812 | *Fusarium verticillioides* | MK790042.1 | 1739 | 98.52% | 1192 | 99% | 0 |
| 43 | FEB | HEXI B MEA | MW723813 | *Alternaria tenuissima* | MF952613.1 | 1499 | 95.92% | 1072 | 66% | 0 |
| 44 | FEB | HEXI G MEA | MW723814 | *Alternaria tenuissima* | MT497426.1 | 1739 | 98.57% | 1216 | 100% | 0 |
| 45 | FEB | HEXI G MEA | MW723815 | *Cladosporium anthropophilum* | MF472926.1 | 1526 | 99.41% | 1526 | 99% | 0 |
| 46 | FEB | HEXI G MEA | MW723816 | *Cladosporium anthropophilum* | MK965098.1 | 979 | 98.73% | 979 | 99% | 0 |
| 47 | FEB | HEXI G MEA | MW723817 | *Colletotrichum gloeosporioides* | HQ874970.1 | 806 | 99.33% | 806 | 99% | 0 |
| 48 | FEB | HEXI G MEA | MW723818 | *Alternaria alternata* | MT556702.1 | 933 | 98.31% | 933 | 99% | 0 |
| 49 | FEB | HEXI G MEA | MW723819 | *Cladosporium perangustum* | KF706664.1 | 898 | 98.25% | 898 | 97% | 0 |
| 50 | FEB | HEPING G SDA | MW723820 | *Alternaria alternata* | JQ676196.1 | 920 | 97.93% | 920 | 99% | 0 |
| 51 | FEB | HEPING G SDA | MW723821 | *Alternaria alternata* | MT000437.1 | 952 | 98.87% | 952 | 100% | 0 |
| 52 | FEB | HEPING G SDA | MW723822 | *Alternaria alternata* | MT000437.1 | 933 | 98.31% | 933 | 99% | 0 |
| 53 | FEB | HEPING G SDA | MW723823 | Cladosporium sp. | MK111512.1 | 894 | 98.43% | 894 | 99% | 0 |
|  |  |  |  | *Cladosporium cladosporioides* | MK111520.1 | 893 | 98.43% | 893 | 99% | 0 |
| 54 | FEB | HEPING G SDA | MW723824 | Cladosporium sp. | MK111548.1 | 902 | 98.82% | 902 | 96% | 0 |
|  |  |  |  | *Cladosporium cladosporioides* | MK111520.1 | 902 | 98.82% | 902 | 96% | 0 |
| 55 | FEB | HEPING G SDA | MW723825 | *Alternaria alternata* | MT000437.1 | 955 | 98.97% | 955 | 99% | 0 |
| 56 | FEB | HEPING G SDA | MW723826 | *Alternaria tenuissima* | MH374277.1 | 1554 | 98.96% | 1554 | 99% | 0 |
| 57 | FEB | HEPING G SDA | MW723827 | Alternaria sp. | MN856392.1 | 793 | 100.00% | 793 | 77% | 0 |
|  |  |  |  | *Alternaria tenuissima* | MN822661.1 | 793 | 100.00% | 793 | 77% | 0 |
| 58 | FEB | HEPING B SDA | MW723828 | *Alternaria tenuissima* | MF952613.1 | 1817 | 96.43% | 1153 | 86% | 0 |
| 59 | FEB | HEPING B SDA | MW723829 | *Alternaria tenuissima* | MF952613.1 | 1734 | 98.71% | 1214 | 99% | 0 |
| 60 | FEB | HEPING B SDA | MW723830 | *Fusarium verticillioides* | MK790042.1 | 1706 | 98.34% | 1166 | 97% | 0 |
| 61 | FEB | HEPING B MEA | MW723831 | *Alternaria alternata* | MK907718.1 | 931 | 98.13% | 931 | 99% | 0 |
| 62 | FEB | HEPING B MEA | MW723832 | *Alternaria tenuissima* | KU937315.1 | 2103 | 98.18% | 1530 | 99% | 0 |
| 63 | FEB | HEPING B MEA | MW723833 | *Cladosporium cladosporioides* | MK111520.1 | 887 | 98.04% | 887 | 99% | 0 |
| 64 | FEB | HEPING B MEA | MW723834 | *Didymella glomerata* | MG664756.1 | 1101 | 98.87% | 1101 | 99% | 0 |
| 65 | FEB | HEPING B MEA | MW723835 | *Papiliotrema flavescens* | MH931267.1 | 1334 | 98.17% | 1334 | 99% | 0 |
| 66 | FEB | HEPING B MEA | MW723836 | *Alternaria tenuissima* | MT497426.1 | 1728 | 98.43% | 1210 | 100% | 0 |
| 67 | FEB | HEPING B MEA | MW723837 | *Alternaria tenuissima* | MF952613.1 | 1701 | 96.24% | 1129 | 86% | 0 |
| 68 | FEB | HEBEI B SDA | MW723838 | *Cladosporium cladosporioides* | MG731215.1 | 2442 | 99.04% | 2442 | 99% | 0 |
| 69 | FEB | HEBEI B SDA | MW723839 | *Fusarium verticillioides* | MK790042.1 | 1636 | 96.59% | 1098 | 77% | 0 |
| 70 | FEB | HEXI G SDA | MW723840 | *Cladosporium cladosporioides* | MG731215.1 | 1367 | 98.70% | 1367 | 100% | 0 |
| 71 | FEB | NANKAI G SDA | MW723841 | *Phoma multirostrata* | JX966636.1 | 1715 | 98.57% | 1232 | 99% | 0 |
| 72 | FEB | NANKAI G SDA | MW723842 | *Cladosporium cladosporioides* | KC329631.1 | 935 | 99.42% | 935 | 98% | 0 |
| 73 | FEB | HEBEI B MEA | MW723843 | Cladosporium sp. | KY621330.1 | 2626 | 98.66% | 2111 | 99% | 0 |
|  |  |  |  | *Cladosporium cladosporioides* | MG731215.1 | 1394 | 95.27% | 1029 | 69% | 0 |
| 74 | FEB | HEBEI B MEA | MW723844 | *Alternaria tenuissima* | MF952613.1 | 1749 | 98.43% | 1208 | 99% | 0 |
| 75 | FEB | HEBEI G MEA | MW723845 | *Papiliotrema flavescens* | MH931267.1 | 1349 | 98.44% | 1349 | 99% | 0 |
| 76 | FEB | HEBEI B SDA | MW723846 | *Alternaria tenuissima* | KU937315.1 | 2114 | 98.40% | 1541 | 99% | 0 |
| 77 | FEB | HEBEI G MEA | MW723847 | Alternaria sp. | MT000599.1 | 933 | 99.23% | 933 | 96% | 0 |
|  |  |  |  | *Alternaria alternata* | MT000437.1 | 933 | 99.23% | 933 | 96% | 0 |
| 78 | FEB | HEBEI B MEA | MW723848 | *Alternaria tenuissima* | MH374277.1 | 1565 | 99.31% | 1565 | 99% | 0 |
| 79 | FEB | HEBEI G SDA | MW723849 | *Fusarium verticillioides* | MK790042.1 | 1610 | 95.99% | 1075 | 77% | 0 |
| 80 | FEB | HEBEI B SDA | MW723850 | *Cladosporium asperulatum* | MN202774.1 | 900 | 100.00% | 900 | 100% | 0 |
| 81 | FEB | HEPING G SDA | MW723851 | *Cladosporium cladosporioides* | MK111520.1 | 874 | 97.65% | 874 | 99% | 0 |
| 82 | FEB | HEPING G SDA | MW723852 | *Cladosporium cladosporioides* | MF077224.1 | 955 | 98.00% | 955 | 99% | 0 |
| 83 | FEB | HEPING G MEA | MW723853 | *Fusarium verticillioides* | KY495190.1 | 2043 | 98.78% | 2043 | 99% | 0 |
| 84 | FEB | HEPING G SDA | MW723854 | *Cladosporium cladosporioides* | KX664320.1 | 1947 | 98.72% | 1947 | 99% | 0 |
| 85 | FEB | HEBEI B SDA | MW723855 | *Cladosporium cladosporioides* | KC329631.1 | 867 | 97.09% | 867 | 97% | 0 |
| 86 | FEB | NANKAI B MEA | MW723856 | *Alternaria tenuissima* | MN907695.1 | 1005 | 98.26% | 1005 | 99% | 0 |
| 87 | FEB | HEPING G MEA | MW723857 | *Cladosporium cladosporioides* | MF072677.1 | 887 | 97.67% | 887 | 99% | 0 |
| 88 | FEB | HEBEI G SDA | MW723858 | *Cladosporium cladosporioides* | MG976288.1 | 857 | 97.42% | 857 | 99% | 0 |
| 89 | FEB | HEPING G MEA | MW723859 | Uncultured fungus | MT236874.1 | 2809 | 95.55% | 1410 | 85% | 0 |
|  |  |  |  | *Cladosporium cladosporioides* | MG731215.1 | 1629 | 95.92% | 987 | 84% | 0 |
| 90 | FEB | HEXI G SDA | MW723860 | *Ectophoma multirostrata* | MG840682.1 | 1839 | 100.00% | 1327 | 100% | 0 |
| 91 | FEB | HEXI G MEA | MW723861 | *Fusarium verticillioides* | MK790049.1 | 1819 | 98.56% | 1227 | 99% | 0 |
| 92 | FEB | NANKAI G SDA | MW723862 | *Aspergillus flavus* | MH447084.1 | 928 | 96.76% | 928 | 99% | 0 |
| 93 | FEB | HEBEI G MEA | MW723863 | *Cladosporium cladosporioides* | KC329631.1 | 846 | 96.48% | 846 | 99% | 0 |
| 94 | FEB | HEXI G SDA | MW723864 | *Alternaria alternata* | MF422133.1 | 1079 | 98.37% | 1079 | 100% | 0 |
| 95 | FEB | HEXI G SDA | MW723865 | Cladosporium sp. | MN275871.1 | 865 | 97.25% | 865 | 99% | 0 |
|  |  |  |  | *Cladosporium cladosporioides* | MF475952.1 | 865 | 97.25% | 865 | 99% | 0 |
| 96 | FEB | HEXI B MEA | MW723866 | *Papiliotrema flavescens* | MH023208.1 | 867 | 97.81% | 867 | 99% | 0 |
| 97 | FEB | HEXI B MEA | MW723867 | *Cladosporium cladosporioides* | MF475952.1 | 996 | 98.58% | 996 | 98% | 0 |
| 98 | FEB | HEXI G SDA | MW723868 | *Cladosporium cladosporioides* | MN704703.1 | 885 | 97.49% | 885 | 99% | 0 |
| 99 | FEB | NANKAI B MEA | MW723869 | *Cladosporium cladosporioides* | MH884119.1 | 837 | 96.45% | 837 | 99% | 0 |
| 100 | FEB | HEPING B SDA | MW723870 | *Alternaria alternata* | MF422130.1 | 1226 | 99.13% | 1026 | 100% | 0 |
| 101 | FEB | HEXI G MEA | MW723871 | *Fusarium verticillioides* | MK790042.1 | 1734 | 98.52% | 1192 | 99% | 0 |
| 102 | FEB | HEXI G MEA | MW723872 | *Didymella glomerata* | KT827261.1 | 828 | 98.93% | 828 | 99% | 0 |
| 103 | FEB | HEXI G MEA | MW723873 | *Erythrobasidium hasegawianum* | AF444522.1 | 1068 | 98.20% | 1068 | 99% | 0 |
| 104 | FEB | NANKAI B SDA | MW723874 | *Cladosporium cladosporioides* | MF687304.1 | 922 | 97.58% | 922 | 98% | 0 |
| 105 | FEB | HEXI B MEA | MW723875 | *Fusarium verticillioides* | MK790049.1 | 1821 | 98.26% | 1208 | 99% | 0 |
| 106 | FEB | HEXI G SDA | MW723876 | *Fusarium verticillioides* | MK790051.1 | 1588 | 98.15% | 1131 | 99% | 0 |
| 107 | FEB | HEXI G SDA | MW723877 | *Cladosporium tenuissimum* | MK183813.1 | 905 | 97.90% | 905 | 100% | 0 |
| 108 | FEB | HEPING B MEA | MW723878 | *Fusarium verticillioides* | MK790042.1 | 1614 | 96.75% | 1127 | 99% | 0 |
| 109 | FEB | HEPING B MEA | MW723879 | *Fusarium verticillioides* | MK790049.1 | 1780 | 98.41% | 1216 | 99% | 0 |
| 110 | FEB | HEXI G SDA | MW723880 | *Didymella pedeiae* | MH923258.1 | 1671 | 100.00% | 1216 | 100% | 0 |
| 111 | FEB | HEXI G SDA | MW723881 | *Cladosporium tenuissimum* | MK183813.1 | 854 | 96.17% | 854 | 99% | 0 |
| 112 | FEB | HEXI G SDA | MW723882 | *Papiliotrema flavescens* | MH931267.1 | 1338 | 98.30% | 1338 | 98% | 0 |
| 113 | FEB | HEBEI G MEA | MW723883 | *Cladosporium cladosporioides* | MH341182.1 | 880 | 97.12% | 880 | 99% | 0 |
| 114 | FEB | HEXI B SDA | MW723884 | *Cladosporium cladosporioides* | MH341182.1 | 891 | 97.50% | 891 | 99% | 0 |
| 115 | FEB | HEBEI G SDA | MW723885 | *Cladosporium cladosporioides* | MH341182.1 | 926 | 98.66% | 926 | 99% | 0 |
| 116 | FEB | HEBEI G SDA | MW723886 | *Kazachstania humilis* | MN913450.1 | 1092 | 98.86% | 1092 | 99% | 0 |
| 117 | FEB | HEBEI B SDA | MW723887 | *Cladosporium cladosporioides* | KR084328.1 | 848 | 96.67% | 848 | 99% | 0 |
| 118 | FEB | HEXI B SDA | MW723888 | *Cladosporium tenuissimum* | MT072081.1 | 941 | 99.61% | 941 | 97% | 0 |
| 119 | FEB | HEXI G MEA | MW723889 | *Fusarium verticillioides* | MK790051.1 | 1357 | 98.61% | 1018 | 98% | 0 |
| 120 | FEB | HEBEI G SDA | MW723890 | *Lasiodiplodia theobromae* | JX282407.1 | 1395 | 96.77% | 1395 | 100% | 0 |
| 121 | FEB | HEBEI G SDA | MW723891 | *Didymella pedeiae* | MH923258.1 | 1634 | 99.09% | 1179 | 99% | 0 |
| 122 | FEB | HEBEI G SDA | MW723892 | *Bipolaris zeae* | MT138920.1 | 658 | 100.00% | 658 | 100% | 0 |
| 123 | FEB | NANKAI B SDA | MW723893 | *Fusarium oxysporum* | MN709614.1 | 1896 | 98.79% | 1325 | 99% | 0 |
| 124 | FEB | NANKAI G SDA | MW723894 | *Cladosporium anthropophilum* | MK111487.1 | 922 | 99.03% | 922 | 97% | 0 |
| 125 | FEB | NANKAI G MEA | MW723895 | *Naganishia albida* | KY238183.1 | 1050 | 98.33% | 1050 | 99% | 0 |
| 126 | FEB | NANKAI G MEA | MW723896 | *Naganishia albida* | KY238185.1 | 1062 | 98.66% | 1062 | 99% | 0 |
| 127 | FEB | NANKAI G MEA | MW723897 | *Naganishia albida* | KY238183.1 | 1044 | 98.32% | 1044 | 99% | 0 |
| 128 | FEB | HEPING B SDA | MW723898 | *Naganishia albida* | KY238185.1 | 1075 | 99.33% | 1075 | 98% | 0 |
| 129 | FEB | HEPING B MEA | MW723899 | *Naganishia albida* | KY238185.1 | 1075 | 99.49% | 1075 | 98% | 0 |
| 130 | FEB | HEPING G MEA | MW723900 | *Papiliotrema flavescens* | MH931267.1 | 1375 | 99.08% | 1375 | 99% | 0 |
| 131 | FEB | NANKAI G MEA | MW723901 | *Naganishia albida* | KY238170.1 | 1070 | 99.32% | 1070 | 98% | 0 |
| 132 | FEB | NANKAI G MEA | MW723902 | *Naganishia albida* | KY238170.1 | 1051 | 98.33% | 1051 | 99% | 0 |
| 133 | FEB | NANKAI B MEA | MW723903 | *Naganishia albida* | KY238161.1 | 1044 | 98.32% | 1044 | 99% | 0 |
| 134 | FEB | NANKAI B MEA | MW723904 | *Naganishia albida* | KY238157.1 | 1064 | 98.99% | 1064 | 99% | 0 |
| 135 | FEB | HEBEI B MEA | MW723905 | *Symmetrospora symmetrica* | NR_158993.1 | 1686 | 99.28% | 1003 | 96% | 0 |
| 136 | FEB | HEXI G MEA | MW723906 | *Papiliotrema flavescens* | KY104461.1 | 917 | 98.10% | 917 | 99% | 0 |
| 137 | FEB | HEPING B MEA | MW723907 | *Aureobasidium pullulans* | MH854941.1 | 1029 | 98.79% | 1029 | 99% | 0 |
| 138 | FEB | HEXI B SDA | MW723908 | *Alternaria tenuissima* | MF952613.1 | 1695 | 96.85% | 1162 | 99% | 0 |
| 139 | FEB | HEBEI G SDA | MW723909 | *Aspergillus niger* | MN420840.1 | 1509 | 99.16% | 1509 | 99% | 0 |
| 140 | FEB | NANKAI G SDA | MW723910 | *Naganishia albida* | KY238185.1 | 1072 | 98.84% | 1072 | 100% | 0 |
| 141 | FEB | HEPING B MEA | MW723911 | *Cladosporium cladosporioides* | LC515098.1 | 1919 | 98.53% | 1919 | 99% | 0 |
| 142 | FEB | HEPING B SDA | MW723912 | *Aureobasidium pullulans* | MH854941.1 | 869 | 100.00% | 869 | 99% | 0 |
| 143 | FEB | HEXI G SDA | MW723913 | *Alternaria alternata* | MF422130.1 | 1228 | 99.30% | 1027 | 100% | 0 |
| 144 | FEB | HEPING B SDA | MW723914 | *Naganishia albida* | KY238161.1 | 1055 | 98.66% | 1055 | 99% | 0 |
| 145 | FEB | HEXI B SDA | MW723915 | Cladosporium sp. | MK367557.1 | 913 | 98.09% | 913 | 99% | 0 |
| 146 | FEB | HEBEI G MEA | MW723916 | *Cladosporium cladosporioides* | HQ671181.1 | 920 | 99.03% | 920 | 99% | 0 |
| 147 | FEB | HEXI G MEA | MW723917 | *Cladosporium cladosporioides* | LC515096.1 | 1905 | 98.43% | 1905 | 99% | 0 |
| 148 | FEB | HEXI B MEA | MW723918 | *Fusarium verticillioides* | MK790051.1 | 1270 | 96.84% | 953 | 98% | 0 |
| 149 | FEB | HEXI B SDA | MW723919 | *Cladosporium perangustum* | KF706664.1 | 931 | 99.42% | 931 | 97% | 0 |
| 150 | FEB | NANKAI G SDA | MW723920 | *Cladosporium cladosporioides* | MK813966.1 | 929 | 97.26% | 929 | 99% | 0 |
| 151 | FEB | NANKAI G SDA | MW723921 | Alternaria sp. | KP027305.1 | 2869 | 95.27% | 1550 | 99% | 0 |
|  |  |  |  | *Alternaria alternata* | JQ080319.1 | 2983 | 94.57% | 1528 | 97% | 0 |
| 152 | FEB | NANKAI G SDA | MW723922 | *Cladosporium cladosporioides* | KX664389.1 | 1934 | 98.80% | 1934 | 99% | 0 |
| 153 | FEB | NANKAI B SDA | MW723923 | *Didymella pedeiae* | MH923258.1 | 1503 | 96.35% | 1079 | 99% | 0 |
| 154 | FEB | NANKAI G MEA | MW723924 | *Cladosporium cladosporioides* | LC515096.1 | 1912 | 98.70% | 1912 | 100% | 0 |
| 155 | FEB | HEXI G MEA | MW723925 | Pleosporales sp. | MH473902.1 | 1551 | 98.84% | 918 | 96% | 0 |
|  |  |  |  | *Periconia epilithographicola* | NR_157477.1 | 1479 | 96.77% | 870 | 97% | 0 |
| 156 | FEB | HEPING B MEA | MW723926 | *Paraconiothyrium hawaiiense* | EU715661.1 | 1614 | 98.60% | 1003 | 89% | 0 |
| 157 | FEB | HEXI G SDA | MW723927 | *Microsphaeropsis olivacea* | MN944412.1 | 854 | 100.00% | 854 | 100% | 0 |
| 158 | FEB | HEPING B MEA | MW723928 | *Erythrobasidium hasegawianum* | AF444522.1 | 998 | 99.82% | 998 | 100% | 0 |
| 159 | FEB | NANKAI G MEA | MW723929 | *Naganishia albida* | KY238161.1 | 1011 | 97.47% | 1011 | 98% | 0 |
| 160 | FEB | NANKAI G SDA | MW723930 | *Naganishia albida* | KY238183.1 | 1055 | 98.66% | 1055 | 99% | 0 |
| 161 | FEB | HEBEI G MEA | MW723931 | Symmetrospora sp. | FJ613120.1 | 924 | 98.66% | 924 | 90% | 0 |
|  |  |  |  | *Symmetrospora foliicola* | KY105571.1 | 915 | 96.26% | 915 | 96% | 0 |
| 162 | FEB | NANKAI G MEA | MW723932 | *Naganishia albida* | KY238170.1 | 1016 | 97.32% | 1016 | 99% | 0 |
| 163 | FEB | NANKAI G MEA | MW723933 | *Naganishia albida* | KY238149.1 | 1013 | 97.32% | 1013 | 99% | 0 |
| 164 | FEB | HEBEI B MEA | MW723934 | *Papiliotrema flavescens* | MH931267.1 | 1393 | 99.35% | 1393 | 99% | 0 |
| 165 | FEB | HEXI G MEA | MW723935 | *Papiliotrema flavescens* | MH931267.1 | 1327 | 97.91% | 1327 | 99% | 0 |
| 1 | MAR | NANKAI B MEA | MW723936 | *Naganishia globosa* | [MH809978.1](https://www.ncbi.nlm.nih.gov/nucleotide/MH809978.1?report=genbank&log$=nucltop&blast_rank=2&RID=BR2J17JZ01R) | 1110 | 99.81% | 966 | 96% | 0 |
| 2 | MAR | NANKAI B MEA | MW723937 | *Naganishia globosa* | [MH809978.1](https://www.ncbi.nlm.nih.gov/nucleotide/MH809978.1?report=genbank&log$=nucltop&blast_rank=1&RID=BR2Z7G5G016) | 950 | 99.62% | 950 | 100% | 0 |
| 3 | MAR | NANKAI B MEA | MW723938 | *Didymella pedeiae* | [MH923258.1](https://www.ncbi.nlm.nih.gov/nucleotide/MH923258.1?report=genbank&log$=nucltop&blast_rank=1&RID=BR35AABK014) | 1365 | 96.67% | 987 | 100% | 0 |
| 4 | MAR | NANKAI B MEA | MW723939 | *Naganishia liquefaciens* | [MT303813.1](https://www.ncbi.nlm.nih.gov/nucleotide/MT303813.1?report=genbank&log$=nucltop&blast_rank=1&RID=BR3CSBP0016) | 933 | 100.00% | 933 | 99% | 0 |
| 5 | MAR | NANKAI B MEA | MW723940 | *Naganishia globosa* | [MH809978.1](https://www.ncbi.nlm.nih.gov/nucleotide/MH809978.1?report=genbank&log$=nucltop&blast_rank=1&RID=BR3JATED016) | 1053 | 99.65% | 1053 | 100% | 0 |
| 6 | MAR | NANKAI B MEA | MW723941 | *Alternaria alternata* | [HQ343446.1](https://www.ncbi.nlm.nih.gov/nucleotide/HQ343446.1?report=genbank&log$=nucltop&blast_rank=1&RID=BR3UAXT0014) | 939 | 100.00% | 939 | 99% | 0 |
| 7 | MAR | NANKAI B MEA | MW723942 | *Didymella pedeiae* | [MH923258.1](https://www.ncbi.nlm.nih.gov/nucleotide/MH923258.1?report=genbank&log$=nucltop&blast_rank=1&RID=BR42FEG8014) | 1462 | 95.26% | 1018 | 100% | 0 |
| 8 | MAR | NANKAI B MEA | MW723943 | *Naganishia globosa* | [MH809978.1](https://www.ncbi.nlm.nih.gov/nucleotide/MH809978.1?report=genbank&log$=nucltop&blast_rank=1&RID=BR4DRYGT014) | 989 | 100.00% | 989 | 100% | 0 |
| 9 | MAR | NANKAI B MEA | MW723944 | *Naganishia globosa* | [KY238197.1](https://www.ncbi.nlm.nih.gov/nucleotide/KY238197.1?report=genbank&log$=nucltop&blast_rank=1&RID=BR48PF3Z016) | 1026 | 99.47% | 1026 | 100% | 0 |
| 10 | MAR | NANKAI B MEA | MW723945 | *Aureobasidium pullulans* | [MK772063.1](https://www.ncbi.nlm.nih.gov/nucleotide/MK772063.1?report=genbank&log$=nucltop&blast_rank=1&RID=BR4MDG4M016) | 909 | 99.80% | 909 | 100% | 0 |
| 11 | MAR | NANKAI B MEA | MW723946 | *Naganishia globosa* | [KY238197.1](https://www.ncbi.nlm.nih.gov/nucleotide/KY238197.1?report=genbank&log$=nucltop&blast_rank=9&RID=BR4W5UT2014) | 1136 | 99.29% | 1009 | 95% | 0 |
| 12 | MAR | NANKAI B MEA | MW723947 | Fungal endophyte isolate | [KP335566.1](https://www.ncbi.nlm.nih.gov/nucleotide/KP335566.1?report=genbank&log$=nucltop&blast_rank=1&RID=BR51758A014) | 881 | 100.00% | 881 | 99% | 0 |
|  |  |  |  | *Aureobasidium pullulans* | [MG025879.1](https://www.ncbi.nlm.nih.gov/nucleotide/MG025879.1?report=genbank&log$=nucltop&blast_rank=39&RID=BR51758A014) | 881 | 100.00% | 881 | 99% | 0 |
| 13 | MAR | NANKAI B MEA | MW723948 | *Naganishia globosa* | [MH809978.1](https://www.ncbi.nlm.nih.gov/nucleotide/MH809978.1?report=genbank&log$=nucltop&blast_rank=2&RID=BR5NEPP9016) | 1042 | 99.82% | 1042 | 85% | 0 |
| 14 | MAR | NANKAI B MEA | MW723949 | *Didymella macrostoma* | [MN944409.1](https://www.ncbi.nlm.nih.gov/nucleotide/MN944409.1?report=genbank&log$=nucltop&blast_rank=1&RID=BR5GJ9YR016) | 730 | 99.02% | 730 | 100% | 0 |
| 15 | MAR | NANKAI B MEA | MW723950 | *Didymella pedeiae* | [MH923258.1](https://www.ncbi.nlm.nih.gov/nucleotide/MH923258.1?report=genbank&log$=nucltop&blast_rank=1&RID=CYNTJFHZ014) | 1562 | 97.87% | 1134 | 99% | 0 |
| 16 | MAR | NANKAI B MEA | MW723951 | [Fungal sp.](https://blast.ncbi.nlm.nih.gov/Blast.cgi) | [KT375712.1](https://www.ncbi.nlm.nih.gov/nucleotide/KT375712.1?report=genbank&log$=nucltop&blast_rank=1&RID=CC4SHJWZ016) | 939 | 100.00% | 939 | 100% | 0 |
|  |  |  |  | *[Paraconiothyrium hawaiiense](https://blast.ncbi.nlm.nih.gov/Blast.cgi)* | [EU715661.1](https://www.ncbi.nlm.nih.gov/nucleotide/EU715661.1?report=genbank&log$=nucltop&blast_rank=2&RID=CC4SHJWZ016) | 939 | 100.00% | 939 | 100% | 0 |
| 17 | MAR | NANKAI B MEA | MW723952 | *Didymella pedeiae* | [MH923258.1](https://www.ncbi.nlm.nih.gov/nucleotide/MH923258.1?report=genbank&log$=nucltop&blast_rank=1&RID=BR5UEV8X014) | 1370 | 96.53% | 990 | 100% | 0 |
| 18 | MAR | NANKAI B MEA | MW723953 | *[Didymellaceae](https://blast.ncbi.nlm.nih.gov/Blast.cgi)* [sp.](https://blast.ncbi.nlm.nih.gov/Blast.cgi) | [MT453292.1](https://www.ncbi.nlm.nih.gov/nucleotide/MT453292.1?report=genbank&log$=nucltop&blast_rank=1&RID=CC4X8ZW6016) | 830 | 100.00% | 830 | 100% | 0 |
| 19 | MAR | NANKAI B MEA | MW723954 | *Naganishia globosa* | [MH809978.1](https://www.ncbi.nlm.nih.gov/nucleotide/MH809978.1?report=genbank&log$=nucltop&blast_rank=1&RID=BR613YJ9014) | 924 | 99.22% | 924 | 100% | 0 |
| 20 | MAR | NANKAI B MEA | MW723955 | *Naganishia globosa* | [MH809978.1](https://www.ncbi.nlm.nih.gov/nucleotide/MH809978.1?report=genbank&log$=nucltop&blast_rank=1&RID=BR613YJ9014) | 924 | 99.22% | 924 | 100% | 0 |
| 21 | MAR | NANKAI B MEA | MW723956 | *Phoma medicaginis* | [KT192426.1](https://www.ncbi.nlm.nih.gov/nucleotide/KT192426.1?report=genbank&log$=nucltop&blast_rank=1&RID=BR6BD09G014) | 846 | 99.78% | 846 | 99% | 0 |
| 22 | MAR | NANKAI B MEA | MW723957 | *[Naganishia globosa](https://blast.ncbi.nlm.nih.gov/Blast.cgi)* | [MH809978.1](https://www.ncbi.nlm.nih.gov/nucleotide/MH809978.1?report=genbank&log$=nucltop&blast_rank=2&RID=CC51JKWC014) | 1237 | 100.00% | 1027 | 96% | 0 |
| 23 | MAR | NANKAI B MEA | MW723958 | *Naganishia albida* | [MG551287.1](https://www.ncbi.nlm.nih.gov/nucleotide/MG551287.1?report=genbank&log$=nucltop&blast_rank=1&RID=BZ67DE26016) | 1703 | 97.25% | 1096 | 100% | 0 |
| 24 | MAR | NANKAI B MEA | MW723959 | *[Phoma](https://blast.ncbi.nlm.nih.gov/Blast.cgi)* [sp.](https://blast.ncbi.nlm.nih.gov/Blast.cgi) | [KY088083.1](https://www.ncbi.nlm.nih.gov/nucleotide/KY088083.1?report=genbank&log$=nucltop&blast_rank=1&RID=CC54Y4R0016) | 839 | 99.78% | 839 | 100% | 0 |
| 25 | MAR | NANKAI B MEA | MW723960 | *[Phoma](https://blast.ncbi.nlm.nih.gov/Blast.cgi)* [sp.](https://blast.ncbi.nlm.nih.gov/Blast.cgi) | [KY088083.1](https://www.ncbi.nlm.nih.gov/nucleotide/KY088083.1?report=genbank&log$=nucltop&blast_rank=1&RID=CC5EB4KW016) | 845 | 100.00% | 845 | 99% | 0 |
| 26 | MAR | NANKAI B MEA | MW723961 | *[Naganishia globosa](https://blast.ncbi.nlm.nih.gov/Blast.cgi)* | [MH809978.1](https://www.ncbi.nlm.nih.gov/nucleotide/MH809978.1?report=genbank&log$=nucltop&blast_rank=2&RID=CC5B955K016) | 891 | 100.00% | 891 | 84% | 0 |
| 27 | MAR | NANKAI B MEA | MW723962 | *Naganishia globosa* | [MH809978.1](https://www.ncbi.nlm.nih.gov/nucleotide/MH809978.1?report=genbank&log$=nucltop&blast_rank=1&RID=CYPDDT4A01R) | 1434 | 97.28% | 1434 | 100% | 0 |
| 28 | MAR | NANKAI B MEA | MW723963 | *[Alternaria tenuissima](https://blast.ncbi.nlm.nih.gov/Blast.cgi)* | [MF952613.1](https://www.ncbi.nlm.nih.gov/nucleotide/MF952613.1?report=genbank&log$=nucltop&blast_rank=1&RID=BZ6FGBR6014) | 1398 | 97.33% | 1020 | 100% | 0 |
| 29 | MAR | NANKAI B MEA | MW723964 | *Paraconiothyrium hawaiiense* | [EU715661.1](https://www.ncbi.nlm.nih.gov/nucleotide/EU715661.1?report=genbank&log$=nucltop&blast_rank=1&RID=CYPVERKZ014) | 2006 | 98.09% | 2006 | 99% | 0 |
| 30 | MAR | NANKAI B MEA | MW723965 | *[Naganishia globosa](https://blast.ncbi.nlm.nih.gov/Blast.cgi)* | [MH809978.1](https://www.ncbi.nlm.nih.gov/nucleotide/MH809978.1?report=genbank&log$=nucltop&blast_rank=1&RID=BZ71DR24014) | 998 | 100.00% | 998 | 99% | 0 |
| 31 | MAR | NANKAI B MEA | MW723966 | *Naganishia globosa* | [MH809978.1](https://www.ncbi.nlm.nih.gov/nucleotide/MH809978.1?report=genbank&log$=nucltop&blast_rank=1&RID=BZ7AVVZ5016) | 963 | 99.81% | 963 | 99% | 0 |
| 32 | MAR | NANKAI B MEA | MW723967 | *[Cryptococcus saitoi](https://blast.ncbi.nlm.nih.gov/Blast.cgi)* | [JQ993394.1](https://www.ncbi.nlm.nih.gov/nucleotide/JQ993394.1?report=genbank&log$=nucltop&blast_rank=1&RID=BZ7J3632014) | 1113 | 99.45% | 983 | 98% | 0 |
|  |  |  |  | *[Naganishia globosa](https://blast.ncbi.nlm.nih.gov/Blast.cgi)* | [MH809978.1](https://www.ncbi.nlm.nih.gov/nucleotide/MH809978.1?report=genbank&log$=nucltop&blast_rank=2&RID=BZ7J3632014) | 1110 | 99.26% | 979 | 99% | 0 |
| 33 | MAR | NANKAI B MEA | MW723968 | *[Phoma macrostoma](https://blast.ncbi.nlm.nih.gov/Blast.cgi)* | [KF293857.1](https://www.ncbi.nlm.nih.gov/nucleotide/KF293857.1?report=genbank&log$=nucltop&blast_rank=1&RID=BZ9NAK4D014) | 837 | 100.00% | 837 | 99% | 0 |
| 34 | MAR | NANKAI B SDA | MW723969 | *[Didymella pedeiae](https://blast.ncbi.nlm.nih.gov/Blast.cgi)* | [MH923258.1](https://www.ncbi.nlm.nih.gov/nucleotide/MH923258.1?report=genbank&log$=nucltop&blast_rank=1&RID=BZ9YSE01014) | 1311 | 97.82% | 944 | 100% | 0 |
| 35 | MAR | NANKAI B SDA | MW723970 | *[Naganishia globosa](https://blast.ncbi.nlm.nih.gov/Blast.cgi)* | [MH809978.1](https://www.ncbi.nlm.nih.gov/nucleotide/MH809978.1?report=genbank&log$=nucltop&blast_rank=1&RID=BZA7N58G014) | 911 | 99.21% | 911 | 99% | 0 |
| 36 | MAR | NANKAI B SDA | MW723971 | [Fungal sp.](https://blast.ncbi.nlm.nih.gov/Blast.cgi) | [KT375712.1](https://www.ncbi.nlm.nih.gov/nucleotide/KT375712.1?report=genbank&log$=nucltop&blast_rank=1&RID=CC5K0HM8016) | 990 | 100.00% | 990 | 100% | 0 |
|  |  |  |  | *[Paraconiothyrium hawaiiense](https://blast.ncbi.nlm.nih.gov/Blast.cgi)* | [EU715661.1](https://www.ncbi.nlm.nih.gov/nucleotide/EU715661.1?report=genbank&log$=nucltop&blast_rank=2&RID=CC5K0HM8016) | 990 | 100.00% | 990 | 100% | 0 |
| 37 | MAR | NANKAI B SDA | MW723972 | *Naganishia globosa* | [MH809978.1](https://www.ncbi.nlm.nih.gov/nucleotide/MH809978.1?report=genbank&log$=nucltop&blast_rank=1&RID=CYRU991N01R) | 905 | 97.54% | 905 | 99% | 0 |
| 38 | MAR | NANKAI B SDA | MW723973 | *[Naganishia globosa](https://blast.ncbi.nlm.nih.gov/Blast.cgi)* | [MH809978.1](https://www.ncbi.nlm.nih.gov/nucleotide/MH809978.1?report=genbank&log$=nucltop&blast_rank=1&RID=BZAFGECM014) | 846 | 98.74% | 846 | 100% | 0 |
| 39 | MAR | NANKAI B SDA | MW723974 | *Naganishia albida* | [MF062213.1](https://www.ncbi.nlm.nih.gov/nucleotide/MF062213.1?report=genbank&log$=nucltop&blast_rank=1&RID=CYRDSXE901R) | 1061 | 98.03% | 1061 | 98% | 0 |
| 40 | MAR | NANKAI B SDA | MW723975 | Uncultured *Phaeosphaeria* | [KC785561.1](https://www.ncbi.nlm.nih.gov/nucleotide/KC785561.1?report=genbank&log$=nucltop&blast_rank=1&RID=CC5R5HJ7014) | 815 | 100.00% | 815 | 99% | 0 |
|  |  |  |  | *[Phaeosphaeria](https://blast.ncbi.nlm.nih.gov/Blast.cgi)* [sp.](https://blast.ncbi.nlm.nih.gov/Blast.cgi) | [HQ631018.1](https://www.ncbi.nlm.nih.gov/nucleotide/HQ631018.1?report=genbank&log$=nucltop&blast_rank=2&RID=CC5R5HJ7014) | 815 | 100.00% | 815 | 99% | 0 |
| 41 | MAR | NANKAI B SDA | MW723976 | *[Naganishia globosa](https://blast.ncbi.nlm.nih.gov/Blast.cgi)* | [MH809978.1](https://www.ncbi.nlm.nih.gov/nucleotide/MH809978.1?report=genbank&log$=nucltop&blast_rank=1&RID=CYS5X5RR01R) | 891 | 96.98% | 891 | 99% | 0 |
| 42 | MAR | NANKAI B SDA | MW723977 | [Uncultured fungus clone](https://blast.ncbi.nlm.nih.gov/Blast.cgi) | [KX516304.1](https://www.ncbi.nlm.nih.gov/nucleotide/KX516304.1?report=genbank&log$=nucltop&blast_rank=1&RID=CYSETC07014) | 1129 | 97.16% | 1129 | 99% | 0 |
|  |  |  |  | *[Naganishia globosa](https://blast.ncbi.nlm.nih.gov/Blast.cgi)* | [MH809978.1](https://www.ncbi.nlm.nih.gov/nucleotide/MH809978.1?report=genbank&log$=nucltop&blast_rank=3&RID=CYSETC07014) | 1123 | 97.16% | 1129 | 99% | 0 |
| 43 | MAR | NANKAI B SDA | MW723978 | [Uncultured fungus clone](https://blast.ncbi.nlm.nih.gov/Blast.cgi) | [KX516304.1](https://www.ncbi.nlm.nih.gov/nucleotide/KX516304.1?report=genbank&log$=nucltop&blast_rank=1&RID=CYSMKDSV01R) | 1096 | 96.26% | 1096 | 99% | 0 |
|  |  |  |  | *[Naganishia globosa](https://blast.ncbi.nlm.nih.gov/Blast.cgi)* | [MH809978.1](https://www.ncbi.nlm.nih.gov/nucleotide/MH809978.1?report=genbank&log$=nucltop&blast_rank=3&RID=CYSMKDSV01R) | 1090 | 96.26% | 1096 | 99% | 0 |
| 44 | MAR | NANKAI B SDA | MW723979 | *[Naganishia albida](https://blast.ncbi.nlm.nih.gov/Blast.cgi)* | [MF062213.1](https://www.ncbi.nlm.nih.gov/nucleotide/MF062213.1?report=genbank&log$=nucltop&blast_rank=1&RID=CC5UF873014) | 961 | 99.81% | 961 | 100% | 0 |
| 45 | MAR | NANKAI B SDA | MW723980 | *[Naganishia liquefaciens](https://blast.ncbi.nlm.nih.gov/Blast.cgi)* | [MT303813.1](https://www.ncbi.nlm.nih.gov/nucleotide/MT303813.1?report=genbank&log$=nucltop&blast_rank=1&RID=CC5X9NAA016) | 966 | 100.00% | 966 | 100% | 0 |
| 46 | MAR | NANKAI B SDA | MW723981 | *Aureobasidium proteae* | [MN341232.1](https://www.ncbi.nlm.nih.gov/nucleotide/MN341232.1?report=genbank&log$=nucltop&blast_rank=1&RID=CYT2CG2F01R) | 948 | 96.20% | 948 | 100% | 0 |
| 47 | MAR | NANKAI B SDA | MW723982 | *Naganishia albida* | [MG551278.1](https://www.ncbi.nlm.nih.gov/nucleotide/MG551278.1?report=genbank&log$=nucltop&blast_rank=1&RID=D2HDP7K7016) | 1027 | 97.98% | 1027 | 100% | 0 |
| 48 | MAR | NANKAI B SDA | MW723983 | *Naganishia albida* | MK782383.1 | 1027 | 98.95% | 1027 | 99% | 0 |
| 49 | MAR | NANKAI B SDA | MW723984 | *Alternaria alternata* | MT453271.1 | 1378 | 98.96% | 1378 | 99% | 0 |
| 50 | MAR | NANKAI B SDA | MW723985 | Uncultured fungus | [MF156091.1](https://www.ncbi.nlm.nih.gov/nucleotide/MF156091.1?report=genbank&log$=nucltop&blast_rank=1&RID=BZNB35M8016) | 874 | 98.98% | 874 | 89% | 0 |
|  |  |  |  | *Alternaria alternata* | [KU258751.1](https://www.ncbi.nlm.nih.gov/nucleotide/KU258751.1?report=genbank&log$=nucltop&blast_rank=5&RID=1FX55Z3W016) | 872 | 98.78% | 872 | 90% | 0 |
| 51 | MAR | NANKAI B SDA | MW723986 | *[Didymella bryoniae](https://blast.ncbi.nlm.nih.gov/Blast.cgi)* | [EU030365.1](https://www.ncbi.nlm.nih.gov/nucleotide/EU030365.1?report=genbank&log$=nucltop&blast_rank=1&RID=BZNRFKCZ014) | 880 | 100.00% | 880 | 99% | 0 |
| 52 | MAR | NANKAI B SDA | MW723987 | *[Alternaria](https://blast.ncbi.nlm.nih.gov/Blast.cgi)* [sp.](https://blast.ncbi.nlm.nih.gov/Blast.cgi) | [MT447542.1](https://www.ncbi.nlm.nih.gov/nucleotide/MT447542.1?report=genbank&log$=nucltop&blast_rank=1&RID=BZNXA9FB016) | 928 | 100.00% | 928 | 100% | 0 |
|  |  |  |  | *[Alternaria alternata](https://blast.ncbi.nlm.nih.gov/Blast.cgi)* | [MT446185.1](https://www.ncbi.nlm.nih.gov/nucleotide/MT446185.1?report=genbank&log$=nucltop&blast_rank=2&RID=BZNXA9FB016) | 928 | 100.00% | 928 | 100% | 0 |
| 53 | MAR | NANKAI B SDA | MW723988 | *[Alternaria alternata](https://blast.ncbi.nlm.nih.gov/Blast.cgi)* | [MK690431.1](https://www.ncbi.nlm.nih.gov/nucleotide/MK690431.1?report=genbank&log$=nucltop&blast_rank=1&RID=CC6AW151014) | 881 | 100.00% | 881 | 100% | 0 |
| 54 | MAR | NANKAI B SDA | MW723989 | *[Alternaria](https://blast.ncbi.nlm.nih.gov/Blast.cgi)* [sp.](https://blast.ncbi.nlm.nih.gov/Blast.cgi) | [MT447542.1](https://www.ncbi.nlm.nih.gov/nucleotide/MT447542.1?report=genbank&log$=nucltop&blast_rank=1&RID=BZBHJXA0016) | 869 | 100.00% | 869 | 100% | 0 |
|  |  |  |  | *[Alternaria alternata](https://blast.ncbi.nlm.nih.gov/Blast.cgi)* | [MT446185.1](https://www.ncbi.nlm.nih.gov/nucleotide/MT446185.1?report=genbank&log$=nucltop&blast_rank=2&RID=BZBHJXA0016) | 869 | 100.00% | 869 | 100% | 0 |
| 55 | MAR | NANKAI B SDA | MW723990 | *Alternaria alternata* | [MT453271.1](https://www.ncbi.nlm.nih.gov/nucleotide/MT453271.1?report=genbank&log$=nucltop&blast_rank=1&RID=CC6E2JC9016) | 785 | 100.00% | 785 | 100% | 0 |
| 56 | MAR | NANKAI B SDA | MW723991 | *[Dothiorella viticola](https://blast.ncbi.nlm.nih.gov/Blast.cgi)* | [KY385661.1](https://www.ncbi.nlm.nih.gov/nucleotide/KY385661.1?report=genbank&log$=nucltop&blast_rank=1&RID=BZP6HC1A016) | 913 | 100.00% | 913 | 98% | 0 |
| 57 | MAR | NANKAI B SDA | MW723992 | *Paraconiothyrium hawaiiense* | [EU715661.1](https://www.ncbi.nlm.nih.gov/nucleotide/EU715661.1?report=genbank&log$=nucltop&blast_rank=1&RID=D2J89S9U016) | 1971 | 97.49% | 1971 | 99% | 0 |
| 58 | MAR | NANKAI B SDA | MW723993 | *[Aplosporella javeedii](https://blast.ncbi.nlm.nih.gov/Blast.cgi)* | [MH974687.1](https://www.ncbi.nlm.nih.gov/nucleotide/MH974687.1?report=genbank&log$=nucltop&blast_rank=1&RID=BZPAZH6E014) | 874 | 97.65% | 874 | 99% | 0 |
| 59 | MAR | NANKAI B SDA | MW723994 | *[Didymellaceae](https://blast.ncbi.nlm.nih.gov/Blast.cgi)* [sp.](https://blast.ncbi.nlm.nih.gov/Blast.cgi) | [MT453292.1](https://www.ncbi.nlm.nih.gov/nucleotide/MT453292.1?report=genbank&log$=nucltop&blast_rank=1&RID=CC6HB2TA014) | 832 | 100.00% | 832 | 99% | 0 |
| 60 | MAR | NANKAI B SDA | MW723995 | *Dothiorella viticola* | [KY385661.1](https://www.ncbi.nlm.nih.gov/nucleotide/KY385661.1?report=genbank&log$=nucltop&blast_rank=1&RID=D2JSVBX1014) | 931 | 97.27% | 931 | 99% | 0 |
| 61 | MAR | NANKAI B SDA | MW723996 | *[Microsphaeropsis olivacea](https://blast.ncbi.nlm.nih.gov/Blast.cgi)* | [MN944412.1](https://www.ncbi.nlm.nih.gov/nucleotide/MN944412.1?report=genbank&log$=nucltop&blast_rank=1&RID=BZPG20Z5016) | 841 | 100.00% | 841 | 99% | 0 |
| 62 | MAR | NANKAI G SDA | MW723997 | *[Alternaria alternata](https://blast.ncbi.nlm.nih.gov/Blast.cgi)* | [MT333205.1](https://www.ncbi.nlm.nih.gov/nucleotide/MT333205.1?report=genbank&log$=nucltop&blast_rank=1&RID=CC7RHGX1016) | 942 | 100.00% | 942 | 100% | 0 |
| 63 | MAR | NANKAI G MEA | MW723998 | *[Cladosporium anthropophilum](https://blast.ncbi.nlm.nih.gov/Blast.cgi)* | [MK965098.1](https://www.ncbi.nlm.nih.gov/nucleotide/MK965098.1?report=genbank&log$=nucltop&blast_rank=1&RID=BZR7D294016) | 898 | 100.00% | 898 | 100% | 0 |
| 64 | MAR | NANKAI G MEA | MW723999 | *[Alternaria alternata](https://blast.ncbi.nlm.nih.gov/Blast.cgi)* | [MK605765.1](https://www.ncbi.nlm.nih.gov/nucleotide/MK605765.1?report=genbank&log$=nucltop&blast_rank=1&RID=CC7UWFDK014) | 898 | 100.00% | 898 | 100% | 0 |
| 65 | MAR | NANKAI G MEA | MW724000 | *[Coniothyrium aleuritis](https://blast.ncbi.nlm.nih.gov/Blast.cgi)* | [MK070113.1](https://www.ncbi.nlm.nih.gov/nucleotide/MK070113.1?report=genbank&log$=nucltop&blast_rank=1&RID=CC80U1H4014) | 743 | 100.00% | 743 | 98% | 0 |
| 66 | MAR | NANKAI G MEA | MW724001 | *[Fusarium tricinctum](https://blast.ncbi.nlm.nih.gov/Blast.cgi)* | [MT180474.1](https://www.ncbi.nlm.nih.gov/nucleotide/MT180474.1?report=genbank&log$=nucltop&blast_rank=1&RID=CC897J5G014) | 843 | 100.00% | 843 | 100% | 0 |
| 67 | MAR | NANKAI G MEA | MW724002 | *[Alternaria alternata](https://blast.ncbi.nlm.nih.gov/Blast.cgi)* | [MT446185.1](https://www.ncbi.nlm.nih.gov/nucleotide/MT446185.1?report=genbank&log$=nucltop&blast_rank=1&RID=X383EVDX016) | 1053 | 98.33% | 1053 | 100% | 0 |
| 68 | MAR | NANKAI G SDA | MW724003 | *Naganishia albida* | [MK782383.1](https://www.ncbi.nlm.nih.gov/nucleotide/MK782383.1?report=genbank&log$=nucltop&blast_rank=1&RID=D2KAVUCB016) | 961 | 96.86% | 961 | 99% | 0 |
| 69 | MAR | NANKAI G SDA | MW724004 | *Naganishia albida* | [MK782383.1](https://www.ncbi.nlm.nih.gov/nucleotide/MK782383.1?report=genbank&log$=nucltop&blast_rank=1&RID=D2KPS221014) | 987 | 97.57% | 987 | 100% | 0 |
| 70 | MAR | HEXI B SDA | MW724005 | *[Dothideomycetes](https://blast.ncbi.nlm.nih.gov/Blast.cgi)* [sp.](https://blast.ncbi.nlm.nih.gov/Blast.cgi) | [MH474085.1](https://www.ncbi.nlm.nih.gov/nucleotide/MH474085.1?report=genbank&log$=nucltop&blast_rank=1&RID=BZRN80FC016) | 979 | 100.00% | 979 | 99% | 0 |
| 71 | MAR | HEXI B SDA | MW724006 | *[Alternaria alternata](https://blast.ncbi.nlm.nih.gov/Blast.cgi)* | [MT453271.1](https://www.ncbi.nlm.nih.gov/nucleotide/MT453271.1?report=genbank&log$=nucltop&blast_rank=1&RID=CC8CH381016) | 920 | 100.00% | 920 | 100% | 0 |
| 72 | MAR | HEXI B SDA | MW724007 | *[Talaromyces funiculosus](https://blast.ncbi.nlm.nih.gov/Blast.cgi)* | [MH590622.1](https://www.ncbi.nlm.nih.gov/nucleotide/MH590622.1?report=genbank&log$=nucltop&blast_rank=1&RID=C1HCDHWH014) | 929 | 100.00% | 929 | 100% | 0 |
| 73 | MAR | HEXI B SDA | MW724008 | *Talaromyces funiculosus* | [MH590622.1](https://www.ncbi.nlm.nih.gov/nucleotide/MH590622.1?report=genbank&log$=nucltop&blast_rank=1&RID=D2M1ZMP5016) | 970 | 96.74% | 970 | 100% | 0 |
| 74 | MAR | HEXI B SDA | MW724009 | *Phoma* sp. | [KY088083.1](https://www.ncbi.nlm.nih.gov/nucleotide/KY088083.1?report=genbank&log$=nucltop&blast_rank=2&RID=D2MDN170016) | 774 | 95.46% | 774 | 100% | 0 |
| 75 | MAR | HEXI B SDA | MW724010 | *Cladosporium gossypiicola* | [MK956909.1](https://www.ncbi.nlm.nih.gov/nucleotide/MK956909.1?report=genbank&log$=nucltop&blast_rank=1&RID=D2MTY8JY014) | 870 | 97.10% | 870 | 99% | 0 |
| 76 | MAR | HEXI B SDA | MW724011 | *Phoma* sp. | [KY088083.1](https://www.ncbi.nlm.nih.gov/nucleotide/KY088083.1?report=genbank&log$=nucltop&blast_rank=1&RID=D2NMMN7U016) | 791 | 96.08% | 791 | 100% | 0 |
| 77 | MAR | HEXI B MEA | MW724012 | *Naganishia globosa* | [MH809978.1](https://www.ncbi.nlm.nih.gov/nucleotide/MH809978.1?report=genbank&log$=nucltop&blast_rank=1&RID=D2NX1P9W016) | 1450 | 97.63% | 1450 | 100% | 0 |
| 78 | MAR | HEXI B MEA | MW724013 | *[Alternaria alternata](https://blast.ncbi.nlm.nih.gov/Blast.cgi)* | [MK690432.1](https://www.ncbi.nlm.nih.gov/nucleotide/MK690432.1?report=genbank&log$=nucltop&blast_rank=1&RID=CC8REZN1016) | 883 | 100.00% | 883 | 100% | 0 |
| 79 | MAR | HEXI B MEA | MW724014 | *[Alternaria arborescens](https://blast.ncbi.nlm.nih.gov/Blast.cgi)* | [MT420628.1](https://www.ncbi.nlm.nih.gov/nucleotide/MT420628.1?report=genbank&log$=nucltop&blast_rank=1&RID=CC8WG21M014) | 915 | 100.00% | 915 | 100% | 0 |
| 80 | MAR | HEXI B MEA | MW724015 | *[Alternaria alternata](https://blast.ncbi.nlm.nih.gov/Blast.cgi)* | [KX622113.1](https://www.ncbi.nlm.nih.gov/nucleotide/KX622113.1?report=genbank&log$=nucltop&blast_rank=1&RID=CC9111H6016) | 881 | 100.00% | 881 | 100% | 0 |
| 81 | MAR | HEXI G SDA | MW724016 | *[Alternaria alternata](https://blast.ncbi.nlm.nih.gov/Blast.cgi)* | [MT447475.1](https://www.ncbi.nlm.nih.gov/nucleotide/MT447475.1?report=genbank&log$=nucltop&blast_rank=1&RID=C1HTZ5U9014) | 928 | 100.00% | 928 | 99% | 0 |
| 82 | MAR | HEPING B MEA | MW724017 | *Fusarium equiseti* | [MK621018.1](https://www.ncbi.nlm.nih.gov/nucleotide/MK621018.1?report=genbank&log$=nucltop&blast_rank=1&RID=D2SYF3GK014) | 922 | 97.24% | 922 | 99% | 0 |
| 83 | MAR | HEPING B MEA | MW724018 | *[Cladosporium](https://blast.ncbi.nlm.nih.gov/Blast.cgi)* [sp.](https://blast.ncbi.nlm.nih.gov/Blast.cgi) | [MK356562.1](https://www.ncbi.nlm.nih.gov/nucleotide/MK356562.1?report=genbank&log$=nucltop&blast_rank=1&RID=CC995XTY014) | 887 | 100.00% | 887 | 100% | 0 |
|  |  |  |  | *[Cladosporium cladosporioides](https://blast.ncbi.nlm.nih.gov/Blast.cgi)* | [KX639814.1](https://www.ncbi.nlm.nih.gov/nucleotide/KX639814.1?report=genbank&log$=nucltop&blast_rank=2&RID=CC995XTY014) | 887 | 100.00% | 887 | 100% | 0 |
| 84 | MAR | HEPING B MEA | MW724019 | *[Cladosporium anthropophilum](https://blast.ncbi.nlm.nih.gov/Blast.cgi)* | [MN857898.1](https://www.ncbi.nlm.nih.gov/nucleotide/MN857898.1?report=genbank&log$=nucltop&blast_rank=1&RID=C1HZPWGV014) | 902 | 100.00% | 902 | 99% | 0 |
| 85 | MAR | HEPING B MEA | MW724020 | *[Cladosporium gossypiicola](https://blast.ncbi.nlm.nih.gov/Blast.cgi)* | [MK956909.1](https://www.ncbi.nlm.nih.gov/nucleotide/MK956909.1?report=genbank&log$=nucltop&blast_rank=1&RID=C1J6UTA7014) | 863 | 100.00% | 863 | 100% | 0 |
| 86 | MAR | HEPING B MEA | MW724021 | *[Naganishia uzbekistanensis](https://blast.ncbi.nlm.nih.gov/Blast.cgi)* | [MG720264.1](https://www.ncbi.nlm.nih.gov/nucleotide/MG720264.1?report=genbank&log$=nucltop&blast_rank=1&RID=CC9E9S2C014) | 1185 | 99.81% | 963 | 92% | 0 |
| 87 | MAR | HEPING B MEA | MW724022 | *[Alternaria alternata](https://blast.ncbi.nlm.nih.gov/Blast.cgi)* | [MT453271.1](https://www.ncbi.nlm.nih.gov/nucleotide/MT453271.1?report=genbank&log$=nucltop&blast_rank=1&RID=CC9GX4EA016) | 756 | 100.00% | 756 | 100% | 0 |
| 88 | MAR | HEPING B MEA | MW724023 | *[Alternaria alternata](https://blast.ncbi.nlm.nih.gov/Blast.cgi)* | [MT453271.1](https://www.ncbi.nlm.nih.gov/nucleotide/MT453271.1?report=genbank&log$=nucltop&blast_rank=1&RID=CC9KU6AN014) | 900 | 100.00% | 900 | 100% | 0 |
| 89 | MAR | HEPING B SDA | MW724024 | *[Naganishia albida](https://blast.ncbi.nlm.nih.gov/Blast.cgi)* | [MG551274.1](https://www.ncbi.nlm.nih.gov/nucleotide/MG551274.1?report=genbank&log$=nucltop&blast_rank=1&RID=CC9PB8C9014) | 1485 | 96.21% | 1029 | 99% | 0 |
| 90 | MAR | HEPING B SDA | MW724025 | *[Phoma medicaginis](https://blast.ncbi.nlm.nih.gov/Blast.cgi)* | [KT192426.1](https://www.ncbi.nlm.nih.gov/nucleotide/KT192426.1?report=genbank&log$=nucltop&blast_rank=1&RID=CC9TCNHY016) | 907 | 100.00% | 907 | 99% | 0 |
| 91 | MAR | HEPING B SDA | MW724026 | *Naganishia adeliensis* | [KY073545.1](https://www.ncbi.nlm.nih.gov/nucleotide/KY073545.1?report=genbank&log$=nucltop&blast_rank=1&RID=D2PEFXNC01R) | 979 | 97.72% | 979 | 100% | 0 |
| 92 | MAR | HEBEI B SDA | MW724027 | *[Naganishia adeliensis](https://blast.ncbi.nlm.nih.gov/Blast.cgi)* | [KY073545.1](https://www.ncbi.nlm.nih.gov/nucleotide/KY073545.1?report=genbank&log$=nucltop&blast_rank=1&RID=YSAWYGAS016) | 996 | 98.24% | 996 | 100% | 0 |
| 93 | MAR | HEBEI B SDA | MW724028 | *Naganishia globosa* | [MH809978.1](https://www.ncbi.nlm.nih.gov/nucleotide/MH809978.1?report=genbank&log$=nucltop&blast_rank=1&RID=D2R57NW001R) | 1445 | 97.51% | 1445 | 100% | 0 |
| 94 | MAR | HEBEI B SDA | MW724029 | *[Phoma medicaginis](https://blast.ncbi.nlm.nih.gov/Blast.cgi)* | [KT192426.1](https://www.ncbi.nlm.nih.gov/nucleotide/KT192426.1?report=genbank&log$=nucltop&blast_rank=1&RID=CCAEG0MP016) | 817 | 100.00% | 817 | 99% | 0 |
| 95 | MAR | HEBEI B SDA | MW724030 | *Papiliotrema laurentii* | [MK268144.1](https://www.ncbi.nlm.nih.gov/nucleotide/MK268144.1?report=genbank&log$=nucltop&blast_rank=1&RID=D2TAR5B6016) | 989 | 98.06% | 989 | 100% | 0 |
| 96 | MAR | HEBEI B SDA | MW724031 | *Aureobasidium pullulans* | [MK794596.1](https://www.ncbi.nlm.nih.gov/nucleotide/MK794596.1?report=genbank&log$=nucltop&blast_rank=3&RID=CCA9BS5E016) | 872 | 100.00% | 872 | 99% | 0 |
| 97 | MAR | HEBEI B SDA | MW724032 | *[Dothiorella gregaria](https://blast.ncbi.nlm.nih.gov/Blast.cgi)* | [EU520055.1](https://www.ncbi.nlm.nih.gov/nucleotide/EU520055.1?report=genbank&log$=nucltop&blast_rank=1&RID=C1K8GD0G016) | 905 | 100.00% | 905 | 99% | 0 |
| 98 | MAR | HEBEI B SDA | MW724033 | *[Papiliotrema laurentii](https://blast.ncbi.nlm.nih.gov/Blast.cgi)* | [MK268144.1](https://www.ncbi.nlm.nih.gov/nucleotide/MK268144.1?report=genbank&log$=nucltop&blast_rank=1&RID=C1KGTXAD016) | 905 | 99.80% | 905 | 99% | 0 |
| 99 | MAR | HEBEI B SDA | MW724034 | *[Naganishia globosa](https://blast.ncbi.nlm.nih.gov/Blast.cgi)* | [KY238197.1](https://www.ncbi.nlm.nih.gov/nucleotide/KY238197.1?report=genbank&log$=nucltop&blast_rank=1&RID=C1KSH3A3016) | 955 | 100.00% | 955 | 100% | 0 |
| 100 | MAR | HEBEI B SDA | MW724035 | *[Naganishia globosa](https://blast.ncbi.nlm.nih.gov/Blast.cgi)* | [MH809978.1](https://www.ncbi.nlm.nih.gov/nucleotide/MH809978.1?report=genbank&log$=nucltop&blast_rank=2&RID=C1M0TAGC014) | 959 | 99.43% | 959 | 99% | 0 |
| 101 | MAR | HEBEI B SDA | MW724036 | *[Cryptococcus saitoi](https://blast.ncbi.nlm.nih.gov/Blast.cgi)* | [JQ993394.1](https://www.ncbi.nlm.nih.gov/nucleotide/JQ993394.1?report=genbank&log$=nucltop&blast_rank=1&RID=C1M5VC8X014) | 1003 | 98.42% | 1003 | 99% | 0 |
|  |  |  |  | *[Naganishia friedmannii](https://blast.ncbi.nlm.nih.gov/Blast.cgi)* | [MK782318.1](https://www.ncbi.nlm.nih.gov/nucleotide/MK782318.1?report=genbank&log$=nucltop&blast_rank=2&RID=C1M5VC8X014) | 1000 | 98.25% | 1000 | 100% | 0 |
| 102 | MAR | HEBEI B SDA | MW724037 | *[Naganishia globosa](https://blast.ncbi.nlm.nih.gov/Blast.cgi)* | [MH809978.1](https://www.ncbi.nlm.nih.gov/nucleotide/MH809978.1?report=genbank&log$=nucltop&blast_rank=1&RID=C1MAWPEN014) | 979 | 99.81% | 979 | 99% | 0 |
| 103 | MAR | HEBEI B SDA | MW724038 | *Fusarium equiseti* | [MK621018.1](https://www.ncbi.nlm.nih.gov/nucleotide/MK621018.1?report=genbank&log$=nucltop&blast_rank=1&RID=D2SYF3GK014) | 957 | 98.18% | 957 | 100% | 0 |
| 104 | MAR | HEBEI B SDA | MW724039 | *[Naganishia albida](https://blast.ncbi.nlm.nih.gov/Blast.cgi)* | [MG551288.1](https://www.ncbi.nlm.nih.gov/nucleotide/MG551288.1?report=genbank&log$=nucltop&blast_rank=1&RID=C1MPED9D014) | 1040 | 100.00% | 1040 | 99% | 0 |
| 105 | MAR | HEBEI B SDA | MW724040 | *Phoma sojicola* | [MH857118.1](https://www.ncbi.nlm.nih.gov/nucleotide/MH857118.1?report=genbank&log$=nucltop&blast_rank=5&RID=D2TZG4ZE016) | 850 | 98.77% | 850 | 99% | 0 |
| 106 | MAR | HEBEI B SDA | MW724041 | *[Phoma](https://blast.ncbi.nlm.nih.gov/Blast.cgi)* [sp.](https://blast.ncbi.nlm.nih.gov/Blast.cgi) | [KP067267.1](https://www.ncbi.nlm.nih.gov/nucleotide/KP067267.1?report=genbank&log$=nucltop&blast_rank=1&RID=C1MZGGNV014) | 869 | 99.58% | 869 | 98% | 0 |
|  |  |  |  | Stagonosporopsis cucurbitacearum | [MT312750.1](https://www.ncbi.nlm.nih.gov/nucleotide/MT312750.1?report=genbank&log$=nucltop&blast_rank=2&RID=C1MZGGNV014) | 867 | 99.58% | 867 | 98% | 0 |
| 107 | MAR | HEBEI B SDA | MW724042 | *Cytospora chrysosperma* | [KC880155.1](https://www.ncbi.nlm.nih.gov/nucleotide/KC880155.1?report=genbank&log$=nucltop&blast_rank=1&RID=CCAK2P8V014) | 929 | 100.00% | 929 | 100% | 0 |
| 108 | MAR | HEBEI B SDA | MW724043 | *[Alternaria](https://blast.ncbi.nlm.nih.gov/Blast.cgi)* [sp.](https://blast.ncbi.nlm.nih.gov/Blast.cgi) | [MT447542.1](https://www.ncbi.nlm.nih.gov/nucleotide/MT447542.1?report=genbank&log$=nucltop&blast_rank=1&RID=C1N54W7R014) | 867 | 100.00% | 867 | 100% | 0 |
|  |  |  |  | *[Alternaria alternata](https://blast.ncbi.nlm.nih.gov/Blast.cgi)* | [MT446185.1](https://www.ncbi.nlm.nih.gov/nucleotide/MT446185.1?report=genbank&log$=nucltop&blast_rank=2&RID=C1N54W7R014) | 867 | 100.00% | 867 | 100% | 0 |
| 109 | MAR | HEBEI B SDA | MW724044 | *[Alternaria](https://blast.ncbi.nlm.nih.gov/Blast.cgi)* [sp.](https://blast.ncbi.nlm.nih.gov/Blast.cgi) | [KU377193.1](https://www.ncbi.nlm.nih.gov/nucleotide/KU377193.1?report=genbank&log$=nucltop&blast_rank=1&RID=C1NC2DJW014) | 749 | 98.36% | 749 | 84% | 0 |
|  |  |  |  | *[Alternaria tenuissima](https://blast.ncbi.nlm.nih.gov/Blast.cgi)* | [KF996903.1](https://www.ncbi.nlm.nih.gov/nucleotide/KF996903.1?report=genbank&log$=nucltop&blast_rank=2&RID=C1NC2DJW014) | 749 | 98.36% | 749 | 84% | 0 |
| 110 | MAR | HEBEI B SDA | MW724045 | *[Microsphaeropsis olivacea](https://blast.ncbi.nlm.nih.gov/Blast.cgi)* | [MN944412.1](https://www.ncbi.nlm.nih.gov/nucleotide/MN944412.1?report=genbank&log$=nucltop&blast_rank=1&RID=C1NGAPDU016) | 797 | 100.00% | 797 | 100% | 0 |
| 111 | MAR | HEBEI B SDA | MW724046 | *Cladosporium anthropophilum* | [MN857898.1](https://www.ncbi.nlm.nih.gov/nucleotide/MN857898.1?report=genbank&log$=nucltop&blast_rank=1&RID=D4HTG9H8016) | 913 | 97.05% | 913 | 100% | 0 |
| 112 | MAR | HEBEI B SDA | MW724047 | *[Aureobasidium namibiae](https://blast.ncbi.nlm.nih.gov/Blast.cgi)* | [MK782292.1](https://www.ncbi.nlm.nih.gov/nucleotide/MK782292.1?report=genbank&log$=nucltop&blast_rank=1&RID=C1NNVMXS016) | 795 | 99.77% | 795 | 100% | 0 |
| 113 | MAR | HEBEI B SDA | MW724048 | *[Colletotrichum gloeosporioides](https://blast.ncbi.nlm.nih.gov/Blast.cgi)* | [HQ874970.1](https://www.ncbi.nlm.nih.gov/nucleotide/HQ874970.1?report=genbank&log$=nucltop&blast_rank=1&RID=C1NUZC7C014) | 71.3 | 100.00% | 71.3 | 76% | 0.00E+00 |
| 114 | MAR | HEBEI B MEA | MW724049 | *[Naganishia globosa](https://blast.ncbi.nlm.nih.gov/Blast.cgi)* | [MH809978.1](https://www.ncbi.nlm.nih.gov/nucleotide/MH809978.1?report=genbank&log$=nucltop&blast_rank=1&RID=C1NYHX6R014) | 953 | 99.81% | 953 | 99% | 0 |
| 115 | MAR | HEBEI B MEA | MW724050 | *[Cryptococcus saitoi](https://blast.ncbi.nlm.nih.gov/Blast.cgi)* | [JQ993394.1](https://www.ncbi.nlm.nih.gov/nucleotide/JQ993394.1?report=genbank&log$=nucltop&blast_rank=1&RID=C1PBPAC5016) | 974 | 100.00% | 974 | 99% | 0 |
|  |  |  |  | *[Naganishia globosa](https://blast.ncbi.nlm.nih.gov/Blast.cgi)* | [MH809978.1](https://www.ncbi.nlm.nih.gov/nucleotide/MH809978.1?report=genbank&log$=nucltop&blast_rank=2&RID=C1PBPAC5016) | 966 | 99.81% | 966 | 99% | 0 |
| 116 | MAR | HEBEI B MEA | MW724051 | *[Cladosporium anthropophilum](https://blast.ncbi.nlm.nih.gov/Blast.cgi)* | [MN857898.1](https://www.ncbi.nlm.nih.gov/nucleotide/MN857898.1?report=genbank&log$=nucltop&blast_rank=1&RID=C1PFZ7VK016) | 894 | 100.00% | 894 | 100% | 0 |
| 117 | MAR | HEBEI B MEA | MW724052 | *[Aureobasidium namibiae](https://blast.ncbi.nlm.nih.gov/Blast.cgi)* | [MK782292.1](https://www.ncbi.nlm.nih.gov/nucleotide/MK782292.1?report=genbank&log$=nucltop&blast_rank=1&RID=C1PKX498014) | 806 | 100.00% | 806 | 99% | 0 |
| 118 | MAR | HEBEI B MEA | MW724053 | *[Aureobasidium proteae](https://blast.ncbi.nlm.nih.gov/Blast.cgi)* | [MN341232.1](https://www.ncbi.nlm.nih.gov/nucleotide/MN341232.1?report=genbank&log$=nucltop&blast_rank=1&RID=C1PRA5VN016) | 828 | 100.00% | 828 | 99% | 0 |
| 119 | MAR | HEBEI B MEA | MW724054 | *[Alternaria alternata](https://blast.ncbi.nlm.nih.gov/Blast.cgi)* | [MN889427.1](https://www.ncbi.nlm.nih.gov/nucleotide/MN889427.1?report=genbank&log$=nucltop&blast_rank=1&RID=C1PVVPB0014) | 913 | 100.00% | 913 | 100% | 0 |
| 120 | MAR | HEBEI B MEA | MW724055 | *[Microsphaeropsis olivacea](https://blast.ncbi.nlm.nih.gov/Blast.cgi)* | [MN944412.1](https://www.ncbi.nlm.nih.gov/nucleotide/MN944412.1?report=genbank&log$=nucltop&blast_rank=1&RID=C1R0N0XB016) | 804 | 100.00% | 804 | 100% | 0 |
| 121 | MAR | HEBEI B MEA | MW724056 | *[Naganishia globosa](https://blast.ncbi.nlm.nih.gov/Blast.cgi)* | [MH809978.1](https://www.ncbi.nlm.nih.gov/nucleotide/MH809978.1?report=genbank&log$=nucltop&blast_rank=1&RID=C1R674FX014) | 1037 | 99.82% | 1037 | 100% | 0 |
| 122 | MAR | HEBEI B MEA | MW724057 | *[Cladosporium anthropophilum](https://blast.ncbi.nlm.nih.gov/Blast.cgi)* | [MN857898.1](https://www.ncbi.nlm.nih.gov/nucleotide/MN857898.1?report=genbank&log$=nucltop&blast_rank=1&RID=C1R9T38N016) | 828 | 100.00% | 828 | 100% | 0 |
| 123 | MAR | HEBEI G SDA | MW724058 | *[Allophoma labilis](https://blast.ncbi.nlm.nih.gov/Blast.cgi)* | [KT013227.1](https://www.ncbi.nlm.nih.gov/nucleotide/KT013227.1?report=genbank&log$=nucltop&blast_rank=1&RID=C1T8RJXX016) | 1454 | 98.71% | 961 | 100% | 0 |
| 124 | MAR | HEBEI G SDA | MW724059 | *Aureobasidium proteae* | [MN341232.1](https://www.ncbi.nlm.nih.gov/nucleotide/MN341232.1?report=genbank&log$=nucltop&blast_rank=1&RID=D4JCVRVZ016) | 992 | 97.59% | 992 | 99% | 0 |
| 125 | MAR | HEBEI G SDA | MW724060 | *[Rhodotorula mucilaginosa](https://blast.ncbi.nlm.nih.gov/Blast.cgi)* | [MN945314.1](https://www.ncbi.nlm.nih.gov/nucleotide/MN945314.1?report=genbank&log$=nucltop&blast_rank=1&RID=C1TDRKFZ014) | 955 | 100.00% | 955 | 100% | 0 |
| 126 | MAR | HEBEI G SDA | MW724061 | *[Alternaria alternata](https://blast.ncbi.nlm.nih.gov/Blast.cgi)* | [KU258751.1](https://www.ncbi.nlm.nih.gov/nucleotide/KU258751.1?report=genbank&log$=nucltop&blast_rank=1&RID=C1THYBZS014) | 909 | 100.00% | 909 | 100% | 0 |
| 127 | MAR | HEBEI G SDA | MW724062 | *[Alternaria](https://blast.ncbi.nlm.nih.gov/Blast.cgi)* [sp.](https://blast.ncbi.nlm.nih.gov/Blast.cgi) | [MT447542.1](https://www.ncbi.nlm.nih.gov/nucleotide/MT447542.1?report=genbank&log$=nucltop&blast_rank=1&RID=C1TP2HGS014) | 782 | 100.00% | 782 | 99% | 0 |
|  |  |  |  | *[Alternaria alternata](https://blast.ncbi.nlm.nih.gov/Blast.cgi)* | [MT446185.1](https://www.ncbi.nlm.nih.gov/nucleotide/MT446185.1?report=genbank&log$=nucltop&blast_rank=2&RID=C1TP2HGS014) | 782 | 100.00% | 782 | 99% | 0 |
| 128 | MAR | HEBEI G SDA | MW724063 | *Paraconiothyrium archidendri* | [NR_155630.1](https://www.ncbi.nlm.nih.gov/nucleotide/NR_155630.1?report=genbank&log$=nucltop&blast_rank=1&RID=D4JP37M7016) | 1000 | 96.38% | 1000 | 100% | 0 |
| 129 | MAR | HEBEI G SDA | MW724064 | *[Alternaria alternata](https://blast.ncbi.nlm.nih.gov/Blast.cgi)* | [MN889427.1](https://www.ncbi.nlm.nih.gov/nucleotide/MN889427.1?report=genbank&log$=nucltop&blast_rank=1&RID=C1TUXETH014) | 957 | 100.00% | 957 | 100% | 0 |
| 130 | MAR | HEBEI G SDA | MW724065 | *Talaromyces funiculosus* | [CP036229.1](https://www.ncbi.nlm.nih.gov/nucleotide/CP036229.1?report=genbank&log$=nucltop&blast_rank=1&RID=D4K2WPZR014) | 4571 | 98.86% | 1249 | 100% | 0 |
| 131 | MAR | HEBEI G SDA | MW724066 | *[Alternaria porri](https://blast.ncbi.nlm.nih.gov/Blast.cgi)* | [MH014943.1](https://www.ncbi.nlm.nih.gov/nucleotide/MH014943.1?report=genbank&log$=nucltop&blast_rank=1&RID=C1U00ZT0014) | 952 | 100.00% | 952 | 100% | 0 |
| 132 | MAR | HEBEI G SDA | MW724067 | *[Alternaria](https://blast.ncbi.nlm.nih.gov/Blast.cgi)* [sp.](https://blast.ncbi.nlm.nih.gov/Blast.cgi) | [MK640595.1](https://www.ncbi.nlm.nih.gov/nucleotide/MK640595.1?report=genbank&log$=nucltop&blast_rank=1&RID=C1U3PN1K016) | 946 | 100.00% | 946 | 100% | 0 |
|  |  |  |  | *[Alternaria](https://blast.ncbi.nlm.nih.gov/Blast.cgi)* [sp.](https://blast.ncbi.nlm.nih.gov/Blast.cgi) | MN856392.1 | 946 | 100.00% | 946 | 100% | 0 |
| 133 | MAR | HEBEI G SDA | MW724068 | [Fungal sp.](https://blast.ncbi.nlm.nih.gov/Blast.cgi) | [KT375712.1](https://www.ncbi.nlm.nih.gov/nucleotide/KT375712.1?report=genbank&log$=nucltop&blast_rank=1&RID=C1U7R5EC014) | 979 | 99.08% | 979 | 100% | 0 |
|  |  |  |  | *[Paraconiothyrium hawaiiense](https://blast.ncbi.nlm.nih.gov/Blast.cgi)* | [EU715661.1](https://www.ncbi.nlm.nih.gov/nucleotide/EU715661.1?report=genbank&log$=nucltop&blast_rank=2&RID=C1U7R5EC014) | 977 | 99.08% | 977 | 99% | 0 |
| 134 | MAR | HEBEI G SDA | MW724069 | *Talaromyces marneffei* | [CP015870.1](https://www.ncbi.nlm.nih.gov/nucleotide/CP015870.1?report=genbank&log$=nucltop&blast_rank=1&RID=D4KEG1YP016) | 1086 | 97.78% | 1086 | 100% | 0 |
| 135 | MAR | HEBEI G SDA | MW724070 | *[Talaromyces marneffei](https://blast.ncbi.nlm.nih.gov/Blast.cgi)* | [CP045655.1](https://www.ncbi.nlm.nih.gov/nucleotide/CP045655.1?report=genbank&log$=nucltop&blast_rank=1&RID=D4KM7R96016) | 1184 | 98.23% | 1184 | 100% | 0 |
| 136 | MAR | HEBEI G SDA | MW724071 | *[Cladosporium ramotenellum](https://blast.ncbi.nlm.nih.gov/Blast.cgi)* | [MT441591.1](https://www.ncbi.nlm.nih.gov/nucleotide/MT441591.1?report=genbank&log$=nucltop&blast_rank=1&RID=CT6D7E5F01R) | 800 | 100.00% | 800 | 100% | 0 |
| 137 | MAR | HEBEI G SDA | MW724072 | *Dioszegia zsoltii* | [EU266502.1](https://www.ncbi.nlm.nih.gov/nucleotide/EU266502.1?report=genbank&log$=nucltop&blast_rank=1&RID=D86Y7MYV01R) | 896 | 98.62% | 896 | 98% | 0 |
| 138 | MAR | HEBEI G SDA | MW724073 | *Talaromyces marneffei* | [CP045655.1](https://www.ncbi.nlm.nih.gov/nucleotide/CP045655.1?report=genbank&log$=nucltop&blast_rank=1&RID=D87TP3N1016) | 2223 | 98.10% | 1829 | 100% | 0 |
| 139 | MAR | HEBEI G SDA | MW724074 | *[Alternaria alternata](https://blast.ncbi.nlm.nih.gov/Blast.cgi)* | [KU258667.1](https://www.ncbi.nlm.nih.gov/nucleotide/KU258667.1?report=genbank&log$=nucltop&blast_rank=1&RID=BZC18ANM016) | 939 | 100.00% | 939 | 100% | 0 |
| 140 | MAR | HEBEI G MEA | MW724075 | *[Phoma herbarum](https://blast.ncbi.nlm.nih.gov/Blast.cgi)* | [KJ767079.1](https://www.ncbi.nlm.nih.gov/nucleotide/KJ767079.1?report=genbank&log$=nucltop&blast_rank=1&RID=C1VHJC6N014) | 804 | 100.00% | 804 | 99% | 0 |
| 141 | MAR | HEBEI G MEA | MW724076 | *[Phoma](https://blast.ncbi.nlm.nih.gov/Blast.cgi)* [sp.](https://blast.ncbi.nlm.nih.gov/Blast.cgi) | [KF177690.1](https://www.ncbi.nlm.nih.gov/nucleotide/KF177690.1?report=genbank&log$=nucltop&blast_rank=1&RID=C1VU3NTE014) | 880 | 99.79% | 880 | 99% | 0 |
|  |  |  |  | *[Phoma herbarum](https://blast.ncbi.nlm.nih.gov/Blast.cgi)* | [KJ767079.1](https://www.ncbi.nlm.nih.gov/nucleotide/KJ767079.1?report=genbank&log$=nucltop&blast_rank=2&RID=C1VU3NTE014) | 878 | 99.79% | 878 | 99% | 0 |
| 142 | MAR | HEBEI G MEA | MW724077 | *Fusarium equiseti* | [MH999443.1](https://www.ncbi.nlm.nih.gov/nucleotide/MH999443.1?report=genbank&log$=nucltop&blast_rank=1&RID=D88HNZES016) | 1081 | 98.22% | 1081 | 100% | 0 |
| 143 | MAR | HEBEI G MEA | MW724078 | *[Alternaria](https://blast.ncbi.nlm.nih.gov/Blast.cgi)* [sp.](https://blast.ncbi.nlm.nih.gov/Blast.cgi) | [MT447542.1](https://www.ncbi.nlm.nih.gov/nucleotide/MT447542.1?report=genbank&log$=nucltop&blast_rank=1&RID=C1VZNV84014) | 795 | 99.77% | 795 | 100% | 0 |
|  |  |  |  | *[Alternaria alternata](https://blast.ncbi.nlm.nih.gov/Blast.cgi)* | [MT446185.1](https://www.ncbi.nlm.nih.gov/nucleotide/MT446185.1?report=genbank&log$=nucltop&blast_rank=2&RID=C1VZNV84014) | 795 | 99.77% | 795 | 100% | 0 |
| 144 | MAR | HEBEI G MEA | MW724079 | *[Alternaria](https://blast.ncbi.nlm.nih.gov/Blast.cgi)* [sp.](https://blast.ncbi.nlm.nih.gov/Blast.cgi) | [MT447542.1](https://www.ncbi.nlm.nih.gov/nucleotide/MT447542.1?report=genbank&log$=nucltop&blast_rank=1&RID=C1W7MBAM014) | 704 | 99.77% | 795 | 100% | 0 |
|  |  |  |  | *[Alternaria alternata](https://blast.ncbi.nlm.nih.gov/Blast.cgi)* | [MT446185.1](https://www.ncbi.nlm.nih.gov/nucleotide/MT446185.1?report=genbank&log$=nucltop&blast_rank=2&RID=C1W7MBAM014) | 704 | 99.77% | 795 | 100% | 0 |
| 145 | MAR | HEBEI G MEA | MW724080 | *Aureobasidium pullulans* | [MK794595.1](https://www.ncbi.nlm.nih.gov/nucleotide/MK794595.1?report=genbank&log$=nucltop&blast_rank=1&RID=D9BB25HZ016) | 952 | 97.99% | 952 | 100% | 0 |
| 146 | MAR | HEBEI G MEA | MW724081 | *[Alternaria tenuissima](https://blast.ncbi.nlm.nih.gov/Blast.cgi)* | [MF952613.1](https://www.ncbi.nlm.nih.gov/nucleotide/MF952613.1?report=genbank&log$=nucltop&blast_rank=1&RID=BZB8BWR3016) | 1608 | 97.59% | 1142 | 99% | 0 |
| 147 | MAR | HEBEI G MEA | MW724082 | *[Alternaria](https://blast.ncbi.nlm.nih.gov/Blast.cgi)* [sp.](https://blast.ncbi.nlm.nih.gov/Blast.cgi) | [MT447542.1](https://www.ncbi.nlm.nih.gov/nucleotide/MT447542.1?report=genbank&log$=nucltop&blast_rank=1&RID=C1WFU25M014) | 854 | 100.00% | 854 | 100% | 0 |
|  |  |  |  | *[Alternaria alternata](https://blast.ncbi.nlm.nih.gov/Blast.cgi)* | [MT446185.1](https://www.ncbi.nlm.nih.gov/nucleotide/MT446185.1?report=genbank&log$=nucltop&blast_rank=2&RID=C1WFU25M014) | 854 | 100.00% | 854 | 100% | 0 |
| 148 | MAR | HEBEI G MEA | MW724083 | *[Rhodotorula mucilaginosa](https://blast.ncbi.nlm.nih.gov/Blast.cgi)* | [MN945314.1](https://www.ncbi.nlm.nih.gov/nucleotide/MN945314.1?report=genbank&log$=nucltop&blast_rank=1&RID=C3XWSRRT014) | 1925 | 100.00% | 961 | 99% | 0 |
| 149 | MAR | HEBEI G MEA | MW724084 | *[Alternaria tenuissima](https://blast.ncbi.nlm.nih.gov/Blast.cgi)* | [MT497426.1](https://www.ncbi.nlm.nih.gov/nucleotide/MT497426.1?report=genbank&log$=nucltop&blast_rank=1&RID=D9BRZMPW016) | 1771 | 97.72% | 1201 | 100% | 0 |
| 150 | MAR | HEBEI G MEA | MW724085 | *Aureobasidium pullulans* | [MK794596.1](https://www.ncbi.nlm.nih.gov/nucleotide/MK794596.1?report=genbank&log$=nucltop&blast_rank=1&RID=D9C292WA016) | 966 | 98.54% | 966 | 100% | 0 |
| 151 | MAR | HEBEI G MEA | MW724086 | *[Talaromyces marneffei](https://blast.ncbi.nlm.nih.gov/Blast.cgi)* | [CP015870.1](https://www.ncbi.nlm.nih.gov/nucleotide/CP015870.1?report=genbank&log$=nucltop&blast_rank=1&RID=D9DUC43S014) | 4033 | 98.86% | 1873 | 100% | 0 |
| 152 | MAR | HEBEI G MEA | MW724087 | *Cladosporium* sp. | [MN905843.1](https://www.ncbi.nlm.nih.gov/nucleotide/MN905843.1?report=genbank&log$=nucltop&blast_rank=1&RID=D9E6ZTHP016) | 911 | 96.71% | 911 | 100% | 0 |
|  |  |  |  | *[Cladosporium tenuissimum](https://blast.ncbi.nlm.nih.gov/Blast.cgi)* | [MH712245.1](https://www.ncbi.nlm.nih.gov/nucleotide/MH712245.1?report=genbank&log$=nucltop&blast_rank=2&RID=D9E6ZTHP016) | 911 | 96.71% | 911 | 100% | 0 |
| 153 | MAR | HEBEI G MEA | MW724088 | *[Alternaria alternata](https://blast.ncbi.nlm.nih.gov/Blast.cgi)* | [MK690431.1](https://www.ncbi.nlm.nih.gov/nucleotide/MK690431.1?report=genbank&log$=nucltop&blast_rank=1&RID=C3YF2R99014) | 911 | 100.00% | 911 | 100% | 0 |
| 154 | MAR | HEBEI G MEA | MW724089 | *Fusarium equiseti* | [MH999443.1](https://www.ncbi.nlm.nih.gov/nucleotide/MH999443.1?report=genbank&log$=nucltop&blast_rank=1&RID=D9GRPGZX014) | 1075 | 98.06% | 1075 | 100% | 0 |
| 155 | MAR | HEBEI G MEA | MW724090 | *[Alternaria tenuissima](https://blast.ncbi.nlm.nih.gov/Blast.cgi)* | [MN907695.1](https://www.ncbi.nlm.nih.gov/nucleotide/MN907695.1?report=genbank&log$=nucltop&blast_rank=1&RID=C3YKVKV9016) | 952 | 100.00% | 952 | 99% | 0 |
| 156 | MAR | HEBEI G MEA | MW724091 | *[Alternaria alternata](https://blast.ncbi.nlm.nih.gov/Blast.cgi)* | [MT447475.1](https://www.ncbi.nlm.nih.gov/nucleotide/MT447475.1?report=genbank&log$=nucltop&blast_rank=1&RID=C46PZ311014) | 983 | 100.00% | 983 | 100% | 0 |
| 157 | MAR | HEBEI G MEA | MW724092 | *[Cladosporium](https://blast.ncbi.nlm.nih.gov/Blast.cgi)* [sp.](https://blast.ncbi.nlm.nih.gov/Blast.cgi) | [MT000472.1](https://www.ncbi.nlm.nih.gov/nucleotide/MT000472.1?report=genbank&log$=nucltop&blast_rank=1&RID=C3Z53UJG016) | 861 | 100.00% | 861 | 99% | 0 |
| 158 | MAR | HEBEI G MEA | MW724093 | *Naganishia albida* | [MG551274.1](https://www.ncbi.nlm.nih.gov/nucleotide/MG551274.1?report=genbank&log$=nucltop&blast_rank=1&RID=D9H4R7FP016) | 2742 | 98.29% | 1947 | 100% | 0 |
| 159 | MAR | HEBEI G MEA | MW724094 | *Candida auris* | [LR595924.1](https://www.ncbi.nlm.nih.gov/nucleotide/LR595924.1?report=genbank&log$=nucltop&blast_rank=1&RID=3WE67XSV013) | 675 | 97.04% | 675 | 97% | 0 |
| 160 | MAR | HEBEI G MEA | MW724095 | *[Paraconiothyrium hawaiiense](https://blast.ncbi.nlm.nih.gov/Blast.cgi)* | [EU715661.1](https://www.ncbi.nlm.nih.gov/nucleotide/EU715661.1?report=genbank&log$=nucltop&blast_rank=2&RID=C3ZF0NXW016) | 963 | 100.00% | 963 | 100% | 0 |
| 161 | MAR | HEPING G MEA | MW724096 | *[Cladosporium herbarum](https://blast.ncbi.nlm.nih.gov/Blast.cgi)* | [MK919499.1](https://www.ncbi.nlm.nih.gov/nucleotide/MK919499.1?report=genbank&log$=nucltop&blast_rank=1&RID=C3ZSCFS1014) | 912 | 100.00% | 912 | 100% | 0 |
| 162 | MAR | HEPING G MEA | MW724097 | *Vishniacozyma* sp. | [MN450786.1](https://www.ncbi.nlm.nih.gov/nucleotide/MN450786.1?report=genbank&log$=nucltop&blast_rank=8&RID=D9HDSDXV016) | 1851 | 97.43% | 1851 | 95% | 0 |
| 163 | MAR | HEPING G MEA | MW724098 | *Vishniacozyma tephrensis* | [KX078413.1](https://www.ncbi.nlm.nih.gov/nucleotide/KX078413.1?report=genbank&log$=nucltop&blast_rank=11&RID=CCB1JUV0014) | 866 | 100.00% | 866 | 100% | 0 |
| 164 | MAR | HEPING G MEA | MW724099 | *[Dothideomycetes](https://blast.ncbi.nlm.nih.gov/Blast.cgi)* [sp.](https://blast.ncbi.nlm.nih.gov/Blast.cgi) | [MH474085.1](https://www.ncbi.nlm.nih.gov/nucleotide/MH474085.1?report=genbank&log$=nucltop&blast_rank=1&RID=CCBKVR5G014) | 900 | 99.40% | 900 | 100% | 0 |
| 165 | MAR | HEPING G MEA | MW724100 | *Alternaria tenuissima* | [MH374277.1](https://www.ncbi.nlm.nih.gov/nucleotide/MH374277.1?report=genbank&log$=nucltop&blast_rank=1&RID=D9RG4KYE014) | 1605 | 100.00% | 1605 | 100% | 0 |
| 166 | MAR | HEPING G MEA | MW724101 | *Alternaria tenuissima* | [MT497426.1](https://www.ncbi.nlm.nih.gov/nucleotide/MT497426.1?report=genbank&log$=nucltop&blast_rank=1&RID=D9RRYD61014) | 1703 | 96.58% | 1155 | 100% | 0 |
| 167 | MAR | HEPING G MEA | MW724102 | *Naganishia uzbekistanensis* | [MG720264.1](https://www.ncbi.nlm.nih.gov/nucleotide/MG720264.1?report=genbank&log$=nucltop&blast_rank=1&RID=D9S06E1F016) | 983 | 97.73% | 983 | 99% | 0 |
| 168 | MAR | HEPING G MEA | MW724103 | *Naganishia albida* | [MG551274.1](https://www.ncbi.nlm.nih.gov/nucleotide/MG551274.1?report=genbank&log$=nucltop&blast_rank=1&RID=D9SGGRKA014) | 2718 | 98.11% | 1932 | 100% | 0 |
| 169 | MAR | HEXI G MEA | MW724104 | *[Naganishia albida](https://blast.ncbi.nlm.nih.gov/Blast.cgi)* | [MG551274.1](https://www.ncbi.nlm.nih.gov/nucleotide/MG551274.1?report=genbank&log$=nucltop&blast_rank=2&RID=C403G21J016) | 1299 | 97.17% | 1043 | 99% | 0 |
| 170 | MAR | HEXI G MEA | MW724105 | *Naganishia uzbekistanensis* | [MN759033.1](https://www.ncbi.nlm.nih.gov/nucleotide/MN759033.1?report=genbank&log$=nucltop&blast_rank=1&RID=CCC1DHG4016) | 1040 | 100.00% | 1040 | 100% | 0 |
| 171 | MAR | HEXI G MEA | MW724106 | *[Naganishia randhawae](https://blast.ncbi.nlm.nih.gov/Blast.cgi)* | [MK782282.1](https://www.ncbi.nlm.nih.gov/nucleotide/MK782282.1?report=genbank&log$=nucltop&blast_rank=2&RID=CCC543P3014) | 1034 | 97.28% | 931 | 96% | 0 |
| 172 | MAR | HEXI G MEA | MW724107 | *[Cryptococcus saitoi](https://blast.ncbi.nlm.nih.gov/Blast.cgi)* | [JQ993394.1](https://www.ncbi.nlm.nih.gov/nucleotide/JQ993394.1?report=genbank&log$=nucltop&blast_rank=1&RID=C40C3CAS016) | 1251 | 99.83% | 1136 | 98% | 0 |
| 173 | MAR | HEXI G MEA | MW724108 | *Paraconiothyrium hawaiiense* | [EU715661.1](https://www.ncbi.nlm.nih.gov/nucleotide/EU715661.1?report=genbank&log$=nucltop&blast_rank=1&RID=D9T3NA5P016) | 2012 | 98.02% | 2012 | 100% | 0 |
| 174 | MAR | HEXI G MEA | MW724109 | *Fusarium equiseti* | [MK621018.1](https://www.ncbi.nlm.nih.gov/nucleotide/MK621018.1?report=genbank&log$=nucltop&blast_rank=1&RID=X37382X9013) | 976 | 98.90% | 976 | 99% | 0 |
| 175 | MAR | HEPING G SDA | MW724110 | *Fusarium equiseti* | [MH999443.1](https://www.ncbi.nlm.nih.gov/nucleotide/MH999443.1?report=genbank&log$=nucltop&blast_rank=1&RID=D9TWY62E016) | 1016 | 96.59% | 1016 | 99% | 0 |
| 176 | MAR | HEPING G SDA | MW724111 | *[Fusarium equiseti](https://blast.ncbi.nlm.nih.gov/Blast.cgi)* | [KU939062.1](https://www.ncbi.nlm.nih.gov/nucleotide/KU939062.1?report=genbank&log$=nucltop&blast_rank=1&RID=D9U7FCYV014) | 935 | 97.79% | 935 | 99% | 0 |
| 177 | MAR | HEPING G SDA | MW724112 | *Alternaria tenuissima* | MH374277.1 | 1419 | 97.65% | 1031 | 97% | 0 |
| 178 | MAR | HEPING G SDA | MW724113 | *[Nothophoma](https://blast.ncbi.nlm.nih.gov/Blast.cgi)* [sp.](https://blast.ncbi.nlm.nih.gov/Blast.cgi) | [MN737832.1](https://www.ncbi.nlm.nih.gov/nucleotide/MN737832.1?report=genbank&log$=nucltop&blast_rank=1&RID=C40ZNGE5014) | 1105 | 99.58% | 938 | 90% | 0 |
|  |  |  |  | *[Dothiorella](https://blast.ncbi.nlm.nih.gov/Blast.cgi)* [sp.](https://blast.ncbi.nlm.nih.gov/Blast.cgi) | [KX219605.1](https://www.ncbi.nlm.nih.gov/nucleotide/KX219605.1?report=genbank&log$=nucltop&blast_rank=2&RID=C40ZNGE5014) | 1097 | 99.58% | 938 | 90% | 0 |
| 179 | MAR | HEPING B MEA | MW724114 | *[Alternaria](https://blast.ncbi.nlm.nih.gov/Blast.cgi)* [sp.](https://blast.ncbi.nlm.nih.gov/Blast.cgi) | [MT447542.1](https://www.ncbi.nlm.nih.gov/nucleotide/MT447542.1?report=genbank&log$=nucltop&blast_rank=1&RID=C4147JG1014) | 934 | 100.00% | 934 | 100% | 0 |
|  |  |  |  | *[Alternaria alternata](https://blast.ncbi.nlm.nih.gov/Blast.cgi)* | [MT446185.1](https://www.ncbi.nlm.nih.gov/nucleotide/MT446185.1?report=genbank&log$=nucltop&blast_rank=2&RID=C4147JG1014) | 934 | 100.00% | 934 | 100% | 0 |
| 180 | MAR | HEPING B MEA | MW724115 | *[Fusarium equiseti](https://blast.ncbi.nlm.nih.gov/Blast.cgi)* | [KT192259.1](https://www.ncbi.nlm.nih.gov/nucleotide/KT192259.1?report=genbank&log$=nucltop&blast_rank=1&RID=DBRRB9RJ014) | 915 | 96.89% | 915 | 100% | 0 |
| 181 | MAR | HEPING B MEA | MW724116 | *Naganishia albida* | [MN759029.1](https://www.ncbi.nlm.nih.gov/nucleotide/MN759029.1?report=genbank&log$=nucltop&blast_rank=1&RID=DBSHC1J4014) | 1005 | 98.09% | 1005 | 98% | 0 |
| 182 | MAR | HEPING B MEA | MW724117 | *[Dothiorella gregaria](https://blast.ncbi.nlm.nih.gov/Blast.cgi)* | [KT192425.1](https://www.ncbi.nlm.nih.gov/nucleotide/KT192425.1?report=genbank&log$=nucltop&blast_rank=1&RID=C41AWNJA014) | 1029 | 99.77% | 854 | 97% | 0 |
| 183 | MAR | HEPING B MEA | MW724118 | Uncultured fungus | [MT236706.1](https://www.ncbi.nlm.nih.gov/nucleotide/MT236706.1?report=genbank&log$=nucltop&blast_rank=1&RID=C41HCAFH016) | 2383 | 98.34% | 1203 | 100% | 0 |
|  |  |  |  | *[Cladosporium herbarum](https://blast.ncbi.nlm.nih.gov/Blast.cgi)* | [MN486550.1](https://www.ncbi.nlm.nih.gov/nucleotide/MN486550.1?report=genbank&log$=nucltop&blast_rank=3&RID=C41HCAFH016) | 1251 | 99.59% | 944 | 100% | 0 |
| 184 | MAR | HEPING B MEA | MW724119 | *[Pleosporales](https://blast.ncbi.nlm.nih.gov/Blast.cgi)* [sp.](https://blast.ncbi.nlm.nih.gov/Blast.cgi) | [MK753054.1](https://www.ncbi.nlm.nih.gov/nucleotide/MK753054.1?report=genbank&log$=nucltop&blast_rank=1&RID=DBSW6UAP014) | 767 | 95.98% | 767 | 99% | 0 |
|  |  |  |  | *[Herpotrichia striatispora](https://blast.ncbi.nlm.nih.gov/Blast.cgi)* | [MK564736.1](https://www.ncbi.nlm.nih.gov/nucleotide/MK564736.1?report=genbank&log$=nucltop&blast_rank=2&RID=DBSW6UAP014) | 730 | 94.51% | 730 | 99% | 0 |
| 185 | MAR | HEPING B SDA | MW724120 | *[Pleosporales](https://blast.ncbi.nlm.nih.gov/Blast.cgi)* [sp.](https://blast.ncbi.nlm.nih.gov/Blast.cgi) | [MK753054.1](https://www.ncbi.nlm.nih.gov/nucleotide/MK753054.1?report=genbank&log$=nucltop&blast_rank=1&RID=C41VEM35014) | 747 | 96.88% | 747 | 100% | 0 |
|  |  |  |  | *[Herpotrichia striatispora](https://blast.ncbi.nlm.nih.gov/Blast.cgi)* | [MK564736.1](https://www.ncbi.nlm.nih.gov/nucleotide/MK564736.1?report=genbank&log$=nucltop&blast_rank=3&RID=C41VEM35014) | 737 | 96.43% | 737 | 100% | 0 |
| 186 | MAR | HEPING B SDA | MW724121 | *[Valsa sordida](https://blast.ncbi.nlm.nih.gov/Blast.cgi)* | [MK994101.1](https://www.ncbi.nlm.nih.gov/nucleotide/MK994101.1?report=genbank&log$=nucltop&blast_rank=1&RID=C42A9AKD016) | 1029 | 100.00% | 1029 | 100% | 0 |
| 187 | MAR | HEPING B SDA | MW724122 | *[Cladosporium](https://blast.ncbi.nlm.nih.gov/Blast.cgi)* [sp.](https://blast.ncbi.nlm.nih.gov/Blast.cgi) | [MN486549.1](https://www.ncbi.nlm.nih.gov/nucleotide/MN486549.1?report=genbank&log$=nucltop&blast_rank=1&RID=C42HHBX2016) | 973 | 100.00% | 973 | 99% | 0 |
|  |  |  |  | [Fungal](https://blast.ncbi.nlm.nih.gov/Blast.cgi)[sp.](https://blast.ncbi.nlm.nih.gov/Blast.cgi) | [MG265951.1](https://www.ncbi.nlm.nih.gov/nucleotide/MG265951.1?report=genbank&log$=nucltop&blast_rank=2&RID=C42HHBX2016) | 973 | 99.80% | 973 | 100% | 0 |
| 188 | MAR | HEPING B SDA | MW724123 | *[Cladosporium](https://blast.ncbi.nlm.nih.gov/Blast.cgi)* [sp.](https://blast.ncbi.nlm.nih.gov/Blast.cgi) | [MN486549.1](https://www.ncbi.nlm.nih.gov/nucleotide/MN486549.1?report=genbank&log$=nucltop&blast_rank=1&RID=C42T16E7014) | 944 | 100.00% | 944 | 100% | 0 |
|  |  |  |  | *[Damon diadema](https://blast.ncbi.nlm.nih.gov/Blast.cgi)* | [MK629946.1](https://www.ncbi.nlm.nih.gov/nucleotide/MK629946.1?report=genbank&log$=nucltop&blast_rank=2&RID=C42T16E7014) | 944 | 100.00% | 944 | 100% | 0 |
| 189 | MAR | HEPING B SDA | MW724124 | *[Dioszegia zsoltii](https://blast.ncbi.nlm.nih.gov/Blast.cgi)* | [EU266502.1](https://www.ncbi.nlm.nih.gov/nucleotide/EU266502.1?report=genbank&log$=nucltop&blast_rank=1&RID=C48CCS8T016) | 813 | 100.00% | 813 | 100% | 0 |
| 190 | MAR | HEPING B SDA | MW724125 | [Fungal sp.](https://blast.ncbi.nlm.nih.gov/Blast.cgi) | [KJ867412.1](https://www.ncbi.nlm.nih.gov/nucleotide/KJ867412.1?report=genbank&log$=nucltop&blast_rank=1&RID=C48JP5BH014) | 942 | 100.00% | 942 | 97% | 0 |
|  |  |  |  | *[Aureobasidium](https://blast.ncbi.nlm.nih.gov/Blast.cgi)* [sp.](https://blast.ncbi.nlm.nih.gov/Blast.cgi) | [MK782382.1](https://www.ncbi.nlm.nih.gov/nucleotide/MK782382.1?report=genbank&log$=nucltop&blast_rank=2&RID=C48JP5BH014) | 938 | 100.00% | 938 | 97% | 0 |
| 191 | MAR | HEBEI B MEA | MW724126 | Uncultured fungus | [MT236706.1](https://www.ncbi.nlm.nih.gov/nucleotide/MT236706.1?report=genbank&log$=nucltop&blast_rank=1&RID=CCCH1E9J014) | 1786 | 99.60% | 904 | 100% | 0 |
|  |  |  |  | *[Cladosporium herbarum](https://blast.ncbi.nlm.nih.gov/Blast.cgi)* | [MK919499.1](https://www.ncbi.nlm.nih.gov/nucleotide/MK919499.1?report=genbank&log$=nucltop&blast_rank=2&RID=CCCH1E9J014) | 902 | 99.80% | 902 | 99% | 0 |
| 192 | MAR | HEBEI B MEA | MW724127 | *[Alternaria tenuissima](https://blast.ncbi.nlm.nih.gov/Blast.cgi)* | [MT497426.1](https://www.ncbi.nlm.nih.gov/nucleotide/MT497426.1?report=genbank&log$=nucltop&blast_rank=1&RID=DBT8W1C2014) | 1651 | 96.71% | 1144 | 100% | 0 |
| 193 | MAR | HEBEI B MEA | MW724128 | *Epicoccum nigrum* | [MN089646.1](https://www.ncbi.nlm.nih.gov/nucleotide/MN089646.1?report=genbank&log$=nucltop&blast_rank=1&RID=DBTUNFGH016) | 920 | 96.90% | 920 | 100% | 0 |
| 194 | MAR | HEBEI B MEA | MW724129 | *Cladosporium herbarum* | [MK919499.1](https://www.ncbi.nlm.nih.gov/nucleotide/MK919499.1?report=genbank&log$=nucltop&blast_rank=1&RID=DBU7N0NK016) | 961 | 97.84% | 961 | 100% | 0 |
| 195 | MAR | HEBEI B MEA | MW724130 | *Talaromyces marneffei* | [CP015873.1](https://www.ncbi.nlm.nih.gov/nucleotide/CP015873.1?report=genbank&log$=nucltop&blast_rank=1&RID=DBUND144014) | 10451 | 99.29% | 1013 | 100% | 0 |
| 196 | MAR | HEBEI B MEA | MW724131 | *Cladosporium herbarum* | [MK919499.1](https://www.ncbi.nlm.nih.gov/nucleotide/MK919499.1?report=genbank&log$=nucltop&blast_rank=1&RID=DBVBXPS4016) | 963 | 98.36% | 963 | 100% | 0 |
| 197 | MAR | HEBEI B MEA | MW724132 | *Rhodotorula mucilaginosa* | [MN945314.1](https://www.ncbi.nlm.nih.gov/nucleotide/MN945314.1?report=genbank&log$=nucltop&blast_rank=1&RID=DBVVSTXX014) | 1038 | 98.47% | 1038 | 99% | 0 |
| 198 | MAR | HEBEI B MEA | MW724133 | *Thelebolus microsporus* | [MG586989.1](https://www.ncbi.nlm.nih.gov/nucleotide/MG586989.1?report=genbank&log$=nucltop&blast_rank=1&RID=DBW8U2TE014) | 972 | 98.37% | 972 | 100% | 0 |
| 199 | MAR | HEBEI B MEA | MW724134 | *Cladosporium herbarum* | [MK919499.1](https://www.ncbi.nlm.nih.gov/nucleotide/MK919499.1?report=genbank&log$=nucltop&blast_rank=1&RID=DBWNPNZB014) | 983 | 98.56% | 983 | 100% | 0 |
| 200 | MAR | HEBEI B MEA | MW724135 | *[Alternaria alternata](https://blast.ncbi.nlm.nih.gov/Blast.cgi)* | [MF422130.1](https://www.ncbi.nlm.nih.gov/nucleotide/MF422130.1?report=genbank&log$=nucltop&blast_rank=1&RID=DBX5S7F9016) | 1302 | 98.09% | 1092 | 100% | 0 |
| 201 | MAR | HEBEI B MEA | MW724136 | *[Microsphaeropsis olivacea](https://blast.ncbi.nlm.nih.gov/Blast.cgi)* | [MN944412.1](https://www.ncbi.nlm.nih.gov/nucleotide/MN944412.1?report=genbank&log$=nucltop&blast_rank=1&RID=DBXFX6B6016) | 931 | 97.44% | 931 | 99% | 0 |
| 202 | MAR | HEBEI B MEA | MW724137 | *Thelebolus microsporus* | [MG586989.1](https://www.ncbi.nlm.nih.gov/nucleotide/MG586989.1?report=genbank&log$=nucltop&blast_rank=1&RID=CCCNN8X6016) | 904 | 99.80% | 904 | 100% | 0 |
| 203 | MAR | HEBEI B MEA | MW724138 | *Aureobasidium pullulans* | [MK794596.1](https://www.ncbi.nlm.nih.gov/nucleotide/MK794596.1?report=genbank&log$=nucltop&blast_rank=1&RID=DBYN2P1F016) | 928 | 97.26% | 928 | 100% | 0 |
| 204 | MAR | HEBEI B MEA | MW724139 | *Epicoccum nigrum* | [MN089646.1](https://www.ncbi.nlm.nih.gov/nucleotide/MN089646.1?report=genbank&log$=nucltop&blast_rank=1&RID=DBZ86VH3016) | 953 | 98.00% | 953 | 100% | 0 |
| 205 | MAR | HEBEI B MEA | MW724140 | *[Epicoccum nigrum](https://blast.ncbi.nlm.nih.gov/Blast.cgi)* | [MN089646.1](https://www.ncbi.nlm.nih.gov/nucleotide/MN089646.1?report=genbank&log$=nucltop&blast_rank=1&RID=C48RKB4D014) | 852 | 99.77% | 852 | 99% | 0 |
| 206 | MAR | HEBEI B MEA | MW724141 | *[Alternaria alternata](https://blast.ncbi.nlm.nih.gov/Blast.cgi)* | [MG722823.1](https://www.ncbi.nlm.nih.gov/nucleotide/MG722823.1?report=genbank&log$=nucltop&blast_rank=1&RID=C48ZZJCB014) | 1196 | 98.48% | 971 | 99% | 0 |
| 207 | MAR | HEBEI B MEA | MW724142 | *Epicoccum nigrum* | [MK983497.1](https://www.ncbi.nlm.nih.gov/nucleotide/MK983497.1?report=genbank&log$=nucltop&blast_rank=1&RID=DBZZTTD3014) | 948 | 98.16% | 948 | 100% | 0 |
| 208 | MAR | HEXI G MEA | MW724143 | *[Talaromyces marneffei](https://blast.ncbi.nlm.nih.gov/Blast.cgi)* | [CP015870.1](https://www.ncbi.nlm.nih.gov/nucleotide/CP015870.1?report=genbank&log$=nucltop&blast_rank=1&RID=DCBN7AYJ014) | 590 | 97.40% | 590 | 100% | 2.00E-164 |
| 209 | MAR | HEXI G MEA | MW724144 | *Cladosporium anthropophilum* | [MK965098.1](https://www.ncbi.nlm.nih.gov/nucleotide/MK965098.1?report=genbank&log$=nucltop&blast_rank=1&RID=DCC56EE7014) | 976 | 98.55% | 976 | 99% | 0 |
| 210 | MAR | HEXI G MEA | MW724145 | *Rhodotorula mucilaginosa* | [MN945314.1](https://www.ncbi.nlm.nih.gov/nucleotide/MN945314.1?report=genbank&log$=nucltop&blast_rank=1&RID=DCE1YTH5014) | 1024 | 97.81% | 1024 | 100% | 0 |
| 211 | MAR | HEXI B MEA | MW724146 | *[Naganishia albida](https://blast.ncbi.nlm.nih.gov/Blast.cgi)* | [KY238156.1](https://www.ncbi.nlm.nih.gov/nucleotide/KY238156.1?report=genbank&log$=nucltop&blast_rank=1&RID=DCEBYXAE014) | 1059 | 98.50% | 1059 | 99% | 0 |
| 212 | MAR | HEXI B MEA | MW724147 | *[Acrodontium crateriforme](https://blast.ncbi.nlm.nih.gov/Blast.cgi)* | [MK794315.1](https://www.ncbi.nlm.nih.gov/nucleotide/MK794315.1?report=genbank&log$=nucltop&blast_rank=1&RID=C495A9FU014) | 892 | 100.00% | 892 | 99% | 0 |
| 213 | MAR | HEBEI B SDA | MW724148 | *[Naganishia albida](https://blast.ncbi.nlm.nih.gov/Blast.cgi)* | [KY238152.1](https://www.ncbi.nlm.nih.gov/nucleotide/KY238152.1?report=genbank&log$=nucltop&blast_rank=1&RID=DCEZP4RY014) | 1044 | 98.16% | 1044 | 100% | 0 |
| 214 | MAR | HEBEI B SDA | MW724149 | [Fungal sp.](https://blast.ncbi.nlm.nih.gov/Blast.cgi) | [KT375697.1](https://www.ncbi.nlm.nih.gov/nucleotide/KT375697.1?report=genbank&log$=nucltop&blast_rank=1&RID=C49A3ZT5016) | 1025 | 99.38% | 932 | 99% | 0 |
|  |  |  |  | *[Nothophoma](https://blast.ncbi.nlm.nih.gov/Blast.cgi)* [sp.](https://blast.ncbi.nlm.nih.gov/Blast.cgi) | [MN737833.1](https://www.ncbi.nlm.nih.gov/nucleotide/MN737833.1?report=genbank&log$=nucltop&blast_rank=2&RID=C49A3ZT5016) | 1023 | 99.38% | 930 | 99% | 0 |
| 215 | MAR | HEBEI B SDA | MW724150 | *[Microsphaeropsis olivacea](https://blast.ncbi.nlm.nih.gov/Blast.cgi)* | [MN944412.1](https://www.ncbi.nlm.nih.gov/nucleotide/MN944412.1?report=genbank&log$=nucltop&blast_rank=1&RID=C49EUUPW014) | 922 | 99.79% | 922 | 98% | 0 |
| 216 | MAR | HEBEI B SDA | MW724151 | *[Phoma macrostoma](https://blast.ncbi.nlm.nih.gov/Blast.cgi)* | [KF293857.1](https://www.ncbi.nlm.nih.gov/nucleotide/KF293857.1?report=genbank&log$=nucltop&blast_rank=1&RID=C49PKARC014) | 870 | 100.00% | 870 | 100% | 0 |
| 217 | MAR | HEBEI B SDA | MW724152 | *[Phoma macrostoma](https://blast.ncbi.nlm.nih.gov/Blast.cgi)* | [KF293857.1](https://www.ncbi.nlm.nih.gov/nucleotide/KF293857.1?report=genbank&log$=nucltop&blast_rank=1&RID=C49VZHT1014) | 938 | 100.00% | 938 | 100% | 0 |
| 218 | MAR | HEBEI B SDA | MW724153 | *Epicoccum nigrum* | [MK983497.1](https://www.ncbi.nlm.nih.gov/nucleotide/MK983497.1?report=genbank&log$=nucltop&blast_rank=1&RID=DDF9UUYX016) | 931 | 97.61% | 931 | 100% | 0 |
| 219 | MAR | HEBEI B SDA | MW724154 | *Epicoccum nigrum* | [MK983497.1](https://www.ncbi.nlm.nih.gov/nucleotide/MK983497.1?report=genbank&log$=nucltop&blast_rank=1&RID=DDFNS3P5016) | 963 | 98.54% | 963 | 100% | 0 |
| 220 | MAR | HEBEI B SDA | MW724155 | *Talaromyces funiculosus* | [MH590622.1](https://www.ncbi.nlm.nih.gov/nucleotide/MH590622.1?report=genbank&log$=nucltop&blast_rank=1&RID=DDG9KNG4014) | 1007 | 97.93% | 1007 | 99% | 0 |
| 221 | MAR | HEBEI B SDA | MW724156 | *Naganishia albida* | [KY238183.1](https://www.ncbi.nlm.nih.gov/nucleotide/KY238183.1?report=genbank&log$=nucltop&blast_rank=1&RID=DDH0XN1X014) | 1050 | 98.33% | 1050 | 99% | 0 |
| 222 | MAR | HEBEI B SDA | MW724157 | *Naganishia albida* | [KY238183.1](https://www.ncbi.nlm.nih.gov/nucleotide/KY238183.1?report=genbank&log$=nucltop&blast_rank=1&RID=DDHA9P9A014) | 1005 | 96.99% | 1005 | 99% | 0 |
| 223 | MAR | NANKAI B MEA | MW724158 | *[Paraconiothyrium brasiliense](https://blast.ncbi.nlm.nih.gov/Blast.cgi)* | [MK247586.1](https://www.ncbi.nlm.nih.gov/nucleotide/MK247586.1?report=genbank&log$=nucltop&blast_rank=1&RID=C4B5RV03016) | 997 | 99.80% | 997 | 99% | 0 |
| 224 | MAR | NANKAI B MEA | MW724159 | *Dothiorella viticola* | [KY385661.1](https://www.ncbi.nlm.nih.gov/nucleotide/KY385661.1?report=genbank&log$=nucltop&blast_rank=1&RID=DDHRV82R016) | 929 | 96.94% | 929 | 99% | 0 |
| 225 | MAR | NANKAI B MEA | MW724160 | *[Neosetophoma samarorum](https://blast.ncbi.nlm.nih.gov/Blast.cgi)* | [MK247705.1](https://www.ncbi.nlm.nih.gov/nucleotide/MK247705.1?report=genbank&log$=nucltop&blast_rank=1&RID=C4BJ6D19014) | 928 | 100.00% | 928 | 99% | 0 |
| 226 | MAR | NANKAI B MEA | MW724161 | *[Dothiorella viticola](https://blast.ncbi.nlm.nih.gov/Blast.cgi)* | [KY385661.1](https://www.ncbi.nlm.nih.gov/nucleotide/KY385661.1?report=genbank&log$=nucltop&blast_rank=1&RID=C4BSEMAW014) | 959 | 100.00% | 959 | 100% | 0 |
| 227 | MAR | NANKAI B MEA | MW724162 | *[Phoma macrostoma](https://blast.ncbi.nlm.nih.gov/Blast.cgi)* | [KF293857.1](https://www.ncbi.nlm.nih.gov/nucleotide/KF293857.1?report=genbank&log$=nucltop&blast_rank=1&RID=C4C8XB6J014) | 926 | 99.79% | 926 | 100% | 0 |
| 228 | MAR | NANKAI B SDA | MW724163 | *[Aplosporella javeedii](https://blast.ncbi.nlm.nih.gov/Blast.cgi)* | [MH974687.1](https://www.ncbi.nlm.nih.gov/nucleotide/MH974687.1?report=genbank&log$=nucltop&blast_rank=1&RID=C4CD4XBU014) | 942 | 97.75% | 942 | 99% | 0 |
| 229 | MAR | NANKAI B SDA | MW724164 | *[Phoma](https://blast.ncbi.nlm.nih.gov/Blast.cgi)* [sp.](https://blast.ncbi.nlm.nih.gov/Blast.cgi) | [MN944537.1](https://www.ncbi.nlm.nih.gov/nucleotide/MN944537.1?report=genbank&log$=nucltop&blast_rank=1&RID=C4CHCDCS014) | 809 | 100.00% | 809 | 100% | 0 |
|  |  |  |  | *[Didymella glomerata](https://blast.ncbi.nlm.nih.gov/Blast.cgi)* | [MT000453.1](https://www.ncbi.nlm.nih.gov/nucleotide/MT000453.1?report=genbank&log$=nucltop&blast_rank=2&RID=C4CHCDCS014) | 807 | 100.00% | 807 | 99% | 0 |
| 230 | MAR | NANKAI B SDA | MW724165 | *[Alternaria](https://blast.ncbi.nlm.nih.gov/Blast.cgi)* [sp.](https://blast.ncbi.nlm.nih.gov/Blast.cgi) | [MT447542.1](https://www.ncbi.nlm.nih.gov/nucleotide/MT447542.1?report=genbank&log$=nucltop&blast_rank=1&RID=C4CRPRN1014) | 957 | 100.00% | 957 | 100% | 0 |
|  |  |  |  | *[Alternaria alternata](https://blast.ncbi.nlm.nih.gov/Blast.cgi)* | [MT446185.1](https://www.ncbi.nlm.nih.gov/nucleotide/MT446185.1?report=genbank&log$=nucltop&blast_rank=2&RID=C4CRPRN1014) | 957 | 100.00% | 957 | 100% | 0 |
| 231 | MAR | NANKAI B SDA | MW724166 | *[Aplosporella javeedii](https://blast.ncbi.nlm.nih.gov/Blast.cgi)* | [MH974687.1](https://www.ncbi.nlm.nih.gov/nucleotide/MH974687.1?report=genbank&log$=nucltop&blast_rank=1&RID=C4DSEN1X014) | 878 | 97.61% | 878 | 99% | 0 |
| 232 | MAR | NANKAI B SDA | MW724167 | *[Aplosporella javeedii](https://blast.ncbi.nlm.nih.gov/Blast.cgi)* | [MH974687.1](https://www.ncbi.nlm.nih.gov/nucleotide/MH974687.1?report=genbank&log$=nucltop&blast_rank=1&RID=C4E0VR2X016) | 934 | 97.74% | 934 | 99% | 0 |
| 233 | MAR | NANKAI B SDA | MW724168 | *[Spencermartinsia](https://blast.ncbi.nlm.nih.gov/Blast.cgi)* [sp.](https://blast.ncbi.nlm.nih.gov/Blast.cgi) | [KY290227.1](https://www.ncbi.nlm.nih.gov/nucleotide/KY290227.1?report=genbank&log$=nucltop&blast_rank=1&RID=C4E4M72X016) | 961 | 100.00% | 961 | 98% | 0 |
|  |  |  |  | *[Dothiorella viticola](https://blast.ncbi.nlm.nih.gov/Blast.cgi)* | [KY385661.1](https://www.ncbi.nlm.nih.gov/nucleotide/KY385661.1?report=genbank&log$=nucltop&blast_rank=2&RID=C4E4M72X016) | 961 | 99.80% | 961 | 99% | 0 |
| 234 | MAR | NANKAI B SDA | MW724169 | *[Alternaria](https://blast.ncbi.nlm.nih.gov/Blast.cgi)* [sp.](https://blast.ncbi.nlm.nih.gov/Blast.cgi) | [MT447542.1](https://www.ncbi.nlm.nih.gov/nucleotide/MT447542.1?report=genbank&log$=nucltop&blast_rank=1&RID=C4E8WB3E014) | 1011 | 100.00% | 1011 | 100% | 0 |
|  |  |  |  | *[Alternaria alternata](https://blast.ncbi.nlm.nih.gov/Blast.cgi)* | [MT446184.1](https://www.ncbi.nlm.nih.gov/nucleotide/MT446184.1?report=genbank&log$=nucltop&blast_rank=2&RID=C4E8WB3E014) | 1011 | 100.00% | 1011 | 100% | 0 |
| 235 | MAR | NANKAI B SDA | MW724170 | *[Dothiorella viticola](https://blast.ncbi.nlm.nih.gov/Blast.cgi)* | [KY385661.1](https://www.ncbi.nlm.nih.gov/nucleotide/KY385661.1?report=genbank&log$=nucltop&blast_rank=1&RID=C4EDNHDN016) | 959 | 100.00% | 959 | 100% | 0 |
| 236 | MAR | NANKAI B SDA | MW724171 | *Dothiorella viticola* | KY385661.1 | 890 | 100.00% | 890 | 100% | 0 |
| 237 | MAR | NANKAI B SDA | MW724172 | *Kazachstania humilis* | MN913450.1 | 1134 | 100.00% | 1134 | 100% | 0 |
| 238 | MAR | NANKAI B SDA | MW724173 | *[Aplosporella javeedii](https://blast.ncbi.nlm.nih.gov/Blast.cgi)* | [MH974687.1](https://www.ncbi.nlm.nih.gov/nucleotide/MH974687.1?report=genbank&log$=nucltop&blast_rank=1&RID=C4EZU3ZV014) | 902 | 97.67% | 902 | 99% | 0 |
| 239 | MAR | NANKAI B SDA | MW724174 | *[Aplosporella javeedii](https://blast.ncbi.nlm.nih.gov/Blast.cgi)* | [MH974687.1](https://www.ncbi.nlm.nih.gov/nucleotide/MH974687.1?report=genbank&log$=nucltop&blast_rank=1&RID=C4F6VKRN016) | 950 | 97.77% | 950 | 99% | 0 |
| 240 | MAR | NANKAI B SDA | MW724175 | *[Alternaria](https://blast.ncbi.nlm.nih.gov/Blast.cgi)* [sp.](https://blast.ncbi.nlm.nih.gov/Blast.cgi) | [MT447542.1](https://www.ncbi.nlm.nih.gov/nucleotide/MT447542.1?report=genbank&log$=nucltop&blast_rank=1&RID=C4FAYJ71014) | 954 | 100.00% | 954 | 100% | 0 |
|  |  |  |  | *[Alternaria alternata](https://blast.ncbi.nlm.nih.gov/Blast.cgi)* | [MT446185.1](https://www.ncbi.nlm.nih.gov/nucleotide/MT446185.1?report=genbank&log$=nucltop&blast_rank=2&RID=C4FAYJ71014) | 954 | 100.00% | 954 | 100% | 0 |
| 241 | MAR | NANKAI B SDA | MW724176 | *[Coniothyrium pyrinum](https://blast.ncbi.nlm.nih.gov/Blast.cgi)* | [MT126619.1](https://www.ncbi.nlm.nih.gov/nucleotide/MT126619.1?report=genbank&log$=nucltop&blast_rank=1&RID=C4FERNEP016) | 942 | 100.00% | 942 | 100% | 0 |

**BLAST search closest matches of fungal internal transcribed spacer DNA sequences amplified from Tianjin air borne fungi. In sample codes, sampling district name (Nankai, Hexi, Heping, Hebei), site (G = Green, B = Busy), and isolation medium (MEA = Malt Extract Agar, SDA = Sabouraud Dextrose Agar) are indicated. Sample GenBank accession codes, accession codes for the closest GenBank matches, sequence identity, and overlap of each match are reported.**

**Supplementary Table S4: Isolated airborne fungal genera and number of strains**

| **Fungal Genera** | **December** | **January** | **February** | **March** | **No. of Isolates** | **Percentage (%)** |
| --- | --- | --- | --- | --- | --- | --- |
| *Alternaria* | 16 | 12 | 48 | 47 | 123 | 21.96 |
| *Cladosporium* | 23 | 19 | 42 | 19 | 103 | 18.39 |
| *Naganishia* | 1 | 4 | 17 | 57 | 79 | 14.11 |
| *Fusarium* | 4 | 3 | 16 | 10 | 33 | 5.89 |
| *Didymella* | 4 | 3 | 10 | 10 | 27 | 4.82 |
| *Phoma* | 5 | 3 | 2 | 13 | 23 | 4.11 |
| *Epicoccum* | 7 | 2 | 1 | 7 | 17 | 3.04 |
| *Aureobasidium* | 0 | 1 | 2 | 11 | 14 | 2.50 |
| *Valsa* | 5 | 7 | 0 | 1 | 13 | 2.32 |
| *Aspergillus* | 3 | 5 | 2 | 0 | 10 | 1.79 |
| *Dothiorella* | 1 | 1 | 0 | 8 | 10 | 1.79 |
| *Paraconiothyrium* | 0 | 1 | 1 | 8 | 10 | 1.79 |
| *Talaromyces* | 0 | 0 | 0 | 10 | 10 | 1.79 |
| *Papiliotrema* | 0 | 0 | 8 | 1 | 9 | 1.61 |
| *Microsphaeropsis* | 0 | 2 | 1 | 5 | 8 | 1.43 |
| *Aplosporella* | 0 | 0 | 0 | 6 | 6 | 1.07 |
| *Colletotrichum* | 1 | 0 | 4 | 0 | 5 | 0.89 |
| *Filobasidium* | 2 | 3 | 0 | 0 | 5 | 0.89 |
| *Nothophoma* | 0 | 1 | 0 | 4 | 5 | 0.89 |
| *Rhodotorula* | 0 | 0 | 0 | 4 | 4 | 0.71 |
| *Sistotrema* | 4 | 0 | 0 | 0 | 4 | 0.71 |
| *Coniothyrium* | 0 | 1 | 0 | 2 | 3 | 0.54 |
| *Ectophoma* | 1 | 2 | 0 | 0 | 3 | 0.54 |
| *Erythrobasidium* | 1 | 0 | 2 | 0 | 3 | 0.54 |
| *Curvularia* | 0 | 1 | 1 | 0 | 2 | 0.36 |
| *Didymellaceae* | 0 | 0 | 0 | 2 | 2 | 0.36 |
| *Dioszegia* | 0 | 0 | 0 | 2 | 2 | 0.36 |
| *Dothideomyces* | 0 | 0 | 0 | 2 | 2 | 0.36 |
| *Kazachstania* | 0 | 0 | 1 | 1 | 2 | 0.36 |
| *Rhizopus* | 0 | 2 | 0 | 0 | 2 | 0.36 |
| *Symmetrospora* | 0 | 0 | 2 | 0 | 2 | 0.36 |
| *Vishniacozyma* | 0 | 0 | 0 | 2 | 2 | 0.36 |
| *Acrodontium* | 0 | 0 | 0 | 1 | 1 | 0.18 |
| *Allophoma* | 0 | 0 | 0 | 1 | 1 | 0.18 |
| *Bipolaris* | 0 | 0 | 1 | 0 | 1 | 0.18 |
| *Coreomyces* | 0 | 0 | 1 | 0 | 1 | 0.18 |
| *Cytospora* | 0 | 0 | 0 | 1 | 1 | 0.18 |
| *Daldinia* | 0 | 0 | 1 | 0 | 1 | 0.18 |
| *Herpotrichia* | 0 | 0 | 0 | 1 | 1 | 0.18 |
| *Lasiodiplodia* | 0 | 0 | 1 | 0 | 1 | 0.18 |
| *Massarina* | 0 | 1 | 0 | 0 | 1 | 0.18 |
| *Neosetophoma* | 0 | 0 | 0 | 1 | 1 | 0.18 |
| *Periconia* | 0 | 0 | 1 | 0 | 1 | 0.18 |
| *Phaeosphaeria* | 0 | 0 | 0 | 1 | 1 | 0.18 |
| *Candida* | 0 | 0 | 0 | 1 | 1 | 0.18 |
| *Purpureocillium* | 1 | 0 | 0 | 0 | 1 | 0.18 |
| *Stagonosporopsis* | 0 | 0 | 0 | 1 | 1 | 0.18 |
| *Thelebolus* | 0 | 0 | 0 | 1 | 1 | 0.18 |
| *Trichoderma* | 1 | 0 | 0 | 0 | 1 | 0.18 |

**Supplementary Table S5: Isolated air borne fungal species and number of strains**

| **Fungi** | **Dec** | **Jan** | **Feb** | **March** | **Total** | **Percentage (%)** |
| --- | --- | --- | --- | --- | --- | --- |
| *Acrodontium crateriforme* | 0 | 0 | 0 | 1 | 1 | 0.18 |
| *Allophoma labilis* | 0 | 0 | 0 | 1 | 1 | 0.18 |
| *Alternaria alternata* | 7 | 9 | 25 | 34 | 75 | 13.3 |
| *Alternaria arborescens* | 0 | 0 | 0 | 1 | 1 | 0.18 |
| *Alternaria brassicicola* | 0 | 1 | 0 | 0 | 1 | 0.18 |
| *Alternaria eichhorniae* | 1 | 0 | 0 | 0 | 1 | 0.18 |
| *Alternaria longipes* | 0 | 0 | 1 | 0 | 1 | 0.18 |
| *Alternaria porri* | 0 | 0 | 0 | 1 | 1 | 0.18 |
| *Alternaria* sp. | 0 | 0 | 0 | 1 | 1 | 0.18 |
| *Alternaria tenuissima* | 0 | 2 | 22 | 10 | 34 | 6.07 |
| *Aplosporella javeedii* | 0 | 0 | 0 | 6 | 6 | 1.07 |
| *Aspergillus flavus* | 0 | 1 | 1 | 0 | 2 | 0.36 |
| *Aspergillus niger* | 0 | 0 | 1 | 0 | 1 | 0.18 |
| *Aspergillus nomius* | 1 | 1 | 0 | 0 | 2 | 0.36 |
| *Aspergillus ochraceus* | 1 | 0 | 0 | 0 | 1 | 0.18 |
| *Aspergillus ostianus* | 0 | 3 | 0 | 0 | 3 | 0.54 |
| *Aspergillus pseudoglaucus* | 1 | 0 | 0 | 0 | 1 | 0.18 |
| *Aureobasidium namibiae* | 0 | 0 | 0 | 2 | 2 | 0.36 |
| *Aureobasidium proteae* | 0 | 0 | 0 | 3 | 3 | 0.54 |
| *Aureobasidium pullulans* | 0 | 1 | 2 | 5 | 8 | 1.43 |
| *Aureobasidium* sp. | 0 | 0 | 0 | 1 | 1 | 0.18 |
| *Bipolaris zeae* | 0 | 0 | 1 | 0 | 1 | 0.18 |
| *Candida auris* | 0 | 0 | 0 | 1 | 1 | 0.18 |
| *Cladosporium anthropophilum* | 1 | 0 | 3 | 6 | 10 | 1.79 |
| *Cladosporium asperulatum* | 1 | 2 | 1 | 0 | 4 | 0.71 |
| *Cladosporium cladosporioides* | 10 | 13 | 30 | 1 | 54 | 9.64 |
| *Cladosporium gossypiicola* | 0 | 0 | 0 | 1 | 1 | 0.18 |
| *Cladosporium herbarum* | 0 | 0 | 0 | 6 | 6 | 1.07 |
| *Cladosporium perangustum* | 2 | 0 | 2 | 0 | 4 | 0.36 |
| *Cladosporium pseudocladosporioides* | 4 | 0 | 0 | 0 | 4 | 0.71 |
| *Cladosporium ramotenellum* | 0 | 1 | 0 | 1 | 2 | 0.36 |
| *Cladosporium* sp. | 2 | 1 | 1 | 3 | 7 | 1.25 |
| *Cladosporium subuliforme* | 1 | 1 | 0 | 0 | 2 | 0.36 |
| *Cladosporium tenuissimum* | 8 | 0 | 4 | 1 | 13 | 2.32 |
| *Cladosporium uredinicola* | 0 | 0 | 1 | 0 | 1 | 0.18 |
| *Cladosporium xanthochromaticum* | 2 | 1 | 0 | 0 | 3 | 0.54 |
| *Colletotrichum gloeosporioides* | 1 | 0 | 4 | 1 | 6 | 1.07 |
| *Coniothyrium aleuritis* | 0 | 1 | 0 | 1 | 2 | 0.36 |
| *Coniothyrium pyrinum* | 0 | 0 | 0 | 1 | 1 | 0.18 |
| *Coreomyces* sp. | 0 | 0 | 1 | 0 | 1 | 0.18 |
| *Curvularia* sp. | 0 | 0 | 1 | 0 | 1 | 0.18 |
| *Curvularia tsudae* | 0 | 1 | 0 | 0 | 1 | 0.18 |
| *Cytospora chrysosperma* | 0 | 0 | 0 | 1 | 1 | 0.18 |
| *Daldinia eschscholtzii* | 0 | 0 | 1 | 0 | 1 | 0.18 |
| *Didymella bryoniae* | 0 | 0 | 0 | 1 | 1 | 0.18 |
| *Didymella glomerata* | 0 | 0 | 2 | 0 | 2 | 0.36 |
| *Didymella macrostoma* | 0 | 0 | 2 | 1 | 3 | 0.54 |
| *Didymella pedeiae* | 2 | 1 | 6 | 6 | 15 | 2.68 |
| *Didymella* sp. | 0 | 2 | 0 | 0 | 2 | 0.36 |
| *Didymella subherbarum* | 2 | 0 | 0 | 0 | 2 | 0.36 |
| *Didymellaceae* sp. | 0 | 0 | 0 | 2 | 2 | 0.36 |
| *Dioszegia zsoltii* | 0 | 0 | 0 | 2 | 2 | 0.36 |
| *Dothideomycetes* sp. | 0 | 0 | 0 | 2 | 2 | 0.36 |
| *Dothiorella gregaria* | 1 | 0 | 0 | 2 | 3 | 0.54 |
| *Dothiorella viticola* | 0 | 1 | 0 | 6 | 7 | 1.25 |
| *Ectophoma multirostrata* | 1 | 2 | 0 | 0 | 3 | 0.54 |
| *Epicoccum latusicollum* | 2 | 0 | 0 | 0 | 2 | 0.36 |
| *Epicoccum nigrum* | 0 | 1 | 0 | 7 | 8 | 1.43 |
| *Epicoccum sorghinum* | 5 | 1 | 1 | 0 | 7 | 1.25 |
| *Erythrobasidium hasegawianum* | 1 | 0 | 2 | 0 | 3 | 0.54 |
| *Filobasidium chernovii* | 1 | 0 | 0 | 0 | 1 | 0.18 |
| *Filobasidium magnum* | 1 | 2 | 0 | 0 | 3 | 0.54 |
| *Filobasidium uniguttulatum* | 0 | 1 | 0 | 0 | 1 | 0.18 |
| *Fusarium cf. incarnatum-equiseti* | 2 | 0 | 0 | 0 | 2 | 0.36 |
| *Fusarium chlamydosporum* | 1 | 0 | 0 | 0 | 1 | 0.18 |
| *Fusarium equiseti* | 1 | 3 | 0 | 9 | 13 | 2.32 |
| *Fusarium oxysporum* | 0 | 0 | 1 | 0 | 1 | 0.18 |
| *Fusarium tricinctum* | 0 | 0 | 0 | 1 | 1 | 0.18 |
| *Fusarium verticillioides* | 0 | 0 | 15 | 0 | 15 | 2.68 |
| *Herpotrichia striatispora* | 0 | 0 | 0 | 1 | 1 | 0.18 |
| *Kazachstania humilis* | 0 | 0 | 1 | 1 | 2 | 0.36 |
| *Lasiodiplodia theobromae* | 0 | 0 | 1 | 0 | 1 | 0.18 |
| *Massarina igniaria* | 0 | 1 | 0 | 0 | 1 | 0.18 |
| *Microsphaeropsis olivacea* | 0 | 2 | 1 | 5 | 8 | 1.43 |
| *Naganishia adeliensis* | 0 | 0 | 0 | 1 | 1 | 0.18 |
| *Naganishia albida* | 0 | 1 | 17 | 18 | 36 | 6.43 |
| *Naganishia friedmannii* | 0 | 0 | 0 | 1 | 1 | 0.18 |
| *Naganishia globosa* | 1 | 3 | 0 | 30 | 34 | 6.07 |
| *Naganishia liquefaciens* | 0 | 0 | 0 | 2 | 2 | 0.34 |
| *Naganishia randhawae* | 0 | 0 | 0 | 1 | 1 | 0.18 |
| *Naganishia uzbekistanensis* | 0 | 0 | 0 | 3 | 3 | 0.54 |
| *Neosetophoma samarorum* | 0 | 0 | 0 | 1 | 1 | 0.18 |
| *Nothophoma quercina* | 0 | 1 | 0 | 0 | 1 | 0.18 |
| *Nothophoma* sp. | 0 | 0 | 0 | 4 | 4 | 0.71 |
| *Papiliotrema flavescens* | 0 | 0 | 8 | 0 | 8 | 1.43 |
| *Papiliotrema laurentii* | 0 | 0 | 0 | 2 | 2 | 0.36 |
| *Paraconiothyrium archidendri* | 0 | 0 | 0 | 1 | 1 | 0.18 |
| *Paraconiothyrium hawaiiense* | 0 | 1 | 1 | 8 | 10 | 1.79 |
| *Periconia epilithographicola* | 0 | 0 | 1 | 0 | 1 | 0.18 |
| *Phaeosphaeria* sp. | 0 | 0 | 0 | 1 | 1 | 0.18 |
| *Phoma herbarum* | 1 | 0 | 0 | 2 | 3 | 0.54 |
| *Phoma macrostoma var. macrostoma* | 0 | 1 | 0 | 4 | 5 | 0.89 |
| *Phoma medicaginis* | 2 | 0 | 0 | 3 | 5 | 0.89 |
| *Phoma multirostrata* | 1 | 2 | 2 | 0 | 5 | 0.89 |
| *Phoma sojicola* | 0 | 0 | 0 | 1 | 1 | 0.18 |
| *Phoma* sp. | 1 | 0 | 0 | 3 | 4 | 0.71 |
| *Purpureocillium lilacinum* | 1 | 0 | 0 | 0 | 1 | 0.18 |
| *Rhizopus oryzae* | 0 | 2 | 0 | 0 | 2 | 0.36 |
| *Rhodotorula mucilaginosa* | 0 | 0 | 0 | 4 | 4 | 0.71 |
| *Sistotrema brinkmannii* | 4 | 0 | 0 | 0 | 4 | 0.71 |
| *Stagonosporopsis cucurbitacearum* | 0 | 0 | 0 | 1 | 1 | 0.18 |
| *Symmetrospora foliicola* | 0 | 0 | 1 | 0 | 1 | 0.18 |
| *Symmetrospora symmetrica* | 0 | 0 | 1 | 0 | 1 | 0.18 |
| *Talaromyces funiculosus* | 0 | 0 | 0 | 4 | 4 | 0.71 |
| *Talaromyces marneffei* | 0 | 0 | 0 | 6 | 6 | 1.07 |
| *Thelebolus microsporus* | 0 | 0 | 0 | 1 | 1 | 0.18 |
| *Trichoderma asperellum* | 1 | 0 | 0 | 0 | 1 | 0.18 |
| *Valsa sordida* | 5 | 7 | 0 | 1 | 13 | 2.32 |
| *Vishniacozyma* sp. | 0 | 0 | 0 | 1 | 1 | 0.18 |
| *Vishniacozyma tephrensis* | 0 | 0 | 0 | 1 | 1 | 0.18 |
